# Characterizing device-measured sleep in observational health research using compositional data analysis: a systematic review

**DOI:** 10.1186/s12966-026-01935-8

**Published:** 2026-06-15

**Authors:** Christine W. St. Laurent, Guilherme Moraes Balbim, Denver M. Y. Brown, Chelsea L. Kracht, Christopher D. Pfledderer, Claire I. Groves, Carah D. Holesovsky, Griffin A. T. Randolph, Katrina Rodheim, Sarah Burkart

**Affiliations:** 1 Department of Kinesiology, University of Massachusetts Amherst, Amherst, Massachusetts USA; 2 Department of Physical Therapy, The University of British Columbia, Vancouver, British Columbia Canada; 3 School of Health Sciences, Kansas State University, Manhattan, Kansas USA; 4 Department of Internal Medicine, Medical Center, University of Kansas, Kansas City, Kansas USA; 5 The University of Texas Health Science Center Houston, School of Public Health in Austin, Austin, Texas USA; 6 Department of Psychology, University of Texas at San Antonio, San Antonio, Texas USA; 7 University of South Carolina, Arnold School of Public Health, Columbia, South Carolina USA

**Keywords:** Physical activity, Sedentary behavior, Nap, Actigraphy, Time-use

## Abstract

**Abstract** Compositional data analysis (CoDA) is widely used to examine the associations between one’s balance of movement behaviors (sedentary behavior, physical activity, and sleep) and health outcomes. Existing reviews have primarily focused on reporting standards for physical activity and sedentary behavior, but have not considered the contribution of sleep reporting to our understanding of CoDA.

**Purpose** To characterize device-based sleep data measurement, processing, and reporting in studies using CoDA to examine associations between movement behaviors and health indicators.

**Methods** A systematic search was conducted in seven databases, along with supplemental strategies (forward and backward citation searches and expert review). Observational studies published since 2015 that employed CoDA approaches using isometric log-ratio transformations to examine the associations between movement behavior compositions utilizing device-based measures of sleep and health outcomes were included. Data extraction included items based on sleep actigraphy measurement, processing, and reporting practices recommended by the American Academy of Sleep Medicine. The National Institutes of Health Quality Assessment Tool for Observational Cohort and Cross-sectional Studies was used to assess study quality.

**Results** Among the 70 included studies (*n* = 60 cross-sectional, *n* = 10 longitudinal), few articles reported key sleep measurement and processing information. Most articles (*n* = 67) included only one sleep component in the time-use composition, and less than half of the articles (*n* = 29) acknowledged sleep-related limitations. Reports were classified as having good (*n* = 60) or poor (*n* = 10) study quality.

**Conclusions** This review identified inconsistencies in the measurement, processing, and reporting of device-measured sleep in studies using CoDA. Varying protocols and reporting on sleep data processing highlight the need for adoption of current standardized approaches and reporting practices. Future research should prioritize transparency and consistency to improve the validity and comparability of findings on sleep’s role as a key component of the integrative 24-h approach to health.

**Supplementary Information:**

The online version contains supplementary material available at 10.1186/s12966-026-01935-8.

## Background

Current frameworks, such as the Framework for Viable Integrative Research in Time-Use Epidemiology (VIRTUE), have recognized the interactive nature and mutual influence of behaviors within a 24-h cycle (e.g., sedentary behavior, physical activity, and sleep) and their impact on health across the lifespan [1, 2]. These frameworks emphasize the integration of ‘movement behaviors’ into a cohesive model that supports understanding their combined effects on health outcomes [2]. Indeed, recent reviews have highlighted the growing adoption and relevance of the integrative 24-h movement behavior approach [3–7]. The utilization of measurement and statistical analysis approaches, such as compositional data analysis (CoDA), that account for the full spectrum of daily behaviors and acknowledge their co-dependence, is recommended [8].

Compositional data analysis is a common statistical approach for analyzing 24-h movement data where the components represent parts of a whole and carry only relative information, such as proportions or percentages [9]. Due to the co-dependent nature, methods are needed to account for the compositional properties of the time-use data before applying standard statistical tests to better capture the relationships of movement behaviors with health outcomes [8]. In the context of 24-h movement behaviors, CoDA allows researchers to examine how reallocating time between sleep, sedentary behavior, and physical activity is associated with health outcomes by commonly using isometric log-ratio (ilr) transformations to convert the constrained data into orthogonal coordinates suitable for standard regression models and isotemporal substitution [10–12].

Alongside the application of compositional data analysis to interpret the interdependent nature of time-use data, researchers are increasingly incorporating the use of wearable devices to estimate behaviors of the 24-h cycle [1, 13]. Wearable devices, particularly wrist-worn monitors, are widely used to measure sleep in both research and clinical settings, offering a range of sleep-relevant metrics that provide insights into both the duration and sleep quality indicators such as wake after sleep onset (WASO), sleep onset latency, and sleep efficiency (percent of “time in bed” spent asleep) [14–16]. In the context of sleep science, actigraphy often refers to estimates of sleep–wake patterns primarily derived from wearable accelerometers [15, 17].

Actigraphy was originally developed as a practical alternative to polysomnography (PSG) for multi-day or ambulatory sleep monitoring and has been used for several decades [16, 18, 19]. Standardized approaches for using actigraphy in research studies were necessary, and guidelines were developed by organizations such as the Society of Behavioral Sleep Medicine (SBSM) and the American Academy of Sleep Medicine (AASM), which recognize its validity for estimating sleep duration, timing, and efficiency—particularly in populations without complex sleep disorders [15, 17]. These recommendations were developed for both clinical and research applications, but have been widely applied in epidemiologic and public health studies [14].

Recent reviews have acknowledged that most movement behavior research has concentrated on physical activity and sedentary behavior, often overlooking the complexities of sleep measurement and reporting [3, 4, 20]. For example, Brown et al. [3] noted that approximately only one-third of studies including sleep in the composition clearly defined ‘sleep.’ Without clear definitions, it is possible that researchers may be measuring ‘time in bed’ but drawing conclusions about ‘sleep duration.’ This is one example with implications that could ultimately (mis)inform future guideline development [16]. There are established recommendations for processing and reporting sleep metrics by the SBSM and AASM [15], but these may not be common knowledge for those outside the sleep field. Efforts to improve knowledge and expectations are clearly needed to provide consistent measurement and reporting in the field so as not to undermine knowledge gleaned from this integrative 24-h approach to health.

Much like physical activity and sedentary behavior, sleep is a complex behavior necessitating detailed measurement, processing, and results reporting. This review takes the first step to understanding sleep measures in CoDA studies by characterizing the landscape, intending to identify targets for improving future research with device-based measures of sleep within the context of 24-h movement behaviors. This systematic review aimed to characterize device-measured sleep measurement, processing, and reporting in studies that used CoDA to examine associations between 24-h movement behaviors and health. Specifically, in alignment with both actigraphy measurement and reporting recommendations [15, 17], we categorized such characteristics into the categories of device technical settings, sleep period recording, sleep conceptualization, sleep integration in time-use compositions, and discussion and transparency.

## Methods

### Protocol and registration

This review was registered a priori with the International Prospective Register of Systematic Reviews (PROSPERO; submitted July 29, 2024; ID: CRD42024568636). The current report follows the Preferred Reporting Items for Systematic Reviews and Meta-Analyses (PRISMA) guidelines [21] (Additional File 1).

### Eligibility criteria

Empirical original research studies were included if they (a) reported on human participants of any age, (b) were published between January 1, 2015 (as this was the year of the first publication examining 24-h movement behaviors in relation to health outcomes using CoDA) [22] and September 12, 2024, (c) were published in English, (d) used an observational design (e.g., cross-sectional or longitudinal), and (e) were published in peer-reviewed journals. The exposure of included studies was a daily time use composition that used ilr transformations, as this is a standard and common CoDA approach for handling the co-dependent nature of 24-h movement behaviors [3]. To align with the review's focus on sleep, included studies were required to report at least one free-living, device-measured, time-based (i.e., duration) sleep metric, such as those obtained from actigraphy, accelerometry, or ambulatory polysomnography, in addition to measures of physical activity and sedentary behavior. Inclusion criteria did not have limits regarding specific days of the week (e.g., weekdays or weekends) or a minimum number of compositional components. Finally, studies were included if they reported at least one non-behavioral health outcome, categorized as either physical (e.g., obesity, diabetes risk, mortality) or mental/brain (e.g., psychological well-being, executive function, brain structure), to capture the breadth of health domains associated with movement behaviors.

We excluded studies that were (a) published as gray literature, (b) used qualitative, case study/case series, or experimental study designs, (c) were review, commentary, or consensus reports, or (d) aimed to describe an approach, method, or study protocol.

### Information sources and search strategy

A systematic search strategy was developed in consultation with a research librarian. We developed a list of terms (Additional File 2) that were entered into an electronic search of seven databases (PubMed, PsycINFO, Sport Discus, CINAHL, Web of Science, Scopus, and Cochrane Library) conducted on September 12, 2024. Supplementary sources included articles published within the time frame from a movement behavior-specific journal (*Journal of Activity, Sedentary, and Sleep Behaviors)*, in addition to publications listed online by the International Network of Time Use Epidemiologists (INTUE). The other supplementary search approaches included backwards and forwards citation of included articles, and consulting experts in the field to review our list of included studies and identify any missing relevant articles.

### Selection process

Article screening and data extraction were conducted utilizing Covidence software (Evidence Partners, Ottawa, ON, Canada). After uploading initial searches, duplicates were auto-detected and removed. No automation tools were used for screening. Abstracts and full-text articles were screened by two independent reviewers (all authors). A standardized protocol was developed in advance for both screening stages, requiring reviewers to reach at least 80% accuracy before proceeding. A third reviewer resolved conflicts for both screening phases, when applicable.

### Data extraction

Data were extracted for each included study by two independent reviewers. First, general article data were extracted, including publication information (first author, publication year, country(ies)), participating information (sample size, age, sex), study design, health outcome(s), and 24-h movement behavior measurement information.

For overall descriptive information, data were extracted for the following categories: general information, participant characteristics, study characteristics, protocol, outcomes, movement behavior measures, CoDA, discussion, and other study details. In accordance with established recommendations for actigraphy measurement and reporting, we organized the extracted information into five categories: device technical settings, sleep period recording, sleep conceptualization, integration of sleep within time-use compositions, and reporting transparency and discussion (Table 1) [15, 17]. Data for each article were extracted by two independent reviewers (all authors), and a third reviewer (CWS or SB) compared the data for consensus.

**Table 1:** Data extraction categories, descriptions, and extraction items that were used to synthesize and characterize device-measured sleep measurement, processing, and reporting

Category	Description	Extraction Items
Device Technical Settings	Information about the devices used to measure sleep	Type of device; Device placement; Data collection device parameters and settings; Scoring and processing method(s) (i.e., visual inspection, manual scoring, software, algorithm and/or code, sleep log, event marker, and/or light sensor)
Sleep Period Recording	Specifics of the wear period	How long the device was worn; Minimum valid days required for analysis; How valid days were defined; How non-wear time was defined
Sleep Conceptualization	How sleep was conceptualized and measured	How sleep was defined (e.g., nighttime sleep, 24-h sleep); How sleep was classified into time-based metrics (e.g., total sleep time or sleep duration, time in bed, sleep onset latency, and wake after sleep onset); How authors defined sleep metrics
Sleep Integration in Time-Use Compositions	How sleep was integrated into the time-use composition models used in the study	How the composition was closed (e.g., closed to 24 h or sample mean wear time); Number of time-use components; Number of sleep components
Discussion & Transparency	How sleep was discussed in the context of the study	If authors addressed limitations of sleep measurement; If statistical code was shared or made available*

^*^Included for descriptive purposes, but not a component of the data synthesis score

### Data synthesis

In addition to providing a narrative synthesis for each of the categories in Table 1, for each individual component, scores of ‘2’ were assigned for ‘yes’ (i.e., having the information), ‘1’ for unclear (i.e., partially reported information or available upon request but not included in the article), and ‘0’ if information was not provided. Scores were summed by category and overall, and supplemented with a qualitative synthesis of results.

### Methodological quality and risk of bias assessment

The National Institutes of Health Quality Assessment Tool for Observational Cohort and Cross-sectional Studies assessed study quality and risk of bias [23]. This assessment consists of 14 questions with five response options: “yes”, “no”, “cannot determine”, “not applicable”, and “not reported”. Items with “yes” responses were assigned a score of 1, with other responses assigned zero. The final score for each article was the sum of all items. Although the tool was designed as a guide to align our findings with other work [3, 24], scores were classified into the following categories: “poor” (4 or less), “fair” (5 to 8), or “good” (9 or higher).

## Results

### Included studies

**Fig. 1:**
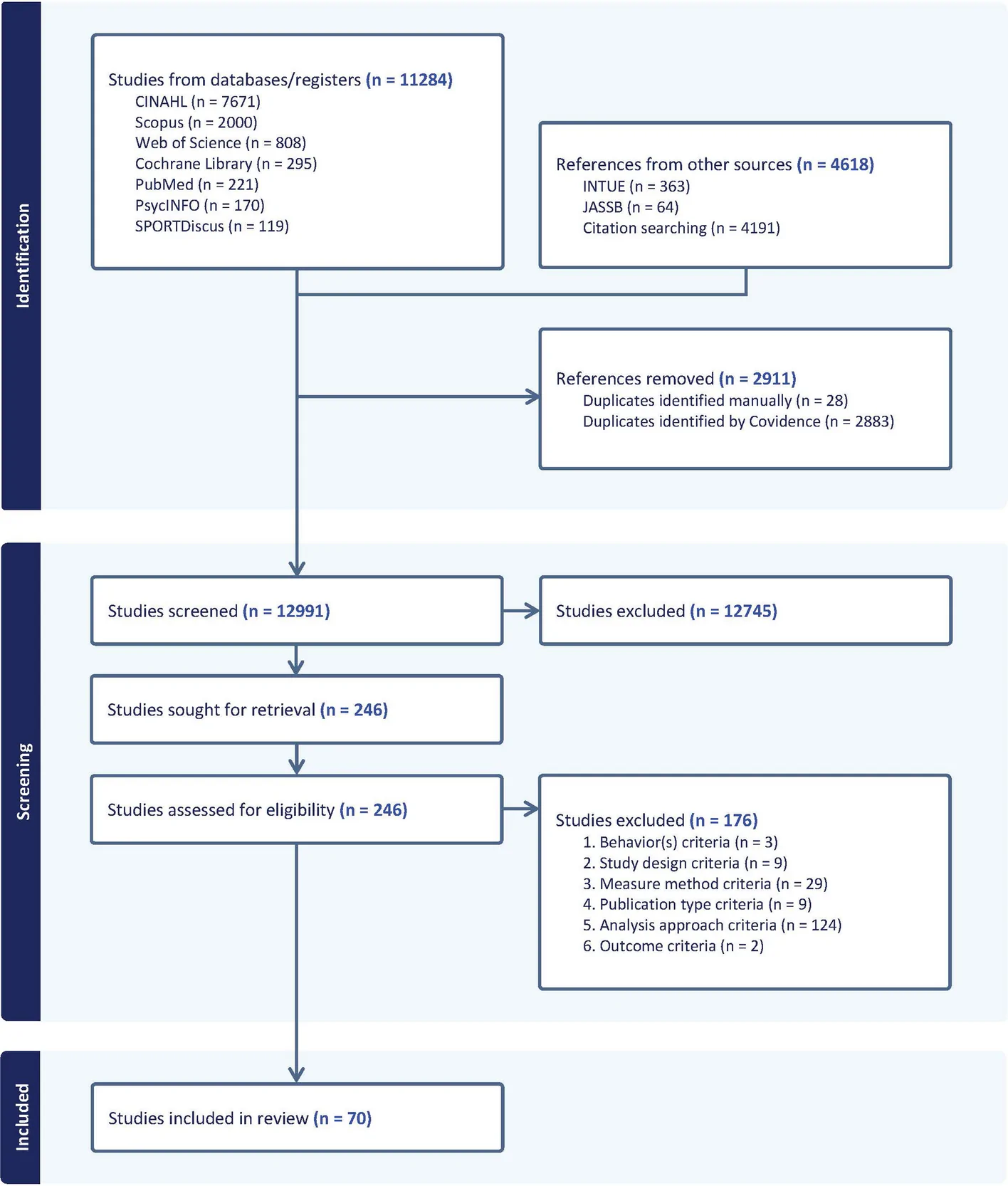
PRISMA 2022 main checklist

The initial database search resulted in 11,284 titles (Fig. 1). Supplemental searches added 4,618 titles. Once duplicates were removed (*n* = 2,911), 12,991 remained for screening. After initial screening at the title and abstract level, 246 full-text articles were reviewed. One hundred seventy-six articles were excluded, with the most common reasons including not meeting the analysis approach criteria (e.g., did not use CoDA), followed by an ineligible measurement of sleep (e.g., only used self-report methods) (Additional File 3). Seventy articles met the inclusion criteria and were included in this review [25–94].

A summary of the general characteristics of the included articles is presented in Table 2. Articles were published between 2017 and 2024, with 52% published between 2022 and 2024. Twenty-nine countries were represented among the study participants, with 12 articles (17.1%) comprising multi-country samples, and most studies originated from Australia (*n* = 21). Among the 15 articles that indicated the specific data source, parent studies or cohorts included the Longitudinal Study of Australian Children (LSAC) (*n* = 5), 1970 British Cohort Study (*n* = 2), ACTIVate study (*n* = 2), Observation of Cardiovascular Risk Factors study (ORISCAV-LUX 2) (*n* = 2), Rotterdam Study (*n* = 2), and Prevention of Overweight in Infancy study (POI) (*n* = 1). The range of analytic sample sizes (largest per article) was 25 to 87,498 participants. Thirty-six articles included children under age 6 years (51.4%), 13 included adolescents aged 13 to 17 years (18.6%), and 34 included adults (48.6%), with some studies including multiple age groups (*n* = 14; 20%). Sixty articles used solely cross-sectional designs, three presented only longitudinal analyses, and seven included both cross-sectional and longitudinal data.

**Table 2:** Characteristics of the included articles

First Author & Year	Year	Country(ies)	Age	Sex	Study Design	Sample Size	Health Outcome(s)	SB Measurement	PA Measurement	Sleep Device
Balbim 2024 [25]	2024	Canada	73.7 (5.41) years	62.1% female	Cross-sectional	253	Cognition	Motion Watch8	Motion Watch8	Motion Watch8
Bianchim 2022 [26]	2022	South Wales Australia	Children: 13.6 (2.8) years Adults: 24.6 (4.7) years	Children: 47.7% girls Adults: 48.8% female	Cross-sectional	Children: 86 Adults: 43	Cardiometabolic	ActiGraph GT9X Link GENEActiv	ActiGraph GT9X Link GENEActiv	ActiGraph GT9X Link GENEActiv
Biddle 2018 [27]	2018	UK	66.9 (7.4) years	38.3% female	Cross-sectional	435	Cardiometabolic	activPAL3	activPAL3	activPAL3
Blodgett 2023 [28]	2023	England Scotland Wales	46 years	52.3% female	Cross-sectional	4,738	Depression	activPAL3 micro	activPAL3 micro	activPAL3 micro
Blodgett 2024 [29]	2023	Denmark Finland Norway The Netherlands UK	53.7 (9.7) years	54.7% female	Cross-sectional	15,253	WBA Cardiometabolic	ActivPAL3/4 ActiGraph	ActivPAL3/4 ActiGraph	ActivPAL3/4 ActiGraph
Brakenridge 2021 [30]	2021	Australia	56.0 (9.8) years	52.7% female	Cross-sectional	FPG: 647 2hPLG: 640	Cardiometabolic	activPAL3	activPAL3	activPAL3
Brakenridge 2024 [31]	2024	Netherlands	60.1 (8.1) years	48.7% female	Cross-sectional	2,388	Cardiometabolic	activPAL3	activPAL3	activPAL3
Brayton 2023 [32]	2024	USA	15.8 (1.2) years	52% female	Longitudinal	33	Injury(ies)	ActiGraph GT9X Link	ActiGraph GT9X Link	ActiGraph GT9X Link
Cabanas-Sánchez 2021 [33]	2021	Spain	Cross-sectional: 71.7 (4.3) years Prospective: 71.4 (4.2) years	Cross-sectional: 53.07% female Prospective: 51.70% female	Cross-sectional Longitudinal	Cross-sectional: 2,489 Prospective: 1,679	Bone health Depression Other	ActiGraph GT9X	ActiGraph GT9X	ActiGraph GT9X
Chen 2023 [34]	2023	China	11.9 (2.1) years	50.13% female	Cross-sectional	389	WBA Cardiometabolic	ActiGraph wGT3X-BT	ActiGraph wGT3X-BT	ActiGraph wGT3X-BT
Chong 2021 [35]	2021	Australia	Cross-sectional: 11.7 (0.5) years Longitudinal: T1 = 11.8 (0.4) years, T2 = 12.8 (0.4) years	Cross-sectional: 73% female Longitudinal: 52% female	Cross-sectional Longitudinal	Baseline: 127 Follow up: 88	Prosocial behavior Behavioral conduct	GENEActiv	GENEActiv	GENEActiv
Collings 2023 [36]	2023	Luxembourg	51.2 (12.2) years	54% female	Cross-sectional	1,046	WBA Cardiometabolic	ActiGraph GT3X +	ActiGraph GT3X +	ActiGraph GT3X +
Collings 2023 [37]	2023	Luxembourg	50.6 (12.2) years	53.4% female	Cross-sectional	1,001	Cardiometabolic WBA Fitness	ActiGraph GT3X +	ActiGraph GT3X +	ActiGraph GT3X +
Curtis 2020 [38]	2020	Australia	41.3 (11.7) years	74% female	Cross-sectional	430	WBA HRQoL Depression Anxiety Stress	GENEActiv	GENEActiv	GENEActiv
Curtis 2023 [39]	2023	Australia	40.4 (5.9) years	58.1% female	Cross-sectional	322	Depression Anxiety Stress	Fitbit Charge 3	Fitbit Charge 3	Fitbit Charge 3
deFaria 2022 [40]	2022	Brazil	16.0 years	49.3% female	Cross-sectional	217	Depression Anxiety	ActiGraph GT3X	ActiGraph GT3X	ActiGraph GT3X
Domingues 2022 [41]	2022	Brazil	15.9 (1.0) years	49.2% female	Cross-sectional	185	WBA	ActiGraph GT3X	ActiGraph GT3X	ActiGraph GT3X
Dumuid 2017 [42]	2017	Australia	9 to 11 years	53.9% female	Cross-sectional	284	AAP	ActiGraph GT3X +	ActiGraph GT3X +	ActiGraph GT3X +
Dumuid 2018 [43]	2018	Australia	65.4 (2.9) years	61% female	Cross-sectional	122	WBA Cardiometabolic Fitness	ActiGraph GT3X +	ActiGraph GT3X +	ActiGraph GT3X +
Dumuid 2018 [44]	2018	Australia Brazil Canada China Colombia England Finland India Kenya Portugal South Africa USA	9 to 11 years	39 to 52% male	Cross-sectional	5,855	HRQoL	ActiGraph GT3X +	ActiGraph GT3X +	ActiGraph GT3X +
Dumuid 2018 [45]	2018	Australia Canada UK Finland	9 to 11 years	56.1% female	Cross-sectional	1,728	WBA	ActiGraph GT3X +	ActiGraph GT3X +	ActiGraph GT3X +
Dumuid 2019 [46]	2019	Australia	11.9 (0.4) years	50% female	Cross-sectional	971	WBA	GENEActiv	GENEActiv	GENEActiv
Dumuid 2020 [47]	2020	Australia	11.9 (0.4) years	50.4% female	Cross-sectional	66% site: 804 4% site: 767	Bone health	GENEActiv	GENEActiv	GENEActiv
Dumuid 2021 [48]	2021	Australia	12.0 (0.4) years	49% female	Cross-sectional	809 to 1,182	WBA Fitness	GENEActiv	GENEActiv	GENEActiv
Dumuid 2022 [49]	2022	Australia	65.6 (7.5) years	59.8% female	Cross-sectional	82	Cognition	GENEActiv	GENEActiv	GENEActiv
Dumuid 2022 [50]	2022	Australia	12.0 (0.4) years	49% female	Cross-sectional	1,182	WBA Cardiometabolic Bone health Fitness AAP ER Other	GENEActiv	GENEActiv	GENEActiv
Fairclough 2017 [51]	2017	England	10.3 (0.3) years	50.3% girls	Cross-sectional	169	WBA Fitness	ActiGraph GT9X	ActiGraph GT9X	ActiGraph GT9X
Fairclough 2021 [52]	2021	England	11.5 (1.4) years	50.7% girls	Cross-sectional	359	Depression Cognition ER Prosocial behavior Behavioral conduct Other	ActiGraph GT9X	ActiGraph GT9X	ActiGraph GT9X
Fairclough 2023 [53]	2023	England	11.1 (1.6) years	60.1% female	Cross-sectional	301	Behavioral conduct	ActiGraph GT9X	ActiGraph GT9X	ActiGraph GT9X
Gupta 2020 [54]	2020	Denmark	45.1 (9.7) years	45.6% female	Cross-sectional	807	WBA	ActiGraph GT3X +	ActiGraph GT3X +	ActiGraph GT3X +
Healy 2021 [55]	2021	USA	13.7 (2.97) years	23.3% female	Cross-sectional	46	WBA	ActiGraph GT9X Link	ActiGraph GT9X Link	ActiGraph GT9X Link
Hofman 2022 [56]	2022	Netherlands	70.9 (9.3) years	51.6% women	Cross-sectional	1,943	Depression Anxiety	GENEActiv	GENEActiv	GENEActiv
Janda 2024 [57]	2024	Czech Republic	Children: 11.6 (1.6) years Adolescents: 16.4 (1.3) years	57.7% female	Cross-sectional	Children: 374 Adolescents: 317	WBA	ActiGraph GT3X + GT9X Link	ActiGraph GT3X + GT9X Link	Children: ActiGraph GT3X + Adolescents: GT9X Link
Johansson 2020 [58]	2020	Denmark	Adults median: 48.3 years Older adults median: 72.7 years	Adults: 58.7% women Older adults: 55% women	Cross-sectional	Adults: 773 Older adults: 280	WBA Cardiometabolic	ActiGraph GT3X +	ActiGraph GT3X +	ActiGraph GT3X +
Kim 2021 [59]	2021	USA	50.1 (12.5) years	57.3% female	Cross-sectional	1,247	WBA	24-h Recall SenseWear Armband	24-h Recall SenseWear Armband	SenseWear Armband
Kuzik 2020 [60]	2020	Canada	4.5 years	69.5% male	Cross-sectional	95	WBA Motor skill(s) Cognition Emotional regulation Prosocial behavior Other	ActiGraph wGT3X-BT	ActiGraph wGT3X-BT	ActiGraph wGT3X-BT
Le 2022 [61]	2022	Australia	22.6 (5.3) years	72.6% female	Cross-sectional	361	Other	ActiGraph wGT3X-BT	ActiGraph wGT3X-BT	ActiGraph wGT3X-BT
Lee 2020 [62]	2020	USA	73.3 (1.7) years	100% women	Cross-sectional	136	WBA Cardiometabolic	ActiGraph GT1M	ActiGraph GT1M	Actiwatch-2
Lu 2024 [63]	2024	China	12.9 (2.0) years	56% girls	Cross-sectional	275	Prosocial behavior Behavioral conduct	ActiGraph GT3X +	ActiGraph GT3X +	ActiGraph GT3X +
Marmol-Perez 2024 [64]	2024	Spain	12.1 (3.3) years	42% female	Cross-sectional	~ 116	WBA Bone health	ActiGraph wGT3x-BT	ActiGraph wGT3x-BT	ActiGraph wGT3x-BT
Marshall 2022 [65]	2022	UK	With T1D: 11.9 (1.6) years Without T1D: 11.6 (2.2) years	43% female	Cross-sectional	With T1D: 20 Without T1D: 17	Cardiometabolic	GENEActiv	GENEActiv	GENEActiv
Matricciani 2021 [66]	2021	Australia	Children: 12.0 (0.4) years Parents: 44.0 (5.1) years	Children: 50% male Parents: 13% male	Cross-sectional	Children: 1,073 Adults: 1,378	WBA Cardiometabolic	GENEActiv	GENEActiv	GENEActiv
Mellow 2022 [67]	2022	Australia	65.5 (3) years	68% female	Cross-sectional	384	Cognition	Axivity AX3 Multimedia Activity Recall for Children and Adults (MARCA)	Axivity AX3 Multimedia Activity Recall for Children and Adults	Axivity AX3
Mellow 2024 [68]	2024	New Zealand	65.6 (3.0) years	67% female	Cross-sectional	378	Cognition Other	Axivity AX3	Axivity AX3	Axivity AX3
Migueles 2020 [69]	2020	Spain	10.0 (1.0) years	39.8% girls	Cross-sectional	93	Other	ActiGraph GT3X +	ActiGraph GT3X +	ActiGraph GT3X +
Migueles 2023 [70]	2023	Sweden	Baseline: 4.5 (0.1) years Follow up: 9.6 (0.1) years	Baseline: 4.5 (0.1) years Follow up: 9.6 (0.1) years	Longitudinal	201 children	WBA Fitness	ActiGraph GT3X +	ActiGraph GT3X +	ActiGraph GT3X +
Mitchell 2023 [71]	2023	United Kingdom	47 (0.6) years	52% female	Cross-sectional	4,481	Cognition	activPAL3 micro	activPAL3 micro	activPAL3 micro
Murphy 2024 [72]	2024	Australia	68.4 (9.2) years	92% male	Cross-sectional	25	HRQoL	GENEActiv	GENEActiv	GENEActiv
Ng 2021 [73]	2021	Australia	12.0 (0.4) years	49% female	Cross-sectional	1,874	WBA AAP HRQoL	GENEActiv	GENEActiv	GENEActiv
Niemelä 2024 [74]	2024	Finland	33.8 (0.6) years	60% female	Cross-sectional	1,134	WBA Cardiometabolic	Oura ring generation 2	Oura ring generation 2	Oura Ring Generation 2
Oviedo-Caro 2020 [75]	2020	Spain	32.8 (4.5) years	100% female	Cross-sectional	130	WBA Fitness	SenseWear Mini Armband	SenseWear Mini Armband	SenseWear Mini Armband
Powell 2020 [76]	2020	Ireland	64.6 (5.3) years	46% female	Cross-sectional	366	WBA Cardiometabolic	ActiviPAL3 Micro	ActiviPAL3 Micro	ActiviPAL3 Micro
Rasmussen 2023 [77]	2023	Czech Republic	13.9 (2.8) years	57.5% female	Cross-sectional	Children: 343 Adolescents: 316	WBA	ActiGraph wGT3X-BT ActiGraph GT9X Link	ActiGraph wGT3X-BT ActiGraph GT9X Link	ActiGraph wGT3X-BT ActiGraph GT9X Link
Rorem 2024 [78]	2024	Canada	3 to 5 years	Age 3: 48.1% girls Age 5: 49.6% girls	Cross-sectional Longitudinal	Age 3: 541 Age 5: 575	Depression Behavioral conduct	ActiGraphGT3X-BT Childhood Television Viewing Habits Questionnaire	ActiGraph GT3X-BT	ActiGraph GT3X-BT
Runacres 2023 [79]	2023	Wales	13.8 (1.8) years	47.7% female	Cross-sectional	Trained: 84 Untrained: 92	Cardiometabolic	ActiGraph GT3X	ActiGraph GT3X	ActiGraph GT3X
Sandborg 2022 [80]	2022	Sweden	31 (4) years	100% female + F67	Cross-sectional Longitudinal	273	WBA Cardiometabolic	ActiGraph wGT3x-BT	ActiGraph wGT3x-BT	ActiGraph wGT3x-BT
Smith 2021 [81]	2021	Iran and England	Iran: 9.8 (0.3) years England: 9.5 (0.6) years	Iran: 46% female England: 57% female	Cross-sectional	Iran: 119 England: 139	Motor skill(s)	GENEActiv	GENEActiv	GENEActiv
Smith 2021 [82]	2021	Australia	66.9 (4.5) years	44% female	Cross-sectional	34	Other	GENEActiv	GENEActiv	GENEActiv
Song 2018 [83]	2018	USA	67.1 (8.5) years	49.7% female	Cross-sectional	185	Other (pain)	ActiGraph GT3X	ActiGraph GT3X	ActiGraph GT3X
St Laurent 2023 [84]	2023	US	51.5 (9.5) months	44.4% female	Cross-sectional	388	Cognition Other	Actiwatch Spectrum	Actiwatch Spectrum	Actiwatch Spectrum
Swindell 2020 [85]	2020	Denmark Finland The Netherlands UK Spain Bulgaria Australia New Zealand	52.8 (11.1) years	64.6% female	Cross-sectional	1,462	WBA Cardiometabolic	ActiSleep +	ActiSleep +	ActiSleep +
Talarico 2018 [86]	2018	Canada	10 to 13 years	50.2% female	Cross-sectional	434	WBA	Actical	Actical	Actical
Tan 2023 [87]	2023	Singapore	8 and 10 years	50.5% female	Cross-sectional Longitudinal	370	HRQoL	ActiGraph GT3X + BT	ActiGraph GT3X + BT	ActiGraph GT3X + BT
Taylor 2018 [88]	2018	New Zealand	Mothers: 31.6 (5.2) years Children: 1 to 5 years	43.7 to 49.2% female	Cross-sectional Longitudinal	380	WBA Bone health	Actical	Actical	Actical
Taylor 2020 [89]	2020	New Zealand	7.9 (1.1) years	51.5% female	Cross-sectional	690	WBA	ActiGraph GT3X	ActiGraph GT3X	ActiGraph GT3X
Taylor 2023 [90]	2023	New Zealand	Baseline: 2 years	49.7% female	Cross-sectional Longitudinal	392	Depression Anxiety Cognition ER Behavioral conduct	Actical	Actical	Actical
Tyler 2022 [91]	2022	UK	11.5 (1.4) years	50.7% female	Cross-sectional	359	Motor skill(s)	ActiGraph GT9X	ActiGraph GT9X	ActiGraph GT9X
Vanderlinden 2023 [92]	2023	Belgium	71.3 (6.3) years	71% female	Cross-sectional	410	Well-being	ActiGraph wGT3X-BT	ActiGraph wGT3X-BT	ActiGraph wGT3X-BT
Verhoog 2020 [93]	2020	The Netherlands	70.9 (9.3) years	51.5% women	Cross-sectional	1,934	HRQoL	GENEActiv	GENEActiv	GENEActiv
Walmsley 2022 [94]	2022	UK	40 to 69 years	58% female	Longitudinal	87,498	Other	Axivity AX3	Axivity AX3	Axivity AX3

*ER *emotional regulation, *HRQoL * health-related quality of life, *PA *physical activity, *SB *sedentary behavior, *WBA *weight, body mass index, and/or adiposity

Physical health outcomes that were examined in the included articles included weight, BMI, or adiposity (*n* = 32), cardiometabolic markers (*n* = 16), fitness (*n* = 8), bone health (*n* = 5), motor skills (*n* = 3), injuries (*n* = 1), or other physical measures (*n* = 12). Mental, brain or cognitive health measures included cognition (*n* = 10), depression (*n* = 8), well-being or quality of life (*n* = 6), anxiety (*n* = 5), emotional regulation (*n* = 5), prosocial behavior (*n* = 5), behavioral conduct (*n* = 4), academic performance or achievement (*n* = 3), stress (*n* = 3), and school readiness (*n* = 1). The authors assessed physical activity and sedentary behavior using devices (*n* = 67) or a combination of devices and self-report (*n* = 3).

### Overall sleep reporting

Out of a total possible sum of 40, the combined author-derived reporting score, including all the above categories, was 20.3 ± 5.4 (range = 5 to 36) (Table 3).

**Table 3:** Combined summary of the presence of sleep reporting metrics and information

Study	Device Technical Settings								Sleep Period Recording							SC	Time-Use		Discussion		Total Score
	Placement	Epoch	Frequency	Manual/ Visual	Soft., Algo, &/or code	Sleep Log	Event Marker	Light	Wear Period	Valid Days	Types	Criteria	NW Defined	Sleep NW	NW Methods	Definitions	Cycle Defined	Closure	Limitations	Code	
Balbim 2024 [25]	Y	N	N	Y	Y	N	Y	Y	Y	Y	Y	Y	N	N	N	N	Y	Y	Y	Y	26
Bianchim 2022 [26]	Y	N	Y	Y	Y	N	N	N	Y	Y	N	U	U	U	N	Y	N	Y	N	N	21
Biddle 2018 [27]	Y	N	N	N	Y	N	N	N	Y	Y	N	Y	Y	Y	Y	Y	N	Y	Y	N	24
Blodgett 2023 [28]	Y	N	N	N	Y	N	N	N	Y	N	N	N	Y	U	N	Y	Y	N	Y	N	15
Blodgett 2024 [29]	Y	Y	N	N	Y	N	N	N	Y	Y	N	Y	Y	Y	N	N	N	Y	Y	N	22
Brakenridge 2021 [30]	Y	N	Y	N	N	Y	N	N	Y	N	Y	Y	N	N	N	N	N	Y	Y	N	18
Brakenridge 2024 [31]	Y	N	N	N	Y	N	N	N	Y	U	Y	Y	N	N	N	N	N	N	N	N	11
Brayton 2023 [32]	Y	N	N	N	Y	N	N	N	N	N	N	U	N	N	N	N	N	N	N	N	5
Cabanas-Sánchez 2021 [33]	Y	N	N	N	Y	N	N	N	Y	U	U	U	N	N	N	N	N	Y	N	N	13
Chen 2023 [34]	Y	Y	N	N	Y	N	N	N	Y	Y	Y	Y	N	N	N	N	N	Y	N	N	18
Chong 2021 [35]	Y	Y	Y	N	Y	N	N	N	Y	Y	Y	Y	Y	Y	Y	N	N	N	N	N	22
Collings 2023 [36]	Y	Y	Y	N	Y	N	N	N	Y	U	U	U	N	N	N	Y	N	Y	Y	N	21
Collings 2023 [37]	Y	Y	Y	N	Y	N	N	N	Y	U	U	U	N	N	U	Y	N	Y	Y	N	22
Curtis 2020 [38]	Y	Y	Y	Y	N	Y	N	N	Y	U	U	U	Y	Y	Y	N	U	Y	Y	N	28
Curtis 2023 [39]	Y	N	N	N	Y	N	N	N	Y	Y	Y	Y	Y	Y	Y	N	U	Y	U	N	24
deFaria 2022 [40]	Y	Y	Y	Y	Y	Y	N	N	Y	Y	Y	U	N	N	N	U	N	Y	N	N	24
Domingues 2022 [41]	Y	Y	Y	N	Y	N	N	N	Y	Y	Y	U	N	N	N	N	N	Y	Y	N	21
Dumuid 2017 [42]	Y	Y	Y	N	Y	N	N	N	Y	Y	N	Y	Y	U	Y	N	N	N	Y	N	20
Dumuid 2018 [43]	Y	Y	N	Y	Y	Y	N	N	Y	U	U	U	N	N	N	N	N	Y	Y	N	21
Dumuid 2018 [44]	Y	Y	Y	N	Y	N	N	N	Y	Y	Y	Y	N	N	N	N	N	Y	N	Y	20
Dumuid 2018 [45]	Y	Y	N	N	Y	N	N	N	Y	N	U	U	Y	N	Y	N	N	Y	N	Y	18
Dumuid 2019 [46]	Y	Y	Y	Y	N	Y	N	N	Y	Y	N	Y	Y	U	Y	N	N	Y	N	N	25
Dumuid 2020 [47]	Y	Y	Y	Y	Y	Y	N	N	Y	Y	N	Y	Y	U	U	N	N	Y	N	N	26
Dumuid 2021 [48]	Y	Y	Y	Y	N	Y	N	N	Y	Y	N	Y	N	U	U	N	N	Y	N	N	22
Dumuid 2022 [49]	Y	Y	Y	Y	Y	Y	N	N	Y	U	U	U	N	N	N	N	N	Y	N	N	21
Dumuid 2022 [50]	Y	Y	N	N	Y	Y	N	N	Y	Y	N	Y	N	N	N	N	N	Y	N	Y	18
Fairclough 2017 [51]	Y	Y	Y	N	Y	N	N	N	Y	U	U	U	Y	U	U	N	N	Y	Y	N	23
Fairclough 2021 [52]	Y	Y	Y	N	Y	N	N	N	Y	N	N	N	Y	U	U	N	N	Y	N	N	18
Fairclough 2023 [53]	Y	Y	Y	N	Y	N	N	N	Y	Y	Y	Y	U	U	U	N	N	Y	N	N	23
Gupta 2020 [54]	Y	N	N	Y	N	Y	N	N	Y	Y	U	Y	U	U	Y	Y	Y	Y	N	R	25
Healy 2021 [55]	Y	N	N	N	Y	N	N	N	Y	Y	N	N	Y	U	Y	Y	N	Y	N	N	19
Hofman 2022 [56]	Y	N	Y	N	Y	N	N	N	Y	U	U	U	Y	U	Y	N	N	Y	Y	N	22
Janda 2024 [57]	Y	Y	Y	N	Y	N	N	N	Y	U	U	U	U	U	Y	Y	Y	Y	N	R	25
Johansson 2020 [58]	Y	N	Y	Y	Y	Y	N	N	Y	U	U	U	Y	U	Y	Y	N	Y	Y	N	28
Kim 2021 [59]	U	Y	N	N	Y	N	N	N	Y	U	U	N	N	N	N	N	N	Y	N	N	13
Kuzik 2020 [60]	Y	Y	Y	Y	N	Y	N	N	Y	U	N	U	N	U	U	N	N	Y	N	N	20
Le 2022 [61]	Y	Y	N	N	Y	Y	N	N	Y	N	N	N	Y	Y	U	U	N	Y	Y	Y	22
Lee 2020 [62]	U	Y	N	N	Y	Y	N	N	Y	N	N	N	N	N	N	N	N	Y	Y	N	15
Lu 2024 [63]	Y	Y	Y	N	Y	N	N	N	Y	U	U	U	Y	U	U	N	N	N	N	N	17
Marmol-Perez 2024 [64]	Y	N	Y	N	Y	Y	N	N	Y	U	U	U	Y	U	U	N	N	N	N	N	17
Marshall 2022 [65]	Y	Y	Y	N	Y	N	N	N	Y	U	U	U	N	N	U	N	N	N	N	N	14
Matricciani 2021 [66]	Y	Y	N	Y	Y	Y	N	N	Y	Y	Y	Y	N	U	Y	Y	N	Y	Y	N	29
Mellow 2022 [67]	Y	N	Y	Y	Y	Y	N	N	Y	Y	Y	U	Y	Y	Y	N	N	Y	Y	N	29
Mellow 2024 [68]	Y	N	Y	Y	N	Y	N	N	Y	U	U	U	N	N	U	N	N	Y	Y	N	20
Migueles 2020 [69]	Y	U	Y	N	Y	Y	N	N	Y	N	U	U	N	N	U	N	N	N	N	N	14
Migueles 2023 [70]	Y	Y	Y	N	Y	N	N	N	Y	U	N	U	U	U	U	N	N	Y	Y	N	21
Mitchell 2023 [71]	Y	N	N	N	N	N	N	N	Y	U	N	U	N	N	N	Y	N	N	Y	N	10
Murphy 2024 [72]	Y	Y	Y	Y	Y	Y	N	N	Y	Y	Y	Y	Y	Y	Y	Y	N	Y	Y	N	34
Ng 2021 [73]	Y	Y	Y	N	Y	Y	N	N	Y	Y	Y	Y	N	N	Y	N	N	Y	N	N	24
Niemelä 2024 [74]	Y	Y	Y	N	Y	N	N	N	Y	Y	N	Y	N	N	Y	Y	N	Y	Y	N	24
Oviedo-Caro 2020 [75]	N	N	N	N	N	N	N	N	Y	Y	Y	Y	N	N	Y	Y	N	N	N	N	12
Powell 2020 [76]	Y	Y	Y	Y	N	N	N	N	Y	U	U	U	Y	U	U	N	N	Y	Y	N	23
Rasmussen 2023 [77]	Y	N	Y	N	Y	Y	N	N	Y	U	U	U	N	N	U	Y	N	Y	N	N	20
Rorem 2024 [78]	Y	Y	Y	Y	Y	Y	N	N	Y	U	N	U	Y	U	U	N	N	Y	N	N	24
Runacres 2023 [79]	Y	N	Y	N	Y	Y	N	N	Y	U	U	U	N	N	U	N	N	N	N	N	14
Sandborg 2022 [80]	Y	Y	Y	N	Y	Y	N	N	Y	U	N	U	N	N	U	N	N	N	N	N	15
Smith 2021 [81]	Y	Y	Y	Y	Y	Y	N	N	Y	U	U	U	N	N	U	N	N	N	N	N	18
Smith 2021 [82]	Y	Y	N	N	Y	N	N	N	Y	U	U	N	Y	U	U	N	N	N	N	N	14
Song 2018 [83]	Y	Y	N	N	Y	Y	N	Y	Y	Y	N	Y	Y	N	U	Y	N	N	N	N	21
St Laurent 2023 [84]	Y	Y	Y	Y	Y	Y	Y	N	Y	Y	N	Y	Y	Y	Y	Y	Y	Y	Y	N	36
Swindell 2020 [85]	Y	Y	N	N	Y	N	N	N	Y	U	U	U	Y	U	Y	N	N	Y	N	N	20
Talarico 2018 [86]	Y	Y	N	Y	N	N	N	N	Y	U	N	U	Y	U	U	N	N	Y	N	N	18
Tan 2023 [87]	Y	N	Y	N	Y	N	N	N	Y	U	U	U	Y	U	U	Y	N	N	Y	N	19
Taylor 2018 [88]	Y	Y	N	N	Y	N	N	N	Y	U	U	Y	Y	U	U	N	N	Y	Y	N	22
Taylor 2020 [89]	Y	Y	N	N	Y	N	N	N	Y	U	U	Y	Y	U	U	Y	Y	Y	N	N	24
Taylor 2023 [90]	Y	Y	N	N	Y	N	N	N	Y	U	N	Y	Y	U	U	Y	Y	Y	N	N	23
Tyler 2022 [91]	Y	Y	Y	N	Y	N	N	N	Y	U	Y	U	N	N	U	N	N	Y	N	N	19
Vanderlinden 2023 [92]	Y	N	N	N	Y	N	N	N	Y	U	N	U	N	N	U	N	N	Y	N	N	13
Verhoog 2020 [93]	Y	N	Y	N	Y	N	N	N	Y	N	N	N	Y	U	U	N	N	Y	Y	N	18
Walmsley 2022 [94]	Y	N	N	N	N	Y	N	N	Y	U	N	Y	N	Y	U	U	N	N	Y	N	14

*Algo* = algorithm, *NW* non-wear, *SC* sleep conceptualization, *Soft* software, *Y* yes, *N* no, *U* unclear, *R* upon request

### Device technical setting

A summary of the technical specifications for sleep-measurement devices and settings used in the included articles is presented in Table 3 and Additional File 4. Nineteen devices were utilized across the articles, with GENEActiv (*n* = 16) and ActiGraph GT3X + (*n* = 12) monitors being the most commonly used. Research-grade devices were most commonly used, with only two articles (2.9%) reporting metrics measured by consumer devices (i.e., Fitbit and Oura Ring) [38, 93]. All but three articles fully reported the device placement (*n* = 67; 95.7%). Concerning configuration parameters, 64.3% (*n* = 45) reported the epoch or interval settings, and 58.6% reported the sampling rates (*n* = 41). Reported scoring and processing information included manual scoring and/or visual inspection (30.0%, *n* = 21), use of software, algorithm(s), and/or code (82.9%, *n* = 58), a participant-reported sleep or daily log (41.4%, *n* = 29), use of a device event marker (2.9%, *n* = 2), and light measurements (2.9%, *n* = 2). Most articles used multiple inputs of information to score and process sleep metrics (*n* = 32; 45.7%), and two articles did not report this information (2.9%). Among these categories, the author-derived total reporting score for technical specifications was 7.6 ± 2.6 (range = 0 to 14, out of a possible maximum score of 16).

### Sleep period recording

Reported metrics relating to how sleep was recorded are presented in Table 3 and Additional File 5. Most articles indicated the planned or requested measurement period (98.6%, *n* = 69), which ranged from 2 to 28 days, nights, or 24-h periods. The minimum number of required valid days to be included in analyses was reported in 62 articles and ranged from 1 to 7 days, with 4 days or nights as the most frequent (*n* = 33). However, among the articles that reported minimum valid days, only 26 articles (37.0%) were clear that sleep periods were included in the criteria. Relatedly, although approximately 62% of studies (*n* = 43) described the type of days included (e.g., any days or specific requirements for weekday and weekend days or nights), only 22.9% were specific about sleep inclusion (e.g., the valid day criteria included a minimum duration of sleep data). Defining the criteria of a valid day was included for 89% of the articles (*n* = 62), with 38.6% (*n* = 27) including sleep specifications.

Similarly, how non-wear time was determined was identified clearly in 45.7% (*n* = 32) of all articles, but only 12.9% (*n* = 9) included clear reporting with respect to sleep periods. Approximately 28% of articles (*n* = 20) denoted how non-wear time that included sleep periods was addressed (e.g., excluded or imputed), whereas 44.3% (*n* = 31) described non-wear time decisions for waking periods only or did not distinctly specify this information. The author-derived overall average score when each item in the category was summed was 8.0 ± 2.7 (range = 1 to 14, with a maximum possible score of 14).

### Sleep conceptualization

Information about the sleep metrics in the included articles is highlighted in Table 3 and Additional File 6. Most articles conceptualized sleep as 24-h sleep (or all sleep within a daily cycle) (*n* = 33; 47.1%). Remaining articles referred to night sleep (*n* = 26), the major sleep period (*n* = 1), or did not specify (*n* = 10). Only 28.6% (*n* = 20) of articles included author definitions of the sleep metrics, with 4.3% (*n* = 3) reporting partial information. Most articles did not define their sleep metric (*n* = 47), and how the sleep or measurement cycle was treated (e.g., midnight-to-midnight or wake-to-wake) was only explicitly identified in 10% of the articles (*n* = 7). Only three (4.3% articles) specifically noted that daytime or nap sleep was included, but several articles (*n* = 7, 10%) implied that multiple sleep bouts would be eligible to be calculated within the sleep metrics. The author-derived total score in this category of reporting was 2.3 ± 1.6 (range = 0 to 6, with a maximum possible score of 6).

### Sleep integration in time-use compositions

Information about how sleep was addressed within a time-use composition is included in Table 3 and Additional File 6. How the composition was closed was described in 74.3% (*n* = 52) of the articles, with most of these studies (71.4%, *n* = 50) using 24 h (or 1,440 min). Although the number of compositional components ranged from 3 to 9, most articles (*n* = 67) only included one sleep component. Three articles included two sleep components in their compositions (i.e., sleep duration and wake after sleep onset). Compositional closure was the only scored component in this category and had an author-derived mean score of 1.5 ± 0.9 (range = 0 to 2, with a maximum possible score of 2), with 52 articles including this information (74.2%).

### Discussion and transparency

Factors relating to how sleep was discussed in the articles are also presented in Table 3 and Additional File 6. Forty-one percent of articles (*n* = 29) included a focus on limitations related to sleep measurement within their discussions. Therefore, the author-derived total sleep discussion score of this single item was 0.8 ± 0.9 (range = 0 to 2, with a maximum possible score of 2). Only five articles provided analysis code (6.9%), with two (2.7%) noting the code was available upon request.

### Methodological quality and risk of bias

With numeric total scores ranging from 5 to 11 (out of a possible maximum score of 14), the average study quality and risk score of the included articles was 7.3 ± 1.2 (Table 4). Most articles were classified as “fair” (*n* = 60, 85.7%), with the remaining 10 articles classified as “good” (14.3%) in study quality.

**Table 4:** Summary of the quality and bias question items and scores

	Did authors describe the goal in conducting this research?	Was the study population clearly specified and defined?	Was the participation rate of eligible persons at least 50%?	Were all the subjects selected or recruited from the same or similar populations? Were inclusion and exclusion criteria for being in the study prespecified and applied uniformly to all participants?	Was a sample size justification, power description, or variance and effect estimates provided?	For the analyses in this paper, were the exposure(s) of interest measured prior to the outcome(s) being measured?	Was the timeframe sufficient so that one could reasonably expect to see an association between exposure and outcome if it existed?	For exposures that can vary in amount or level, did the study examine different levels of the exposure as related to the outcome?	Were the exposure measures clearly defined, valid, reliable, and implemented consistently across all study participants?	Was the exposure(s) assessed more than once over time?	Were the outcome measures clearly defined, valid, reliable, and implemented consistently across all study participants?	Were the outcome assessors blinded to the exposure status of participants?	Was loss to follow-up after baseline 20% or less?	Were key potential confounding variables measured and adjusted statistically for their impact on the relationship between exposure(s) and outcome(s)?	**TOTAL**
Balbim 2024 [25]	Yes	Yes	No	No	No	No	No	Yes	Yes	No	Yes	No	No	Yes	6
Bianchim 2022 [26]	Yes	Yes	No	Yes	No	No	No	Yes	No	No	Yes	No	No	Yes	7
Biddle 2018 [27]	Yes	Yes	No	Yes	No	No	No	Yes	No	No	Yes	No	No	Yes	7
Blodgett 2023 [28]	Yes	Yes	Yes	Yes	No	No	No	Yes	No	No	Yes	No	No	Yes	8
Blodgett 2024 [29]	Yes	Yes	No	Yes	Yes	No	No	Yes	No	No	Yes	No	No	Yes	7
Brakenridge 2021 [30]	Yes	Yes	Yes	Yes	No	No	No	Yes	No	No	Yes	No	No	Yes	8
Brakenridge 2024 [31]	Yes	Yes	No	Yes	No	No	No	Yes	No	No	Yes	No	No	Yes	7
Brayton 2023 [32]	Yes	Yes	No	Yes	Yes	Yes	Yes	Yes	No	Oth	Yes	No	Oth	Yes	10
Cabanas-Sánchez 2021 [33]	Yes	Yes	Yes	Yes	No	Yes	Yes	Yes	No	No	Yes	No	No	Yes	10
Chen 2023 [34]	Yes	Yes	Yes	Yes	No	No	No	Yes	No	No	Yes	No	No	Yes	8
Chong 2021 [35]	Yes	Yes	No	Yes	No	Yes	Yes	Yes	Yes	No	Yes	No	No	Yes	9
Collings 2023 [36]	Yes	Yes	No	Yes	No	No	No	Yes	No	No	Yes	No	No	Yes	7
Collings 2023 [37]	Yes	Yes	Yes	Yes	No	No	No	Yes	No	No	Yes	No	No	Yes	8
Curtis 2020 [38]	Yes	Yes	No	Yes	No	No	No	Yes	Yes	No	Yes	No	No	Yes	7
Curtis 2023 [39]	Yes	Yes	No	Yes	No	No	No	Yes	No	No	Yes	No	No	Yes	7
deFaria 2022 [40]	Yes	Yes	No	No	Yes	No	No	Yes	Yes	No	Yes	No	No	Yes	7
Domingues 2022 [41]	Yes	Yes	No	Yes	Yes	No	No	Yes	No	No	Yes	No	No	Yes	8
Dumuid 2017 [42]	Yes	Yes	Yes	Yes	No	No	No	Yes	No	No	Yes	No	No	Yes	8
Dumuid 2018 [43]	Yes	Yes	Yes	Yes	Yes	No	No	Yes	No	No	Yes	No	No	Yes	9
Dumuid 2018 [44]	Yes	Yes	No	Yes	No	No	No	Yes	No	No	Yes	No	No	Yes	7
Dumuid 2018 [45]	Yes	No	No	No	Yes	No	No	Yes	No	No	Yes	No	No	Yes	6
Dumuid 2019 [46]	Yes	Yes	No	Yes	No	No	No	Yes	Yes	No	Yes	No	No	Yes	7
Dumuid 2020 [47]	Yes	Yes	Yes	Yes	No	No	No	Yes	Yes	No	Yes	No	No	Yes	8
Dumuid 2021 [48]	Yes	Yes	No	Yes	No	No	No	Yes	Yes	No	Yes	No	No	Yes	7
Dumuid 2022 [49]	Yes	Yes	No	Yes	No	No	No	Yes	No	No	Yes	No	No	Yes	6
Dumuid 2022 [50]	Yes	Yes	No	Yes	No	No	No	Yes	No	No	Yes	No	No	Yes	7
Fairclough 2017 [51]	Yes	Yes	Yes	Yes	No	No	No	Yes	No	No	Yes	No	No	Yes	8
Fairclough 2021 [52]	Yes	Yes	Yes	Yes	No	No	No	Yes	No	No	Yes	No	No	Yes	8
Fairclough 2023 [53]	Yes	Yes	No	No	No	No	No	Yes	No	No	Yes	No	No	Yes	6
Gupta 2020 [54]	Yes	Yes	Yes	Yes	No	No	No	Yes	Yes	No	Yes	No	Oth	Yes	8
Healy 2021 [55]	Yes	Yes	No	Yes	No	No	No	Yes	No	No	Yes	No	No	Yes	7
Hofman 2022 [56]	Yes	Yes	Yes	Oth	No	No	No	Yes	No	No	Yes	No	No	Yes	7
Janda 2024 [57]	Yes	Yes	No	Yes	No	No	No	Yes	Yes	No	Yes	No	No	Yes	7
Johansson 2020 [58]	Yes	Yes	No	Yes	No	No	No	Yes	Yes	No	Yes	No	No	Yes	7
Kim 2021 [59]	Yes	Yes	No	Yes	No	No	No	Yes	No	No	No	No	No	Yes	6
Kuzik 2020 [60]	Yes	Yes	No	Yes	No	No	No	Yes	No	No	Yes	No	No	Yes	7
Le 2022 [61]	Yes	Yes	No	Yes	No	No	No	Yes	No	No	Yes	No	No	Yes	7
Lee 2020 [62]	Yes	No	No	No	No	No	No	Yes	No	No	Yes	No	No	Yes	5
Lu 2024 [63]	Yes	Yes	Yes	Yes	No	No	No	Yes	No	No	Yes	No	No	Yes	8
Marmol-Perez 2024 [64]	Yes	Yes	No	No	No	No	No	Yes	No	No	Yes	No	No	Yes	5
Marshall 2022 [65]	Yes	Yes	No	Yes	No	No	No	Yes	No	No	Yes	No	No	Yes	7
Matricciani 2021 [66]	Yes	Yes	Yes	Yes	No	No	No	Yes	No	No	Yes	No	No	Yes	7
Mellow 2022 [67]	Yes	Yes	No	Yes	Yes	No	No	Yes	Yes	No	Yes	No	No	Yes	8
Mellow 2024 [68]	Yes	Yes	No	Yes	No	No	No	Yes	No	No	Yes	No	No	Yes	7
Migueles 2020 [69]	Yes	Yes	No	Yes	No	No	No	Yes	No	No	Yes	No	Yes	Yes	8
Migueles 2023 [70]	Yes	Yes	No	Yes	No	Yes	Yes	Yes	No	Yes	Yes	No	No	Yes	10
Mitchell 2023 [71]	Yes	Yes	Yes	Yes	No	No	No	No	No	No	No	Yes	No	Yes	6
Murphy 2024 [72]	Yes	Yes	No	Yes	Yes	No	No	Yes	Yes	No	Yes	No	No	Yes	8
Ng 2021 [73]	Yes	Yes	Yes	Yes	No	No	No	Yes	No	No	Yes	No	No	Yes	7
Niemelä 2024 [74]	Yes	Yes	No	No	No	No	No	Yes	No	No	Yes	No	No	Yes	6
Oviedo-Caro 2020 [75]	Yes	Yes	No	Yes	No	No	No	Yes	No	No	Yes	No	No	Yes	7
Powell 2020 [76]	Yes	Yes	No	Yes	No	No	No	Yes	No	No	No	No	No	Yes	6
Rasmussen 2023 [77]	Yes	Yes	No	Yes	No	No	No	Yes	No	No	Yes	No	No	Yes	7
Rorem 2024 [78]	Yes	Yes	Yes	Yes	No	Yes	Yes	Yes	Yes	Yes	Yes	No	No	Yes	11
Runacres 2023 [79]	Yes	No	No	Yes	No	No	No	Yes	No	No	Yes	No	No	Yes	6
Sandborg 2022 [80]	Yes	Yes	No	Yes	No	Yes	Yes	Yes	No	Yes	Yes	No	No	Yes	10
Smith 2021 [81]	Yes	Yes	Yes	Yes	No	No	No	No	No	No	No	No	No	Yes	5
Smith 2021 [82]	Yes	No	No	No	No	No	No	Yes	No	No	Yes	No	No	Yes	5
Song 2018 [83]	Yes	Yes	Yes	Yes	No	No	No	Yes	No	No	Yes	No	No	Yes	8
St Laurent 2023 [84]	Yes	Yes	No	Yes	No	No	No	Yes	Yes	No	Yes	No	No	Yes	7
Swindell 2020 [85]	Yes	Yes	Yes	Yes	No	No	No	Yes	No	No	Yes	No	No	Yes	8
Talarico 2018 [86]	Yes	Yes	No	No	No	No	No	Yes	No	No	Yes	No	No	Yes	5
Tan 2023 [87]	Yes	Yes	No	Yes	No	Yes	Yes	Yes	No	Yes	Yes	No	No	Yes	10
Taylor 2018 [88]	Yes	Yes	Yes	Yes	No	Yes	Yes	Yes	No	Yes	Yes	No	No	Yes	11
Taylor 2020 [89]	Yes	Yes	No	Yes	No	No	No	Yes	Yes	No	Yes	No	No	Yes	7
Taylor 2023 [90]	Yes	Yes	Yes	Yes	No	Yes	Yes	Yes	No	Yes	Yes	No	No	Yes	11
Tyler 2022 [91]	Yes	No	Yes	Yes	No	No	No	Yes	No	No	Yes	No	No	Yes	7
Vanderlinden 2023 [92]	Yes	Yes	No	No	No	No	No	Yes	No	No	No	No	No	Yes	5
Verhoog 2020 [93]	Yes	Yes	Yes	No	No	No	No	Yes	No	No	Yes	No	No	Yes	7
Walmsley 2022 [94]	Yes	Yes	No	Yes	No	Yes	No	Yes	No	No	Yes	No	No	Yes	7

*Oth* Other (i.e., ‘cannot determine,’ ‘not applicable,’ or ‘not reported)

## Discussion

This systematic review describes the current sleep reporting landscape of how device-assessed sleep has been measured, processed, and reported in observational studies using CoDA to examine associations between movement behaviors and health. Among the included articles, authors reported at least some information in each of the AASM and SBSM actigraphy reporting categories. Specifically, most articles included measurement period definitions, device placement for sleep assessment, how sleep was conceptualized, how the time-use composition was closed, and included sleep measurement-related limitations within the discussion. However, we identified several areas where reporting practices were lacking. Notably, few authors reported specifications for how they classified and captured sleep and how non-wear was defined or handled during sleep periods. In general, most articles described studies of fair or good quality. This review highlights areas of improvement for researchers to enhance CoDA reporting and understanding of sleep within associations between movement behaviors and health outcomes.

Several strengths in reporting practices were consistently observed among the included articles. Most reports demonstrated strong methodological transparency by clearly specifying the technical details of the devices used, including the device type and placement, which is critical for reproducibility and interpretation. In terms of recording procedures, articles generally defined what constituted a valid day of data and clearly outlined the planned measurement period, contributing to the consistency and reliability of data collection. Regarding the conceptualization of sleep, most articles explicitly described how sleep was defined within their analyses (i.e., whether as the primary or “major” sleep period, nighttime sleep, or over a full 24-h cycle), providing clarity on the analytical framework. Additionally, most articles reported how the compositional data were ‘closed,’ ensuring the compositional nature of the data was appropriately handled in the analysis.

Despite these strengths, there remain notable areas where reporting practices could be improved. In terms of technical specifications, there was substantial variability in the level of detail provided about device configuration settings and sleep scoring procedures. Decisions related to data collection (e.g., the selection of sleep detection algorithms or thresholds for sleep–wake classification) were often not described, despite their known impact on sleep estimates [15, 18]. Additionally, although time-use epidemiology focuses primarily on duration-based sleep estimates, many modern devices incorporate multiple biosensors (e.g., heart rate, skin temperature, electrodermal conductance, or blood oxygenation), which generate additional sleep-related indicators [14]. The inclusion of these diverse sensor inputs and the range in algorithms used further complicates cross-study consistency. These differences are particularly pronounced in consumer-grade wearables, where algorithmic transparency is limited, making it difficult to assess how sensor combinations influence behavioral classification. Without clear reporting of both algorithmic features and sensor combinations, reproducibility and comparability across studies are further compromised.

In the domain of data recording, most articles reported criteria for valid days and distinguished between weekdays and weekends. However, these criteria were often generalized across all 24-h movement behaviors, with limited attention to how a valid night of sleep was defined. This represents a missed opportunity, as transparent reporting of sleep-specific validity criteria (e.g., how sleep periods were defined, how non-wear during the night was handled, or how nights with irregular patterns were classified) plays an important role in interpreting sleep estimates [19]. Among included studies, non-wear detection was inconsistently addressed, and often focused only on waking periods, potentially conflating sleep with non-wear time. This is problematic as evidence shows that how non-wear time is reallocated or removed from compositional analyses can drastically alter the magnitude of association with health outcomes [95].

Further, across included studies, the definition of weekdays and weekends operated differently with respect to sleep. While Saturdays and Sundays are typically considered weekends for waking behaviors, a weekend night for sleep purposes includes Friday and Saturday. When constructing an’average’ composition for a weekday and weekend, it is therefore critical to specify how these periods are defined, as applying waking-behavior conventions to sleep data may wash out meaningful weekday-weekend differences. In the context of 24-h movement analyses, it may be warranted to align weekday-weekend definitions across sleep and activity behaviors to maintain internal consistency. However, the most appropriate approach will depend on a study’s objectives, underscoring the need for transparent reporting of these decisions.

Regarding sleep conceptualization, while many articles specified whether the analysis included nighttime or 24-h sleep, several did not define the specific metrics used (e.g., total sleep time, time in bed, or wake after sleep onset) even though these variables represent distinct physiological constructs and behavioral patterns [96]. Additionally, few articles clarified whether daytime sleep or multiple sleep bouts (e.g., naps or segmented sleep) were considered, despite their relevance to total sleep time [97] and they have a higher prevalence among younger and older populations.

With respect to composition construction, most articles measured more than one sleep-related variable, yet many included only a single sleep metric in the final time-use composition. This practice may oversimplify the complexity of sleep behavior and ignore important dimensions of sleep health that could influence outcomes [96]. Furthermore, fewer than half of the reviewed articles discussed limitations related to sleep measurement or the treatment of sleep in the CoDA framework. Transparent acknowledgment of such limitations is crucial, as methodological choices (e.g., how sleep is defined and incorporated into compositional models) can substantially affect interpretations of time-use trade-offs [98].

Several recurring issues emerged that warrant attention in future work. One notable concern was the inconsistency in how sleep metrics were defined and reported. While we tracked whether authors included definitions for sleep metrics (e.g., sleep duration or time in bed), these definitions often varied across articles and were not always aligned with established standards such as those from the AASM or SBSM [15, 97]. This lack of standardization can introduce confusion and limit comparability across studies. For example, a substantial number of studies used time in bed as a proxy for sleep duration. While time in bed is commonly estimated with 24-h wearable device data, it may be a poor substitute for actual sleep duration in that it overestimates total sleep and provides no information about wake time in bed [19, 96]. Such substitutions, if not clearly defined and justified, risk misrepresenting the role of sleep within time-use compositions.

This systematic review has some limitations that should be considered when interpreting the findings. The quantitative tool we used in this review served as a method to capture reporting consistency across reports, not as a prescriptive checklist. Indeed, many of the elements we evaluated, such as configuration parameters (e.g., sampling rate, epoch length), may not be relevant or necessary for all devices or research designs. As such, we caution against interpreting the absence of specific reporting elements as a flaw, recognizing that flexibility is necessary to accommodate the diversity of sleep measurement tools and methodologies currently in use. Additionally, our focus on observational studies that treated time-use compositions as exposures and health outcomes as dependent variables may limit the generalizability of findings, as we did not include all CoDA studies involving device-measured sleep (e.g., studies specifying the time-use composition as the dependent variable). However, because our focus was on reporting practices rather than methodological quality or outcomes, we believe our recommendations remain relevant across a range of study designs and modeling approaches. Although we concentrated on CoDA due to its increasing use in time-use research, many of the reporting considerations (e.g., defining sleep metrics clearly and detailing measurement methods) are likely applicable to other analytical approaches examining 24-h behavior patterns and health outcomes.

## Conclusions

This systematic review identified several areas for improvement in how sleep metrics are reported within CoDA studies. A particularly noteworthy gap was the lack of detail in how sleep was classified and measured, with few studies specifying definitions or aligning with established reporting guidelines. To enhance clarity and comparability, researchers should aim to follow standardized reporting practices, such as those outlined by recognized sleep research organizations, and provide sleep measurement details with the same rigor applied to waking behaviors. Consistent use of sleep metrics, along with greater granularity in reporting sleep components (e.g., onset, offset, wake after sleep onset), can improve the interpretability of the importance of sleep as a component of the 24-h cycle for health. Where possible, all sleep periods, including daytime naps, should be measured and reported (even in populations where napping is infrequent) as these behaviors contribute meaningfully to the daily movement behavior cycle, particularly in children and older adults. Moving forward, increased awareness of sleep measurement limitations and transparent reporting practices will be essential for advancing the quality, transparency, and interpretability of CoDA-based sleep and health research.

## Supplementary Information

Additional file 1: PRISMA checklist.

Additional file 2: Database search strategy.

Additional file 3: Excluded articles from the full-text screening phase.

Additional file 4: Summary of device technical settings.

Additional file 5: Summary of sleep period specifications.

Additional file 6: Summary of sleep conceptualization, integration in time-use compositions, and discussion.

Additional file 7: Funding and conflict of interest disclosures of included studies.

## Data Availability

The datasets used and/or analyzed during the current study are available from the corresponding author on reasonable request.

## Abbreviation

CoDA: Compositional data analysis
