# Supplementary material for: Characterizing device-measured sleep in observational health research using compositional data analysis: a systematic review
Source: Int J Behav Nutr Phys Act. 2026 Jun 15;23:78. doi: 10.1186/s12966-026-01935-8 (PMC13501660; doi:10.1186/s12966-026-01935-8)
Supplement: Supplementary file 2 [file 12966_2026_1935_MOESM2_ESM.pdf]

Additional File 2. Database search strategy.

PUBMED

("Screen Time"[Mesh] OR "Sedentary Behavior"[Mesh] OR "Sitting Position"[Mesh] OR Inactiv*[Title/Abstract] OR Stationar*[Title/Abstract]) AND ("Sleep"[Mesh] OR "Circadian Clocks"[Mesh] OR "Circadian Rhythm"[Mesh] OR "Rest"[Mesh] OR "Bed time" OR "Wake time" OR Nocturnal OR Diurnal OR Offset OR "Rise time" OR Nighttime) AND ("exercise"[MeSH Terms] OR Physical*[Title/Abstract] OR "Resistance Training"[Mesh] OR "Physical Education and Training"[Mesh] OR "Endurance Training"[Mesh] OR Light*[Title/Abstract] OR MVPA[Title/Abstract] OR moderate*[Title/Abstract] OR vigorous*[Title/Abstract] OR aerobic[Title/Abstract] OR strength*[Title/Abstract] OR fitness[Title/Abstract] OR activity[Title/Abstract]) AND (composition*[Title/Abstract] OR isotemporal[Title/Abstract] OR log-ratio[Title/Abstract] OR ILR[Title/Abstract] OR CoDA[Title/Abstract] OR Reallocate*[Title/Abstract] OR “log ratio”[Title/Abstract] OR movement behave*[Title/Abstract] OR 24-hour movement behave*[Title/Abstract] OR 24-hour activit*[Title/Abstract] OR “Time use”[Title/Abstract] OR Time-use[Title/Abstract])

Limiters: Publication date: 2015-2024

Results: 212 articles

PSYCINFO

(TI (Sedentar* OR Inactiv* OR Sitt* OR “Screen time” OR Stationar*) OR AB (Sedentar* OR Inactiv* OR Sitt* OR “Screen time” OR Stationar*)) AND (TI (Sleep* OR Nap* OR Circadian* OR “Bed time” OR “Wake time” OR Nocturnal OR Diurnal OR Onset OR Offset OR “Rise time” OR Nighttime) OR AB (Sleep* OR Nap* OR Circadian* OR “Bed time” OR “Wake time” OR Nocturnal OR Diurnal OR Onset OR Offset OR “Rise time” OR Nighttime)) AND (TI (Exercise OR Sports OR Physical* OR Training OR Light* OR MVPA OR Moderate* OR Vigorous* OR Aerobic OR Strength* OR Fitness OR Activity) OR AB (Exercise OR Sports OR Physical* OR Training OR Light* OR MVPA OR Moderate* OR Vigorous* OR Aerobic OR Strength* OR Fitness OR Activity)) AND (TI (composition* OR isotemporal OR log-ratio OR ILR OR CoDA OR Reallocate* OR “log ratio” OR movement behave* OR 24-hour movement behave* OR 24-hour activit* OR “Time use” OR Time-use) OR AB (composition* OR isotemporal OR log-ratio OR ILR OR CoDA OR Reallocate* OR “log ratio” OR movement behave* OR 24-hour movement behave* OR 24-hour activit* OR “Time use” OR Time-use))

Limiters - Publication Year: 2015-2024; Narrow by Language: English

Results: 166

SPORTDISCUS

((TI (Sedentar* OR Inactiv* OR Sitt* OR “Screen time” OR Stationar*) OR AB (Sedentar* OR Inactiv* OR Sitt* OR “Screen time” OR Stationar*))) AND ((TI (Sleep* OR Nap* OR Circadian* OR “Bed time” OR “Wake time” OR Nocturnal OR Diurnal OR Onset OR Offset OR “Rise time” OR Nighttime) OR AB (Sleep* OR Nap* OR Circadian* OR “Bed time” OR “Wake time” OR Nocturnal OR Diurnal OR Onset OR Offset OR “Rise time” OR Nighttime))) AND ((TI (Exercise OR Sports OR Physical* OR Training OR Light* OR MVPA OR Moderate* OR Vigorous* OR Aerobic OR Strength* OR Fitness OR Activity) OR AB (Exercise OR Sports OR Physical* OR Training OR Light* OR MVPA OR Moderate* OR Vigorous* OR Aerobic OR Strength* OR Fitness OR Activity))) AND ((TI (composition* OR isotemporal OR log-ratio OR ILR OR CoDA OR Reallocate* OR “log ratio” OR movement behave* OR 24-hour movement behave* OR 24-hour activit* OR “Time use” OR Time-use) OR AB (composition* OR isotemporal OR log-ratio OR ILR OR CoDA OR Reallocate* OR “log ratio” OR movement behave* OR 24-hour movement behave* OR 24-hour activit* OR “Time use” OR Time-use)))

Limiters - Publication Date: 20150101-20240012; Language: English

Results: 116 articles

CINAHL

TI (Sedentar* OR Inactiv* OR Sitt* OR “Screen time” OR Stationar*) OR AB (Sedentar* OR Inactiv* OR Sitt* OR “Screen time” OR Stationar*) AND TI (Sleep* OR Nap* OR Circadian* OR “Bed time” OR “Wake time” OR Nocturnal OR Diurnal OR Onset OR Offset OR “Rise time” OR Nighttime) AND AB (Sleep* OR Nap* OR Circadian* OR “Bed time” OR “Wake time” OR Nocturnal OR Diurnal OR Onset OR Offset OR “Rise time” OR Nighttime) AND TI (Exercise OR Sports OR Physical* OR Training OR Light* OR MVPA OR Moderate* OR Vigorous* OR Aerobic OR Strength* OR Fitness OR Activity) AND AB (Exercise OR Sports OR Physical* OR Training OR Light* OR MVPA OR Moderate* OR Vigorous* OR Aerobic OR Strength* OR Fitness OR Activity) AND TI (composition* OR isotemporal OR log-ratio OR ILR OR CoDA OR Reallocate* OR “log ratio” OR movement behave* OR 24-hour movement behave* OR 24-hour activit* OR “Time use” OR Time-use) AND AB(composition* OR isotemporal OR log-ratio OR ILR OR CoDA OR Reallocate* OR “log ratio” OR movement behave* OR 24-hour movement behave* OR 24-hour activit* OR “Time use” OR Time-use)

Limiters - Publication Date: 20150101-20240912; English Language; Academic Journals

Results: 6,046 articles

WEB OF SCIENCE

(Sedentar* OR Inactiv* OR Sitt* OR “Screen time” OR Stationar*) AND (Sleep* OR Nap* OR Circadian* OR “Bed time” OR “Wake time” OR Nocturnal OR Diurnal OR Onset OR Offset OR “Rise time” OR Nighttime) AND (Exercise OR Sports OR Physical* OR Training OR Light* OR MVPA OR Moderate* OR Vigorous* OR Aerobic OR Strength* OR Fitness OR Activity) AND (composition* OR isotemporal OR log-ratio OR ILR OR CoDA OR Reallocate* OR “log ratio” OR movement behave* OR 24-hour movement behave* OR 24-hour activit* OR “Time use” OR Time-use**)** (Title) OR (Sedentar* OR Inactiv* OR Sitt* OR “Screen time” OR Stationar*) AND (Sleep* OR Nap* OR Circadian* OR “Bed time” OR “Wake time” OR Nocturnal OR Diurnal OR Onset OR Offset OR “Rise time” OR Nighttime) AND (Exercise OR Sports OR Physical* OR Training OR Light* OR MVPA OR Moderate* OR Vigorous* OR Aerobic OR Strength* OR Fitness OR Activity) AND (composition* OR isotemporal OR log-ratio OR ILR OR CoDA OR Reallocate* OR “log ratio” OR movement behave* OR 24-hour movement behave* OR 24-hour activit* OR “Time use” OR Time-use) (Abstract)

Refined by: Timespan: 2015-01-01 to 2024-07-12 (Publication Date); English (Languages)

Results: 796 articles

SCOPUS

TITLE-ABS-KEY ((sedentar* OR inactiv* OR sitt* OR "Screen time" OR stationar*) AND (sleep* OR nap* OR circadian* OR "Bed time" OR "Wake time" OR nocturnal OR diurnal OR onset OR offset OR "Rise time" OR nighttime) AND (exercise OR sports OR physical* OR training OR light* OR mvpa OR moderate* OR vigorous* OR aerobic OR strength* OR fitness OR activity) AND (composition* OR isotemporal OR log-ratio OR ilr OR coda OR reallocate* OR "log ratio" OR movement AND behave* OR 24-hour AND movement AND behave* OR 24-hour AND activit* OR "Time use" OR time-use)) AND PUBYEAR > 2014 AND (LIMIT-TO (LANGUAGE , "English"))

Results: 319 articles

COCHRANE

(Sedentar* OR Inactiv* OR Sitt* OR “Screen time” OR Stationar*) OR AB (Sedentar* OR Inactiv* OR Sitt* OR “Screen time” OR Stationar*):ti,ab,kw AND (Sleep* OR Nap* OR Circadian* OR “Bed time” OR “Wake time” OR Nocturnal OR Diurnal OR Onset OR Offset OR “Rise time” OR Nighttime):ti,ab,kw AND (Exercise OR Sports OR Physical* OR Training OR Light* OR MVPA OR Moderate* OR Vigorous* OR Aerobic OR Strength* OR Fitness OR Activity):ti,ab,kw AND (composition* OR isotemporal OR log-ratio OR ILR OR CoDA OR Reallocate* OR “log ratio” OR movement behave* OR “Time use” OR Time-use):ti,ab,kw

with Cochrane Library publication date from Jan 2015 to Jul 2024, in Trials, Clinical Answers, Editorials and Special Collections (Word variations have been searched)

Limiters: English Language

Results: 1,352 articles
