# Supplementary material for: Characterizing device-measured sleep in observational health research using compositional data analysis: a systematic review
Source: Int J Behav Nutr Phys Act. 2026 Jun 15;23:78. doi: 10.1186/s12966-026-01935-8 (PMC13501660; doi:10.1186/s12966-026-01935-8)
Supplement: Supplementary file 3 [file 12966_2026_1935_MOESM3_ESM.pdf]

Additional File 3. Excluded articles from the full-text screening phase.

First Author Last Name	Year	Title	Journal	Volume	Issue	Pages	DOI	First Exclusion Reason
Aadahl	2021	Should leisure-time sedentary behavior be replaced with sleep or physical activity for prevention of diabetes?	Scand J Med Sci Sports	31	5	1105-1114	10.1111/sms.13924	Analysis approach
Armstrong	2019	Featured Article: Bidirectional Effects of Sleep and Sedentary Behavior Among Toddlers: A Dynamic Multilevel Modeling Approach.	Journal of Pediatric Psychology	44	3	275-285	10.1093/jpepsy/jsy089	Analysis approach
Bahl	2021	Changes in 24-Hour Physical Activity Patterns and Walking Gait Biomechanics After Primary Total Hip Arthroplasty: A 2-Year Follow-up Study.	J Bone Joint Surg Am	103	13	1166-1174	10.2106/JBJS.20.01679	Analysis approach
Berninger	2020	Bidirectional Day-to-Day Associations of Reported Sleep Duration With Accelerometer Measured Physical Activity and Sedentary Time Among Dutch Adolescents: An Observational Study.	Journal for the Measurement of Physical Behaviour	3	4	304-314	10.1123/jmpb.2020-0010	measure method
Brown	2021	Adolescent movement behaviour profiles are associated with indicators of mental wellbeing	Mental Health and Physical Activity	20			10.1016/j.mhpa.2021.100387	measure method
Buman	2014	Reallocating time to sleep, sedentary behaviors, or active behaviors: Associations with cardiovascular disease risk biomarkers, NHANES 2005-2006	American Journal of Epidemiology	179	3	323-334	10.1093/aje/kwt292	Analysis approach
Butte	2016	Role of physical activity and sleep duration in growth and body composition of preschool-aged children.	Obesity	24	6	1328-35	10.1002/oby.21489	Analysis approach
Cabanas-Sanchez	2020	Twenty four-hour activity cycle in older adults using wrist-worn accelerometers: The seniors-ENRICA-2 study.	Scand J Med Sci Sports	30	4	700-708	10.1111/sms.13612	Outcome/no outcome

Cabanas-Sanchez	2019	24-h Movement and Nonmovement Behaviors in Older Adults. The IMPACT65+Study	Medicine & Science in Sports & Exercise	51	4	671-680	10.1249/MSS.0000000000001838	Analysis approach
Caetano	2024	Individual, family, school and neighborhood predictors related to different levels of physical activity in adolescents: A cross-sectional study	PLOS ONE	19	8		10.1371/journal.pone.0304737	Analysis approach
Cai	2021	Independent and combined associations between multiple lifestyle behaviours and academic grades of inner urban and peri-urban high school students: a cross-sectional study in Chongqing, China	BMJ Open	11	11		10.1136/bmjopen-2021-049508	Analysis approach
Callender	2021	Which intensities, types, and patterns of movement behaviors are most strongly associated with cardiometabolic risk factors among children?	Journal Of Sport and Health Science	10	3	368-378	10.1016/j.jshs.2019.06.006	Analysis approach
Carneiro-Barrera	2020	Body Composition Impact on Sleep in Young Adults: The Mediating Role of Sedentariness, Physical Activity, and Diet	Journal of Clinical Medicine	9	5		10.3390/jcm9051560	Analysis approach
Chaput	2018	Inequality in physical activity, sedentary behaviour, sleep duration and risk of obesity in children: a 12-country study	Obesity Science & Practice	4	3	229-237	10.1002/osp4.271	Analysis approach
Charman	2023	Physical activity, inactivity and sleep in older patients with coronary artery disease following percutaneous coronary intervention: a longitudinal, observational study.	Coronary Artery Disease	34	6	441-447	10.1097/MCA.0000000000001252	Analysis approach
Chastin	2021	Joint association between accelerometry-measured daily combination of time spent in physical activity, sedentary behaviour and sleep and all-cause mortality: a pooled analysis of six prospective cohorts using compositional analysis.	Br J Sports Med	55	22	1277-1285	10.1136/bjsports-2020-102345	Measure method

Chen	2020	Associations between early-life screen viewing and 24 hour movement behaviours: findings from a longitudinal birth cohort study.	Lancet Child Adolesc Health	4	3	201-209	10.1016/S2352-4642(19)30424-9	Analysis approach
Chen	2022	The Associations between 24-Hour Movement Behaviours and Quality of Life in Preschoolers: A Compositional Analysis of Cross-Sectional Data from 2018-2021.	Int J Environ Res Public Health	19	22		10.3390/ijerph192214969	Measure method
Chen	2024	The associations of chronic pain and 24-h movement behaviors with incident mental disorders: evidence from a large-scale cohort study	BMC Medicine	22	1		10.1186/s12916-024-03534-5	Measure method
Chen	2021	Meeting 24-h movement guidelines: Prevalence, correlates, and the relationships with overweight and obesity among Chinese children and adolescents	Journal of Sport and Health Science	10	3	349-359	https://doi.org/10.1016/j.jshs.2020.07.002	Analysis approach
Chong	2022	Changes in 24-Hour Domain-Specific Movement Behaviors and Their Associations with Children: Psychosocial Health During the Transition From Primary to Secondary School: A Compositional Data Analysis	Journal of Physical Activity and Health	19	5	358-366	10.1123/jpah.2021-0630	Measure method
Clarke	2021	Meeting Canadian 24-Hour Movement Guideline recommendations and risk of all-cause mortality	Applied Physiology Nutrition and Metabolism	46	12	1487-1494	10.1139/apnm-2021-0010	Analysis approach
Collings	2015	Prospective associations between sedentary time, sleep duration and adiposity in adolescents.	Sleep Medicine	16	6	717-722	10.1016/j.sleep.2015.02.532	Analysis approach
Corral-Perez	2024	Associations Between Physical Activity and Inactivity Levels on Physical Function and Sleep Parameters of Older Adults with Frailty Phenotype.	Journal of Applied Gerontology	43	7	910-921	10.1177/07334648231218095	Analysis approach
Costa	2022	Are objective measures of sleep and sedentary behaviours related to low back pain flares?	PAIN	163	9	1829-1837	10.1097/j.pain.0000000000002585	Analysis approach

D'Souza	2023	Differing associations with childhood outcomes using behavioural patterns derived from three data reduction techniques	International Journal of Epidemiology	52	2	577-588	10.1093/ije/dyac142	Analysis approach
de Paula	2024	Associations of activity, sedentary and sleep behaviors with prevalent steatotic liver disease in middle-aged and older adults: the ELSA-Brasil study	Journal of Activity, Sedentary and Sleep Behaviors	3	1	16-16	10.1186/s44167-024-00055-7	Measure method
Dietz-Terjung	2021	Association between habitual physical activity (HPA) and sleep quality in patients with cystic fibrosis	Sleep and Breathing	25	2	609-615	10.1007/s11325-020-02130-0	Analysis approach
Dong	2024	Impact of Replacing Sedentary Behavior with Physical Activity and Sleep on Stroke Risk: A Prospective Cohort Study	Nature and Science of Sleep	16		1611-1622	10.2147/NSS.S482276	Measure method
Drenowatz	2016	The Association of Physical Activity during Weekdays and Weekend with Body Composition in Young Adults	Journal of Obesity	2016			10.1155/2016/8236439	Analysis approach
Du	2024	Accelerometer-Measured Daily Behaviors That Mediate the Association Between Refractive Status and Depressive Disorders	Translational Vision Science & Technology	13	7		10.1167/tvst.13.7.3	Analysis approach
Duarte	2024	Associations of nighttime sleep, midday napping, and physical activity with all-cause mortality in older adults: the Seniors-ENRICA cohorts	Geroscience				10.1007/s11357-024-01351-5	Analysis approach
Dumuid	2018	Compositional data analysis for physical activity, sedentary time and sleep research.	Stat Methods Med Res	27	12	3726-3738	10.1177/0962280217710835	Study Design
Dumuid	2017	The compositional isotemporal substitution model: A method for estimating changes in a health outcome for reallocation of time between sleep, physical activity and sedentary behaviour	Statistical Methods in Medical Research	28	3	846-857	10.1177/0962280217737805	Study Design

Duncan	2024	"Goldilocks days" for adolescent mental health: Movement behaviour combinations for well-being, anxiety and depression by gender	Mental Health and Physical Activity	26			10.1016/j.mhpa.2023.100572	Measure method
Duvivier,	2017	Benefits of Substitution Sitting with Standing and Walking in Free-living Conditions for Cardiometabolic Risk Markers, cognition and Mood in Overweight Adults	Frontiers in Physiology	8			10.3389/fphys.2017.00353	Study Design
Ehlers	2018	Replacing sedentary time with physical activity or sleep: effects on cancer-related cognitive impairment in breast cancer survivors.	BMC Cancer	18	1	685	10.1186/s12885-018-4603-3	Analysis approach
Ezeugwu	2021	Influence of Neighborhood Characteristics and Weather on Movement Behaviors at Age 3 and 5 Years in a Longitudinal Birth Cohort	Journal of Physical Activity & Health	18	5	571-579	10.1123/jpah.2020-0827	Analysis approach
Ezeugwu	2017	Sleep Duration, Sedentary Behavior, Physical Activity, and Quality of Life after Inpatient Stroke Rehabilitation.	Journal of Stroke & Cerebrovascular Diseases	26	9	2004-2012	10.1016/j.jstrokecerebrovasdis.2017.06.009	Analysis approach
Fanning	2017	Replacing sedentary time with sleep, light, or moderate-to-vigorous physical activity: effects on self-regulation and executive functioning.	J Behav Med	40	2	332-342	10.1007/s10865-016-9788-9	Analysis approach
Farrahi	2023	AccNet24: A deep learning framework for classifying 24-hour activity behaviours from wrist-worn accelerometer data under free-living environments	International Journal of Medical Informatics	172			10.1016/j.ijmedinf.2023.105004	Analysis approach
Feehan	2020	Twenty-Four Hour Activity and Sleep Profiles for Adults Living with Arthritis: Habits Matter.	Arthritis Care Res (Hoboken)	72	12	1678-1686	10.1002/acr.24424	Analysis approach
Feehan	2024	Twenty-four hour physical activity, sedentary behaviour and sleep profiles in adults living with rheumatoid arthritis: a cross-sectional latent class analysis	Journal of Activity, Sedentary and Sleep Behaviors	3	1	10-Oct	10.1186/s44167-024-00049-5	Analysis approach
Ferguson	2023	Weather associations with physical activity, sedentary behaviour and sleep patterns of Australian adults: a longitudinal study with implications for climate change.	Int J Behav Nutr Phys Act	20	1	30	10.1186/s12966-023-01414-4	Analysis approach

Fernandez	2022	Temporal and bidirectional associations between physical activity and sleep in adolescents	International Journal of Sport Psychology	53	5	475-496	10.7352/IJSP.2022.53.475	Analysis approach
Feter	2023	Association Between 24-Hour Movement Behavior and Cognitive Function in Brazilian Middle-Aged and Older Adults: Findings From the ELSA-Brasil	Innovation in Aging	7	3		10.1093/geroni/igad030	Measure method
Full	2020	Modeling the cardiometabolic benefits of sleep in older women: exploring the 24-hour day.	Sleep	43	1		10.1093/sleep/zsz205	Analysis approach
Full	2019	Latent profile analysis of accelerometer-measured sleep, physical activity, and sedentary time and differences in health characteristics in adult women	PLOS ONE	14	6		10.1371/journal.pone.0218595	Analysis approach
Fung	2023	Adherence to 24-Hour Movement Recommendations and Health Indicators in Early Adolescence: Cross-Sectional and Longitudinal Associations in the Adolescent Brain Cognitive Development Study	Journal of Adolescent Health	72	3	460-470	10.1016/j.jadohealth.2022.10.019	Analysis approach
Galmes-Panades	2019	Isotemporal substitution of inactive time with physical activity and time in bed: cross-sectional associations with cardiometabolic health in the PREDIMED-Plus study.	Int J Behav Nutr Phys Act	16	1	137	10.1186/s12966-019-0892-4	Analysis approach
Gao	2024	Meeting 24-h movement behavior guidelines is linked to academic engagement, psychological functioning, and cognitive difficulties in youth with internalizing problems	Journal of Affective Disorders	349		176-186	10.1016/j.jad.2024.01.017	Analysis approach
Garaulet	2011	Short sleep duration is associated with increased obesity markers in European adolescents: Effect of physical activity and dietary habits. the HELENA study	International Journal of Obesity	35	10	1308-1317	10.1038/ijo.2011.149	Analysis approach

Garcia Hermoso	2022	Trajectories of 24-h movement guidelines from middle adolescence to adulthood on depression and suicidal ideation: a 22-year follow-up study	International Journal of Behavioral Nutrition and Physical Activity	19	1	135-135	10.1186/s12966-022-01367-0	Analysis approach
German	2021	Sleep, Sedentary Behavior, Physical Activity, and Cardiovascular Health: MESA.	Med Sci Sports Exerc	53	4	724-731	10.1249/MSS.0000000000002534	Analysis approach
Gil	2017	Seasonality of physical activity, sedentary behavior and nighttime sleep duration in middle aged and elderly population of the Rotterdam Study.	Maturitas	103		94-94	10.1016/j.maturitas.2017.06.018	Publication type
Gilson	2021	VO2peak and 24,Äêhour sleep, sedentary behavior, and physical activity in Australian truck drivers.	Scandinavian Journal of Medicine & Science in Sports	31	7	1574-1578	10.1111/sms.13965	Analysis approach
Giuntella	2021	Lifestyle and mental health disruptions during COVID-19	PNAS Proceedings of the National Academy of Sciences of the United States of America	118	9	9-Jan	10.1073/pnas.2016632118	Analysis approach
Grant	2023	Sleep Time, Physical Activity, and Screen Time among Montana American Indian Youth.	Int J Environ Res Public Health	20	17		10.3390/ijerph20176658	Analysis approach
Group: ORISCAV LUX	2022	Associations Between Wearable-Specific Indicators of Physical Activity Behaviour and Insulin Sensitivity and Glycated Haemoglobin in the General Population: Results from the ORISCAV-LUX 2 Study	Sports Medicine-Open	8	1		10.1186/s40798-022-00541-9	Analysis approach
Group: ORISCAV LUX	2024	Associations of movement behaviours and dietary intake with arterial stiffness: results from the ORISCAV-LUX 2 cross-sectional study	BMJ Open	14	7	10-Jan	10.1136/bmjopen-2024-084933	Analysis approach

Guan	2020	Proportion of kindergarten children meeting the WHO guidelines on physical activity, sedentary behaviour and sleep and associations with adiposity in urban Beijing.	BMC Pediatrics	20	1	9-Jan	10.1186/s12887-020-1969-6	Analysis approach
Guo	2022	Associations between Parents' Digital Media Habits, Engagement, Awareness, and Movement Guidelines among Preschool-Age Children: International Ipreschooler Surveillance Study	International Journal of Environmental Research and Public Health	19	17		10.3390/ijerph191710484	Analysis approach
Gupta	2018	Is Daily Composition of Movement Behaviors Related to Blood Pressure in Working Adults?	Medicine & Science in Sports & Exercise	50	10			Measure method
Gupta	2018	Is Daily Composition of Movement Behaviors Related to Blood Pressure in Working Adults?	Medicine and Science in Sports and Exercise	50	10	2150-2155	10.1249/MSS.0000000000001680	Measure method
Gupta	2016	What Is the Effect on Obesity Indicators from Replacing Prolonged Sedentary Time with Brief Sedentary Bouts, Standing and Different Types of Physical Activity during Working Days? A Cross-Sectional Accelerometer-Based Study among Blue-Collar Workers	PLOS ONE	11	5		10.1371/journal.pone.0154935	Analysis approach
Hörnberg	2021	Isotemporal Substitution of Time Between Sleep and Physical Activity: Associations With Cardiovascular Risk Factors in Early Rheumatoid Arthritis	ACR Open Rheumatology	3	3	138-146	10.1002/acr2.11225	Analysis approach
Hakimi	2024	Impacts of physical activity, sedentary behaviour, and sleep on depression symptoms in Canadian older adults 65¬†years of age and above: a compositional data analysis of the Canadian Longitudinal Study on Aging	Journal of Activity, Sedentary and Sleep Behaviors	3	1	8-Aug	10.1186/s44167-024-00047-7	Measure method
Hamilton,	2023	Daily and average associations of physical activity, social media use, and sleep among adolescent girls during the COVID-19 pandemic	Journal of Sleep Research	32	1		10.1111/jsr.13611	Analysis approach
Hargens	2021	Markers of poor sleep quality increase sedentary behavior in college students as derived from accelerometry.	Sleep Breath	25	1	537-544	10.1007/s11325-020-02190-2	Analysis approach

Haszard	2020	Non-Wear Time and Presentation of Compositional 24-Hour Time-Use Analyses Influence Conclusions About Sleep and Body Mass Index in Children.	Journal for the Measurement of Physical Behaviour	3	3	204-210	10.1123/jmpb.2019-0048	Study Design
Hidde	2024	Reallocating time to physical activity and sleep: Associations with body mass index in cancer survivors	International Journal of Behavioral Medicine	31	1	109-115	10.1007/s12529-023-10152-7	Analysis approach
Hidde	2022	Reallocating time to physical activity and sleep: associations with quality of life in cancer survivors	Supportive Care in Cancer	30	9	7527-7534	10.1007/s00520-022-07187-z	Analysis approach
Hinkley	2020	Prospective associations with physiological, psychosocial and educational outcomes of meeting Australian 24-Hour Movement Guidelines for the Early Years	The International Journal of Behavioral Nutrition and Physical Activity	17			10.1186/s12966-020-00935-6	Analysis approach
Hofman	2020	The composition of physical activity, sedentary behaviour and sleep duration across 24-hours: associations with self-rated sleep quality in middle-aged and elderly persons	Journal of Sleep Research	29		114-115		Publication type
Howie	2023	Physical Activity, Sedentary and Sleep Phenotypes in Women During the First Trimester of Pregnancy.	Maternal & Child Health Journal	27	10	1834-1845	10.1007/s10995-023-03745-x	Analysis approach
Huang	2024	Associations of 24-hour movement behaviors with emotional and behavioral problems among Chinese adolescents	The International Journal for Research in Social and Genetic Epidemiology and Mental Health Services				10.1007/s00127-024-02712-3	Analysis approach
Huang	2022	Sleep, physical activity, sedentary behavior, and risk of incident dementia: a prospective cohort study of 431,924 UK Biobank participants	Molecular Psychiatry	27	10	4343-4354	10.1038/s41380-022-01655-y	Measure method
Hulst	2022	Accelerometer-measured physical activity, sedentary behaviour, and sleep in children with cerebral palsy and their adherence to the 24-hour activity guidelines	Sleep Medicine	100		S190-S191		Analysis approach

Jakubec	2020	Is adherence to the 24-hour movement guidelines associated with a reduced risk of adiposity among children and adolescents?	BMC Public Health	20	1		10.1186/s12889-020-09213-3	Analysis approach
Janssen	2017	Is adherence to the Canadian 24-Hour Movement Behaviour Guidelines for Children and Youth associated with improved indicators of physical, mental, and social health?	Applied Physiology Nutrition and Metabolism	42	7	725-731	10.1139/apnm-2016-0681	Analysis approach
Jašková	2023	Compositional functional regression and isotemporal substitution analysis: Methods and application in time-use epidemiology	Statistical Methods in Medical Research	32	10	2064-2080	10.1177/09622802231192949	Study Design
Jauregui	2022	Movement behaviors during COVID-19 among Latin American/Latino toddlers and pre-schoolers in Chile, Mexico and the US	Scientific Reports	12	1		10.1038/s41598-022-23850-1	Analysis approach
Jiang	2024	24-hour movement behaviors time allocation and depression among Chinese community-dwelling older adults: A latent profile analysis	Geriatric Nursing	58		382-387	10.1016/j.gerinurse.2024.05.007	Analysis approach
Jindal	2021	The relationship of sleep duration and quality to energy expenditure and physical activity in children	Pediatric Obesity	16	6		10.1111/ijpo.12751	Analysis approach
Kahn	2021	Sleep, screen time and behaviour problems in preschool children: an actigraphy study.	European Child & Adolescent Psychiatry	30	11	1793-1802	10.1007/s00787-020-01654-w	behavior(s)
Khan	2021	Meeting 24-h movement guidelines and associations with health related quality of life of Australian adolescents	Journal of Science and Medicine in Sport	24	5	468-473	10.1016/j.jsams.2020.10.017	Analysis approach
Kim	2020	Examining the day-to-day bidirectional associations between physical activity, sedentary behavior, screen time, and sleep health during school days in adolescents	PLOS ONE	15	9		10.1371/journal.pone.0238721	Analysis approach
Knaeps	2018	Substituting sedentary time with light and moderate to vigorous physical activity is associated with better cardiometabolic health	Journal of Physical Activity & Health	15	3	197-203	10.1123/jpah.2017-0102	Analysis approach

Korcz	2023	Associations between physical activity, screen time, sleep time and selected academic skills in 8/9-year-old children.	BMC Public Health	23	1	11-Jan	10.1186/s12889-023-16230-5	Analysis approach
Kracht	2023	Association between Sleep, Sedentary Time, Physical Activity, and Adiposity in Adolescents: A Prospective Observational Study.	Med Sci Sports Exerc	55	1	110-118	10.1249/MSS.0000000000003018	Analysis approach
Kracht	2023	Association between Sleep, Sedentary Time, Physical Activity, and Adiposity in Adolescents: A Prospective Observational Study.	Medicine & Science in Sports & Exercise	55	1	110-118	10.1249/MSS.0000000000003018	Analysis approach
Kuzik	2022	Associations between meeting the Canadian 24-hour movement guidelines and physical, cognitive, social-emotional, and overall development in early childhood	Journal of Activity, Sedentary and Sleep Behaviors	1	1	2-Feb	10.1186/s44167-022-00002-4	Analysis approach
Larsen	2023	Substituting sedentary time with sleep or physical activity and subsequent weight-loss maintenance.	Obesity	31	2	515-524	10.1002/oby.23631	Analysis approach
Lee	2017	Meeting new Canadian 24-Hour Movement Guidelines for the Early Years and associations with adiposity among toddlers living in Edmonton, Canada	BMC Public Health	17	5	840-840	10.1186/s12889-017-4855-x	Analysis approach
Leppänen	2022	Longitudinal and cross-sectional associations of adherence to 24-hour movement guidelines with cardiometabolic risk	Scandinavian Journal of Medicine & Science in Sports	32	1	255-266	https://doi.org/10.1111/sms.14081	Analysis approach
Liang	2024	Associations of Reallocating Sedentary Time to Physical Activity and Sleep with Physical and Mental Health of Older Adults	Medicine & Science in Sports & Exercise	56	10	1935-1944	10.1249/MSS.0000000000003491	Measure method
Lin	2022	The mediating effect of engagement in physical activity over a 24-hour period on chronic disease and depression: Using compositional mediation model	Journal of Affective Disorders	299		264-272	10.1016/j.jad.2021.12.019	Measure method

Liu	2022	Meeting 24-h Movement Guidelines is Related to Better Academic Achievement: Findings from the YRBS 2019 Cycle	International Journal of Mental Health Promotion	24	1	13-24	10.32604/ijmhp.2021.017660	Analysis approach
Lopes	2020	Physical activity level sedentary behavior and sleep time associated to body composition in ilhabela schoolchildren	Medicine & Science in Sports & Exercise	52	7	573-573		Publication type
Lu	2024	Assessing the impact of 24-hour activity behaviors on cardiorespiratory fitness in the older adult: a componentAnalysis approach	Frontiers in Public Health	12			10.3389/fpubh.2024.1478533	Analysis approach
Magee	2013	Patterns of health behaviours predict obesity in Australian children	Journal of Paediatrics and Child Health	49	4	291-296	10.1111/jpc.12163	Analysis approach
Malheiros	2021	School schedule affects sleep, but not physical activity, screen time and diet behaviors.	Sleep Medicine	85		54-59	10.1016/j.sleep.2021.06.025	Analysis approach
Marshall	2023	Investigating the influence of physical activity composition on arterial stiffness in youth.	Eur J Sport Sci	23	4	617-624	10.1080/17461391.2022.2039304	Measure method
McGee M	2020	Lean mass accretion in children born very low birth weight is significantly associated with estimated changes from sedentary time to light physical activity.	Pediatr Obes	15	5	e12610	10.1111/ijpo.12610	Measure method
McGowan	2023	Meeting 24-hour movement behavior guidelines in young children: Improved quantity estimation and self-regulation	Early Education and Development	34	3	762-789	10.1080/10409289.2022.2056694	Analysis approach
McGregor	2019	Cox regression survival analysis with compositional covariates: Application to modelling mortality risk from 24-h physical activity patterns	Statistical Methods in Medical Research	29	5	1447-1465	10.1177/0962280219864125	Measure method
Mellow,	2019	Building your best day for healthy brain aging;The neuroprotective effects of optimal time use	Maturitas	125		33-40	10.1016/j.maturitas.2019.04.204	Study Design

Mellow,	2024	Should We Work Smarter or Harder for Our Health? A Comparison of Intensity and Domain-Based Time-Use Compositions and Their Associations With Cognitive and Cardiometabolic Health	Journals of Gerontology Series A-Biological Sciences And Medical Sciences	79	11		10.1093/gerona/glae233	Measure method
Meredith-Jones	2019	Do young children consistently meet 24-h sleep and activity guidelines? A longitudinal analysis using actigraphy	International Journal of Obesity	43	12	2555-2564	10.1038/s41366-019-0432-y	Analysis approach
Meyer	2020	Current and 1-Year Psychological and Physical Effects of Replacing Sedentary Time With Time in Other Behaviors.	Am J Prev Med	59	1	20-Dec	10.1016/j.amepre.2020.02.018	Analysis approach
Mitchell	2022	Device assessed physical activity, sedentary behaviour and sleep in relation to midlife cognition: Compositional analysis of the 1970 British cohort study	Journal of Epidemiology and Community Health	76		A30-A31	10.1136/jech-2022-SSMabstracts.62	Publication type
Mograss		Analysis of nighttime sleep fragmentation of actigraphy rest/activity patterns in healthy, young sedentary nappers.	Sleep Medicine	64		S261-S262	10.1016/j.sleep.2019.11.731	Publication type
Murray	2023	A time compositional analysis of the association between movement behaviors and indicators of mental health in young adults.	Scand J Med Sci Sports	33	12	2598-2607	10.1111/sms.14471	Measure method
Nauha	2024	Regularity of bedtime, wake-up time, and time in bed in mid-life: associations with cardiometabolic health markers with adjustment for physical activity and sedentary time	Journal of Activity, Sedentary and Sleep Behaviors	3	1	2-Feb	10.1186/s44167-023-00040-6	Analysis approach
Nielsen	2021	Physical Activity, Sedentary Behavior, and Sleep Before and After Bariatric Surgery and Associations with Weight Loss Outcome.	Obes Surg	31	1	250-259	10.1007/s11695-020-04908-3	Study Design
Orme	2020	Does cardiac rehabilitation for people with stroke in the sub-acute phase of recovery lead to physical behaviour change? Results from compositional analysis of accelerometry-derived data	Physiotherapy	107		234-242	10.1016/j.physio.2019.10.003	Measure method

Orme	2019	24-hour accelerometry in COPD: Exploring physical activity, sedentary behavior, sleep and clinical characteristics	International Journal of Chronic Obstructive Pulmonary Disease	14		419-430	10.2147/COPD.S183029	Analysis approach
Ortolá	2022	Associations of device-measured sleep, sedentariness and physical activity with growth differentiation factor 15 in older adults.	J Cachexia Sarcopenia Muscle	13	2	1003-1012	10.1002/jcsm.12924	Analysis approach
Ortolá	2020	Association of Physical Activity, Sedentary Behavior, and Sleep With Unhealthy Aging: Consistent Results for Device-Measured and Self-reported Behaviors Using Isotemporal Substitution Models	Journals of Gerontology Series A-Biological Sciences and Medical Sciences	76	1	85-94	10.1093/gerona/glaa177	Analysis approach
Ortolá	2021	Association of Physical Activity, Sedentary Behavior, and Sleep With Unhealthy Aging: Consistent Results for Device-Measured and Self-reported Behaviors Using Isotemporal Substitution Models	Journals of Gerontology Series A-Biological Sciences and Medical Sciences	76	1	85-94	10.1093/gerona/glaa177	Analysis approach
Padmapriya	2021	24-hour movement behaviour profiles and their transition in children aged 5.5 and 8,Äâyears ,Äì findings from a prospective cohort study	International Journal of Behavioral Nutrition and Physical Activity	18	1	145-145	10.1186/s12966-021-01210-y	Analysis approach
Park	2024	Reallocating time between device-measured 24-hour activities and cardiovascular risk in Asian American immigrant women: An isotemporal substitution model.	PLoS One	19	1	e0297042	10.1371/journal.pone.0297042	Analysis approach
Parker,	2024	The Day-Level Association Between Child Care Attendance and 24-Hour Movement Behaviors in Preschool-Aged Children	Journal of Physical Activity & Health	21	6	616-623	10.1123/jpah.2023-0656	Analysis approach
Patel	2023	Changes in Pediatric Movement Behaviors During the COVID-19 Pandemic by Stages of Lockdown in Ontario, Canada: A Longitudinal Cohort Study	Journal of Physical Activity & Health	20	4	292-302	10.1123/jpah.2022-0393	Analysis approach

Patte	2020	Are one-year changes in adherence to the 24-hour movement guidelines associated with depressive symptoms among youth?	BMC Public Health	20	1	793-793	10.1186/s12889-020-08887-z	Analysis approach
Pelclova	2024	The interplay between lying, sitting, standing, moving, and walking on obesity risk in older adults: a compositional and isotemporal substitution analysis	BMC Geriatrics	24	1		10.1186/s12877-024-05619-5	Measure method
Ramer	2022	The convergent effects of primary school physical activity, sleep, and recreational screen time on cognition and academic performance in grade 9	Frontiers in Human Neuroscience	16			10.3389/fnhum.2022.1017598	Analysis approach
Reddy	2024	Association of 24-hour movement behaviors and non-communicable diseases among adults of Udupi, Karnataka, India	International Journal of Health Promotion and Education				10.1080/14635240.2024.2410750	Analysis approach
Rees-Punia	2021	Composition of time in movement behaviors and weight change in Latinx, Black and white participants	PLOS ONE	16	1		10.1371/journal.pone.0244566	Measure method
Ren	2024	Associations of compositional time use with self-reported health, depression, and cognitive outcomes among Chinese adolescents	Children and Youth Services Review	158			10.1016/j.childyouth.2024.107488	Analysis approach
Richardson	2019	Study and Life: How first year university students use their time	Student Success	10	1	17-31	10.5204/ssj.v10i1.437	Analysis approach
Ringin	2024	Relative associations of behavioral and physiological risks for cardiometabolic disease with cognition in bipolar disorder during mid and later-life: Findings from the UK biobank	Psychological Medicine				10.1017/S0033291724000722	Analysis approach
Riquelme	2022	Association between 24-h movement guidelines and cardiometabolic health in Chilean adults	Scientific Reports	12	1		10.1038/s41598-022-09729-1	Analysis approach
Ronen	2018	Canadian Youth with Epilepsy: Time Spent on Physical Activity, Sedentary Screen-time and Sleep During 24 Hours	Annals of Neurology	84		S345-S345		Publication type
Rubin	2020	Prevalence and correlates of adherence to the combined movement guidelines among Czech children and adolescents	BMC Public Health	20	1	1692-1692	10.1186/s12889-020-09802-2	Analysis approach

Saevarsson	2021	Organized Sport Participation, Physical Activity, Sleep and Screen Time in 16-Year-Old Adolescents.	Int J Environ Res Public Health	18	6		10.3390/ijerph18063162	Analysis approach
Salas-Gonzalez	2023	Adherence to 24-h movement guidelines in Spanish schoolchildren and its association with insulin resistance: a cross-sectional study	Frontiers in Public Health	11				Analysis approach
Sampasa-Kanyinga	2021	24-Hour Movement Behaviors and Internalizing and Externalizing Behaviors Among Youth	Journal of Adolescent Health	68	5	969-977	10.1016/j.jadohealth.2020.09.003	Analysis approach
Sampasa-Kanyinga	2017	Associations between meeting combinations of 24-h movement guidelines and health-related quality of life in children from 12 countries	Public Health	153		16-24	10.1016/j.puhe.2017.07.010	Analysis approach
Santos	2017	Compliance with the Australian 24-hour movement guidelines for the early years: associations with weight status	BMC Public Health	17			10.1186/s12889-017-4857-8	Analysis approach
Sewell	2023	The interaction between physical activity and sleep on cognitive function and brain beta-amyloid in older adults	Behavioural Brain Research	437			10.1016/j.bbr.2022.114108	Analysis approach
Shi	2020	Compliance With 24-Hour Movement Guidelines in Hong Kong Adolescents: Associations With Weight Status	Journal of Physical Activity & Health	17	3	287-292	10.1123/jpah.2019-0230	Analysis approach
Skidmore	2017	The Association Between Objectively Measured Sleep, Physical Activity and Sedentary Behaviour on Cardiorespiratory Fitness and Health in 9 - 11 Year Old New Zealand Children.	Circulation	135		AMP030-AMP030	10.1161/circ.135.suppl_1.mp030	Publication type
Skinner	2024	Cross-sectional and longitudinal associations between the 24-hour movement behaviours, including muscle and bone strengthening activity, with bone and lean mass from childhood to adolescence	BMC Public Health	24	1		10.1186/s12889-024-17711-x	Analysis approach
Stalling	2020	Time allocation to active domains, physical activity, and health indicators in older adults: cross-sectional results from the OUTDOOR ACTIVE study	BMC Public Health	20	1		10.1186/s12889-020-09708-z	Analysis approach

Starbek	2022	The Impact of Online-Schooling during COVID-19 on Device-Measured 24-Hour Movement Behaviours among High School Students: A Compositional Data Analysis	Children-Basel	9	5		10.3390/children9050667	Outcome/no outcome
Suorsa	2024	Longitudinal Associations between 24-h Movement Behaviors and Cardiometabolic Biomarkers: A Natural Experiment over Retirement.	Med Sci Sports Exerc	56	7	1297-1306	10.1249/MSS.0000000000003415	Measure method
Suorsa	2023	Modifications of 24-h movement behaviors to prevent obesity in retirement: a natural experiment using compositional data analysis	International Journal of Obesity				10.1038/s41366-023-01326-0	Measure method
Swindell	2022	Prevalence and correlates of compliance with 24-h movement guidelines among children from urban and rural Kenya-The Kenya-LINX project	PLOS ONE	17	12		10.1371/journal.pone.0279751	Analysis approach
Tanaka	2020	Proportion of Japanese primary school children meeting recommendations for 24-h movement guidelines and associations with weight status	Obesity Research & Clinical Practice	14	3	234-240	https://doi.org/10.1016/j.orcp.2020.05.003	Measure method
Taylor	2021	Adherence to 24-h movement behavior guidelines and psychosocial functioning in young children: a longitudinal analysis.	Int J Behav Nutr Phys Act	18	1	110	10.1186/s12966-021-01185-w	Analysis approach
Taylor	2023	Associations of meeting 24-h movement behavior guidelines with cognitive difficulty and social relationships in children and adolescents with attention deficit/hyperactive disorder	Child and Adolescent Psychiatry and Mental Health	17	1	42-42	10.1186/s13034-023-00588-w	Analysis approach
Thewlis	2019	Objectively measured 24-hour activity profiles before and after total hip arthroplasty	Bone & Joint Journal	101B	4	415-425	10.1302/0301-620X.101B4.BJJ-2018-1240.R1	Analysis approach
Ting	2021	Relationship Between 24-hour Movement Behaviour & Physical Fitness Among Adolescents Using Compositional Data Analysis.	Medicine & Science in Sports & Exercise	53	8S	250-250	10.1249/01.mss.0000761972.53761.22	Publication type

Tomaz	2019	Body Mass Index, Physical Activity, Sedentary Behavior, Sleep, and Gross Motor Skill Proficiency in Preschool Children From a Low- to Middle-Income Urban Setting.	J Phys Act Health	16	7	525-532	10.1123/jpah.2018-0133	Analysis approach
Vaquero-Solis	2021	Health Promotion through Movement Behaviors and Its Relationship with Quality of Life in Spanish High School Adolescents: A Predictive Study	International Journal of Environmental Research and Public Health	18	14		10.3390/ijerph18147550	Analysis approach
Verloigne,	2021	Bidirectional associations between sedentary time and sleep duration among 12- to 14-year-old adolescents.	BMC Public Health	21	1	9-Jan	10.1186/s12889-021-11694-9	Analysis approach
Verswijveren	2022	Using compositional data analysis to explore accumulation of sedentary behavior, physical activity and youth health	Journal of Sport and Health Science	11	2	234-243	10.1016/j.jshs.2021.03.004	Measure method
Verswijveren	2022	Intervention effects on children's movement behaviour accumulation as a result of the Transform-Us! A school- and home-based cluster randomised controlled trial	International Journal of Behavioral Nutrition and Physical Activity	19	1		10.1186/s12966-022-01314-z	Study Design
Wang	2024	The mediating effect of 24-h time-use behaviors on the relationship between depression and mortality: A compositional mediation analysis for survival outcomes	Journal of Affective Disorders	350		222-229	10.1016/j.jad.2024.01.075	Measure method
Wang	2022	Physical Activity As Compositional Data: The Relationship Between Physical Activity, Sleep, Sedentary Time And Obesity	Medicine & Science in Sports & Exercise	54	9	471-471		Publication type
Wigna	2022	Relationship of Objectively Measured Physical Activity, Sedentary Behavior and Sleep Time with Cardiovascular and Metabolic Outcomes in Adolescents (A Pilot Study): ABCD Growth Study.	Maternal & Child Health Journal	26	11	2293-2299	10.1007/s10995-022-03471-w	Analysis approach
Wilkie	2016	Multiple lifestyle behaviours and overweight and obesity among children aged 9-11 years: results from the UK site of the International Study of Childhood Obesity, Lifestyle and the Environment	BMJ Open	6	2		10.1136/bmjopen-2015-010677	Analysis approach

Willems	2024	Cross-sectional associations between 24-hour movement behaviors and cardiometabolic health among adults with type 2 diabetes mellitus: A comparison according to weight status.	Journal of Science & Medicine in Sport	27	3	179-186	10.1016/j.jsams.2023.11.010	Measure method
Wu	2023	Analysis of the 24-h activity cycle: An illustration examining the association with cognitive function in the Adult Changes in Thought study	Frontiers in Psychology	14			10.3389/fpsyg.2023.1083344	Measure method
Wyszyńska	2021	Association between objectively measured body composition, sleep parameters and physical activity in preschool children: a cross-sectional study.	BMJ Open	11	1	e042669	10.1136/bmjopen-2020-042669	Analysis approach
Yuan	2023	Association Between Combinations of 24-Hour Movement Behaviors and Depression Among Adolescents - Inner Mongolia Autonomous Region, China, 2019-2021	China CDC Weekly	5	40		10.46234/ccdcw2023.169	Analysis approach
Yuan	2022	Daily Patterns of Accelerometer-Measured Movement Behaviors in Glaucoma Patients: Insights From UK Biobank Participants	Asia-Pacific Journal of Ophthalmology	11	6	521-528	10.1097/APO.0000000000000578	Analysis approach
Zarghani	2024	International study of 24-h movement behaviors of the early years (SUNRISE): A pilot study from Iran	Child Care Health and Development	50	3		10.1111/cch.13269	Analysis approach
Zawada	2024	Accelerometer-Measured Behavior Patterns in Incident Cerebrovascular Disease: Insights for Preventative Monitoring From the UK Biobank	Journal of the American Heart Association	13	11		10.1161/JAHA.123.032965	Analysis approach
Zeng	2022	Association between the 24-hour movement guidelines and executive function among Chinese children	BMC Public Health	22	1	1017-1017	10.1186/s12889-022-13420-5	Analysis approach
Zhang	2023	Beyond the bell: exploring the link between time allocation on extracurricular activities and academic performance in Chinese adolescents	International Journal of Adolescence and Youth	28	1		10.1080/02673843.2023.2277379	Measure method

Zhang	2024	Diurnal patterns of accelerometer-measured physical activity and sleep and risk of all-cause mortality: a follow-up of the National Health and Nutrition Examination Surveys (NHANES)	International Journal of Behavioral Nutrition and Physical Activity	21	1		10.1186/s12966-024-01673-9	Analysis approach
Zhao	2019	Visualising Combined Time Use Patterns of Children's Activities and Their Association with Weight Status and Neighbourhood Context.	Int J Environ Res Public Health	16	5		10.3390/ijerph16050897	Analysis approach
Zhao	2021	Lifestyle Clusters and Cardiometabolic Risks in Adolescents: A Chinese School-Based Study Using a Latent ClassAnalysis approach	Frontiers in Pediatrics	9			10.3389/fped.2021.728841	Analysis approach
Zhu	2024	Replacement of sedentary behavior with various physical activities and the risk of incident depression: A prospective analysis of accelerator-measured and self-reported uk biobank data	The International Journal for Research in Social and Genetic Epidemiology and Mental Health Services				10.1007/s00127-024-02708-z	Analysis approach
