# Supplementary material for: Characterizing device-measured sleep in observational health research using compositional data analysis: a systematic review
Source: Int J Behav Nutr Phys Act. 2026 Jun 15;23:78. doi: 10.1186/s12966-026-01935-8 (PMC13501660; doi:10.1186/s12966-026-01935-8)
Supplement: Supplementary file 4 [file 12966_2026_1935_MOESM4_ESM.pdf]

**Additional File 4:** Summary of device technical settings.

| Article | Sleep Device | Placement | Epoch* | Frequency | Visual Inspection/Manual Scoring | Software, Algorithm and/or Code | Sleep Log | Event Marker | Light Sensor | Score |
|---|---|---|---|---|---|---|---|---|---|---|
| Balbim 2024 (25) | Motion Watch8 | wrist | | | First full day manually removed | Rest-interval scoring algorithm of illuminance and motion data to classify time spent sleeping each night versus time spent awake each day in Motionware | | Each night when they started trying to sleep and again each morning when they finished trying to sleep | Scoring algorithm of including readings of illuminance | 10 |
| Bianchim 2022 (26) | ActiGraph GT9X Link GENEActiv | non-dominant wrist | | 100 Hz | Data were visually inspected to ensure that this was in accordance with the nocturnal sleep pattern for population of similar ages | Algorithm integrated within the GGIR package | | | | 8 |
| Biddle 2018 (27) | activPAL3 | thigh | | | | Algorithm in STATA | | | | 4 |
| Blodgett 2023 (28) | activPAL3 micro | midline upper thigh | | | | Information on thigh inclination and acceleration was used to derive body posture, and an algorithm separated out bouts of valid waking wear data from non-wear or sleep | | | | 4 |
| Blodgett 2024 (29) | ActivPAL3/4 ActiGraph | thigh | 1 seconds | | | Raw accelerometer data were centrally processed with software ActiPASS with algorithms for non-wear, sleep detection, posture, and activity intensity | | | | 6 |
| Brakenridge 2021 (30) | activPAL3 | right thigh | | 20 Hz | | Algorithmic method | If sleep and wake times were not reported in the diary they were estimated using an automated | | | 6 |

| | | | | | | | | | | |
|---|---|---|---|---|---|---|---|---|---|---|
| | | | | | | | algorithmic method | | | |
| Brakenridge 2024 (31) | activPAL3 | right thigh | | | | Algorithm to determine sleep and waking time | | | | 4 |
| Brayton 2023 (32) | ActiGraph GT9X Link | wrist | | | | Algorithm integrated within the GGIR package | | | | 4 |
| Cabanas-Sánchez 2021 (33) | ActiGraph GT9X | non-dominant wrist | | | | Algorithm integrated within the GGIR package | | | | 4 |
| Chen 2023 (34) | ActiGraph wGT3X-BT | right hipbone | 60 seconds | | | Algorithm integrated within the GGIR package | | | | 6 |
| Chong 2021 (35) | GENEActiv | non-dominant wrist | 5 seconds | 75 Hz | | Algorithm integrated within the GGIR package | | | | 8 |
| Collings 2023 (36) | ActiGraph GT3X+ | non-dominant wrist | 5 seconds | 30 Hz | | After download via the Actilife software R package GGIR | | | | 8 |
| Collings 2023 (37) | ActiGraph GT3X+ | non-dominant wrist | 5 seconds | 30 Hz | | Data were sampled at a frequency of 30 Hz, and Data were downloaded in the Actilife software and processed in R GGIR package | | | | 8 |
| Curtis 2020 (38) | GENEActiv | left wrist | 60 seconds | 50 Hz | Sleep and wake times were manually identified on the accelerometer trace | Algorithm integrated within the GGIR package | If the accelerometer was not worn overnight, sleep and wake times were taken from participant's sleep diaries | | | 10 |
| Curtis 2023 (39) | Fitbit Charge 3 | non-dominant wrist | | | | Fitbit's proprietary algorithm | | | Fitbit's proprietary algorithm used light data | 4 |

| | | | | | | | | | | |
|---|---|---|---|---|---|---|---|---|---|---|
| deFaria 2022 (40) | ActiGraph GT3X | Right hip | 15 seconds | 30 Hz | To calculate sleep time, evaluated daily graphs, inclinometer data, and converted these data into Microsoft Excel comma-separated values file | Data analyzed in ActiLife; To calculate sleep time, we evaluated daily graphs, inclinometer data, and converted these data into a Microsoft Excel comma-separated values file | Used to estimate sleep per night. | | | 12 |
| Domingues 2022 (41) | ActiGraph GT3X | right side of hip | 60 seconds | 30 Hz | | ActiLife with Sadeh algorithm | | | | 8 |
| Dumuid 2017 (42) | ActiGraph GT3X+ | right hip | 60 seconds | 80 Hz | | Fully automated algorithm specifically designed for ISCOLE | | | | 8 |
| Dumuid 2018 (43) | ActiGraph GT3X+ | right axillary line | 60 seconds | | Each full day of accelerometer data was visually inspected to determine discrepancies between self-reported sleep and movement recorded by the device | Actilife | The self-reported sleep times from the sleep diary were applied in a customized software program | | | 10 |
| Dumuid 2018 (44) | Actigraph GT3X+ | worn | 1 then 60 seconds | 80 Hz | | Application of a algorithm | | | | 8 |
| Dumuid 2018 (45) | Actigraph GT3X+ | right hip | 60 seconds | | | Data were downloaded in ActiLife using the low-frequency extension filter, with fully automated algorithm to estimate nocturnal sleep duration | | | | 6 |
| Dumuid 2019 (46) | GENEActiv | non-dominant wrist | 60 seconds | 50 Hz | Visual interpretation of trace, combined with activity card sleep/wake and device removal times to determine nonwear | | Visual interpretation of trace, combined with activity card sleep/wake and device removal times to determine nonwear | | | 10 |

| | | | | | | | | | | |
|---|---|---|---|---|---|---|---|---|---|---|
| Dumuid 2020 (47) | GENEActiv | non-dominant wrist | 60 seconds | 50 Hz | Visual inspection of daily accelerometer traces used in combination with other methods to identify sleep and non-wear time | Cobra, a MATLAB-based customized software program | Sleep and non-wear time were identified with self-reported data from the paper-based logs in combination with other methods | | | 12 |
| Dumuid 2021 (48) | GENEActiv | non-dominant wrist | 60 seconds | 50 Hz | Visual inspection of accelerometer traces and the paper-based logs were used to identify sleep and non-wear time | | Sleep and non-wear time were identified with self-reported data from the paper-based logs in combination with other methods | | | 10 |
| Dumuid 2022 (49) | GENEActiv | non-dominant wrist | 60 seconds | 100 Hz | Manual cross-checking of individual participant data by visually inspecting the acceleration trace averaged across 24 h in combination of recorded sleep logs | Raw acceleration data were downloaded using GENEActiv software and processed using a customized MATLAB graphic user interface (COBRA) | Manual cross-checking of individual participant data by visually inspecting the acceleration trace averaged across 24 h in combination of recorded sleep logs | | | 12 |
| Dumuid 2022 (50) | GENEActiv | wrist | 60 seconds | | | Classified into energy expenditure bands using Phillips cutpoints | Paper log used for bed/wake times and reasons for removal; Sport non-wear imputed with 50% MVPA, 30% LPA and 20% sedentary time | | | 8 |
| Fairclough 2017 (51) | ActiGraph GT9X | non-dominant wrist | | 30 Hz | | Sleep estimated from the ActiGraph raw accelerations and arm angle using algorithm in GGIR R package | | | | 8 |
| Fairclough 2021 (52) | ActiGraph GT9X | non-dominant wrist | 5 seconds | 100 Hz | | Algorithm integrated within the GGIR package | | | | 8 |

| | | | | | | | | | | |
|---|---|---|---|---|---|---|---|---|---|---|
| Fairclough 2023 (53) | ActiGraph GT9X | non-dominant wrist | 5 seconds | 100 Hz | | Algorithm integrated within the GGIR package | | | | 8 |
| Gupta 2020 (54) | Actigraph GT3X+ | right hip | | | Paper-based diary noting the start and end of work periods, time in bed,, non-wear time, and time of reference measurement; Time in bed measured using information from self-reported diary that was confirmed via visual checks of accelerometry data | | Paper-based diary noting the start and end of work periods, time in bed,, non-wear time, and time of reference measurement; Time in bed measured using information from self-reported diary that was confirmed via visual checks of accelerometry data | | | 6 |
| Healy 2021 (55) | ActiGraph GT9X Link | wrist | | | | Sleep duration estimated using a Sadeh algorithm | | | | 4 |
| Hofman 2022 (56) | GeneActiv | non-dominant wrist | | 50 Hz | | Validated algorithm to estimate nocturnal sleep | | | | 6 |
| Janda 2024 (57) | Children: ActiGraph GT3X+ Adolescents: GT9X Link | non-dominant wrist | 5 seconds | 100 Hz | | Algorithm integrated within the GGIR package | | | | 8 |
| Johansson 2020 (58) | ActiGraph GT3X+ | thigh and hip | | 30 Hz | Visually inspected the activity classification over time to identify and investigate any abnormalities in the data | MATLAB-software Acti4 was used to detect and derive the time spent in movement behavior types through an algorithm that uses inclinations and accelerations | Time in bed was defined based on a combination of accelerometer and diary data | | | 10 |
| Kim 2021 (59) | SenseWear Armband | arm | | | | Data were processed using the latest version of software, coupled with the | | | | 5 |

| | | | | | | | | | | |
|---|---|---|---|---|---|---|---|---|---|---|
| | | | | | | proprietary algorithms | | | | |
| Kuzik 2020 (60) | Actigraph wGT3X-BT | right hip | 15 seconds | 30 Hz | Sleep categorized through visual inspection guided by the log book and heuristics according to previous visual inspection literature | | Sleep categorized through visual inspection guided by the log book and heuristics according to previous visual inspection literature | | | 10 |
| Le 2022 (61) | ActiGraph wGT3X-BT | wrist | 60 seconds | | | Sleep duration and time awake in bed were scored following a protocol based on the Cole-Kripke scoring algorithm and daily sleep diaries | Sleep duration and time awake in bed were scored following a protocol based on the Cole-Kripke scoring algorithm and daily sleep diaries | | | 8 |
| Lee 2020 (62) | Actiwatch-2 | dominant hip | 60 seconds | | | Data files from both devices were obtained using standard algorithms for the waking and sleep periods | Sleep logs were completed, but role in scoring not described | | | 7 |
| Lu 2024 (63) | ActiGraph GT3X+ | right hip | 15 seconds | 30 Hz | | Estimation of sleep duration employed a polysomnography-validated accelerometer algorithm, which relied on the distribution of change in the z-angle | | | | 8 |
| Marmol-Perez 2024 (64) | ActiGraph wGT3x-BT | non-dominant wrist | | 90 Hz | | Raw data were processed using the GGIR R open-source package | Sleep log was used to assist the sleep-detection algorithm | | | 8 |
| Marshall 2022 (65) | GENEActiv | right wrist | 5 seconds | 20 Hz | | Sleep was determined according to the Van Hees sleep algorithm as no arm angle change of >5 degrees or 5+ min | | | | 8 |

| | | | | | | | | | | |
|---|---|---|---|---|---|---|---|---|---|---|
| Matricciani 2021 (66) | GENEActiv | non-dominant wrist | 60 seconds | | Visual inspection of the activity trace used to correct sleep times and in cases where sleep times were not reported | van Hees sleep algorithm was used to detect sleep and wake between self-reported bedtime and wake times | Activity monitor cards were then used to identify children's bed times, wake times, and device removals | | | 10 |
| Mellow 2022 (67) | Axivity AX3 | non-dominant wrist | | 100 Hz | Time spent in sleep was verified by manual cross-checking of sleep logs and visual inspection of the accelerometry trace | Raw acceleration data were downloaded using the Open Movement GUI software and further processed using a custom MATLAB graphic user interface developed at the University of South Australia (COBRA) | Time spent in sleep was verified by manual cross-checking of sleep logs and visual inspection of the accelerometry trace | | | 10 |
| Mellow 2024 (68) | Axivity AX3 | non-dominant wrist | | 100 Hz | Manually marking of wake and sleep times for each 24-hr recording period while cross-checking participants' sleep logs against the accelerometry trace | Raw acceleration data were downloaded using the Open Movement GUI software and further processed using a custom MATLAB graphic user interface developed at the University of South Australia (COBRA) | Sleep duration and time awake in bed were scored following a protocol based on the Cole-Kripke scoring algorithm and daily sleep diaries | | | 8 |
| Migueles 2020 (69) | ActiGraph GT3X+ | non-dominant wrist | | 100 Hz | | Raw accelerations were downloaded via the ActiLifesoftware and processed in the R package GGIR | Sleep time was identified using an automated algorithm guided by the time reported by the participants | | | 9 |
| Migueles 2023 (70) | ActiGraph GT3X+ | non-dominant wrist | 5 seconds | 50 Hz | | Sleep and awake periods were identified using an automated algorithm | | | | 8 |
| Mitchell 2023 (71) | activPAL3 micro | thigh | | | | | | | | 2 |
| Murphy 2024 (72) | GENEActiv | non-dominant wrist | 5 seconds | 50 Hz | Visual inspection of accelerometer traces in combination with other methods | Data imported into a MATLAB customised software program (Cobra) where activity cards, visual inspection of accelerometer traces and the van Hees algorithm were used | Activity cards were used in combination with other methods to detect sleep and nonwear times | | | 12 |

| | | | | | | | | | | |
|---|---|---|---|---|---|---|---|---|---|---|
| | | | | | | to identify instances of non-wear and sleep | | | | |
| Ng 2021 (73) | GENEActiv | non-dominant wrist | 60 seconds | 50 Hz | A research assistant manually checked each participant, daily accelerometry traces and, if required, moved the sleep onset and sleep offset markers to match the trace. | Data downloaded in GENEActiv PC software and converted to 60-s epoch files and visualized in a MATLAB-based program (Cobra) with the van Hees sleep algorithm was used | Markers indicating self-reported sleep (sleep onset) and wake (sleep offset) times from the activity logs were superimposed onto the daily accelerometer traces | Markers indicating self-reported sleep (sleep onset) and wake (sleep offset) times from the activity logs were superimposed onto the daily accelerometer traces | | 10 |
| Niemelä 2024 (74) | Oura Ring Generation 2 | non-dominant wrist | 60 seconds | 250 Hz & 50 Hz | | Start and end of bedtimes were recognized with Oura's own algorithms | | | | 8 |
| Oviedo-Caro 2020 (75) | Sensewear Mini Armband | arm | | | | | | | | 0 |
| Powell 2020 (76) | ActiviPAL3 Micro | right thigh | 15 seconds | 20 Hz | Sleep was calculated as [time between 0500 h and the first non-sedentary epoch]+[time between the last non-sedentary epoch and the following 0500 h time point] | | | | | 8 |
| Rasmussen 2023 (77) | ActiGraph wGT3X-BT AcitGraph GT9X Link | non-dominant wrist | | 100 Hz | | van Hees algorithm in GGIR R package | Algorithm guided by the participant sleep logs | | | 8 |

| | | | | | | | | | |
|---|---|---|---|---|---|---|---|---|---|
| Rorem 2024 (78) | ActiGraph GT3X-BT | non-dominant wrist | 5 seconds | 30 Hz | Sleep defined using a combination of sleep logs and manual cross-checking of individual participants by visually inspecting the acceleration trace averaged across 24 hours | Visual inspection of sleep analysis graphs in Actilife software with the support of the log | Sleep defined using a combination of parent completed sleep logs and manual cross-checking of individual participants by visually inspecting the acceleration trace averaged across 24 hours | | | 12 |
| Runacres 2023 (79) | ActiGraph GT3X | right hip | | 100 Hz | | Sleep calculated using the Sadeh algorithms | Children and adolescents also completed a seven-day log to detail periods when the monitor was removed, waking time and time going to bed, to minimise the misclassification of non-wear time as sedentary time or sleep | | | 8 |
| Sandborg 2022 (80) | ActiGraph wGT3x-BT | wrist | 5 seconds | 100 Hz | | GGIR package in R | To assist the sleep-detection algorithm, women were instructed to report their everyday in bed and out of bedtime | | | 10 |
| Smith 2021 (81) | GENEActiv | non-dominant wrist | 60 seconds | 100 Hz | Time spent in sleep was defined using a combination of sleep logs and manual cross-checking of individual participants by visually inspecting the acceleration | Data were downloaded in GENEActiv PC software and processed using customized MATLAB graphic user interface | Time spent in sleep was defined using a combination of sleep logs and manual cross-checking of individual participants by visually inspecting the | | | 12 |

| | | | | | | | | | | |
|---|---|---|---|---|---|---|---|---|---|---|
| | | | | | trace averaged across 24 hours | | acceleration trace averaged across 24 hours | | | |
| Smith 2021 (82) | GENEActiv | wrist | 60 seconds | | | An automatized algorithm and sustained inactivity periods in accord with the work of Van Hees and colleagues | | | | 6 |
| Song 2018 (83) | ActiGraph GT3X | wrist | 60 seconds | | Data evaluated by a scorer with standardized protocol that assessed ambient light intensity and physical activity level from the accelerometer and self-report sleep diary information to determine the time in bed and time out of bed | The Cole-Kripke algorithm was applied to identify actual sleep minutes between time in bed and time out of bed | Data evaluated by a scorer with standardized protocol that assessed ambient light intensity and physical activity level from the accelerometer and self-report sleep diary information to determine the time in bed and time out of bed | | Data evaluated by a scorer with standardized protocol that assessed ambient light intensity and physical activity level from the accelerometer and self-report sleep diary information to determine the time in bed and time out of bed | 10 |
| St Laurent 2023 (84) | Actiwatch Spectrum | non-dominant wrist | 15 seconds | 32 Hz | Rest intervals for overnight periods and daytime naps were defined using a combination of marked events and sleep diaries, or manually scoring (the first three consecutive minutes of sleep were used to define sleep onset and the last five consecutive minutes of sleep were defined as sleep offset) | Actiware software and default algorithm | Rest intervals for overnight periods and daytime naps (when present) were defined using a combination of marked events and sleep diaries | Rest intervals for overnight periods and daytime naps (when present) were defined using a combination of marked events and sleep diaries | | 14 |

| | | | | | | | | | | |
|---|---|---|---|---|---|---|---|---|---|---|
| Swindell 2020 (85) | ActiSleep+ | right hip | 60 seconds | | | A fully-automated algorithm developed for use with 24-h waist-worn accelerometer | | | | 6 |
| Talarico 2018 (86) | Actical | right hip | 15 seconds | | The sleep times recorded in the logs were manually verified, and corrected as necessary, by visually inspecting the recorded times against the accelerometer data | | | Rest intervals for overnight periods and daytime naps were defined using a combination of marked events and sleep diaries | | 6 |
| Tan 2023 (87) | ActiGraph GT3X+ BT | non-dominant wrist | | 80 Hz | | Data were extracted ActiLife software and processed GGIR R package | | | | 6 |
| Taylor 2018 (88) | Actical | waist | 15 seconds | | | Data cleaned and scored with automated script in MATLAB | | | | 6 |
| Taylor 2020 (89) | ActiGraph GT3X | right hip | 15 seconds | | | Data cleaned and scored with automated script in MATLAB | | | | 6 |
| Taylor 2023 (90) | Actical | waist | 15 seconds | | | Data scored with automated algorithm that | | | | 6 |
| Tyler 2022 (91) | ActiGraph GT9X | non-dominant wrist | 5 seconds | 100 Hz | | Sleep estimated with accelerometer algorithm | | | | 8 |
| Vanderlinden 2023 (92) | ActiGraph wGT3X-BT | non-dominant wrist | | | | Data processed with algorithms in Actilife software | | | | 4 |
| Verhoog 2020 (93) | GENEActiv | non-dominant wrist | | 50 Hz | | Data processed in Python with PAMPRO software | The algorithm used information on overnight sleep periods from the sleep diary and subtracted from total sedentary behavior | | | 6 |

| | | | | | | | | | | |
|---|---|---|---|---|---|---|---|---|---|---|
| Walmsley 2022 (94) | Axivity AX3 | dominant wrist | | | Using camera images and time use diaries, trained annotators annotated accelerometer data with labels from the Compendium of Physical Activities, including sleep | | Using camera images and time use diaries, trained annotators annotated accelerometer data with labels from the Compendium of Physical Activities, including sleep | | | 4 |
