# Supplementary material for: Characterizing device-measured sleep in observational health research using compositional data analysis: a systematic review
Source: Int J Behav Nutr Phys Act. 2026 Jun 15;23:78. doi: 10.1186/s12966-026-01935-8 (PMC13501660; doi:10.1186/s12966-026-01935-8)
Supplement: Supplementary file 5 [file 12966_2026_1935_MOESM5_ESM.pdf]

Additional File 5. Summary of sleep period specifications.

Article	Wear Period	Minimum Valid days	Types of Days	Valid Day Criteria	Non-wear Detected/Defined	Non-wear Addressed	Score
Balbim 2024 (25)	7 consecutive days	5 days	Averaged the time across the recording period	16 hours/24 hour period & had wake and sleep data	(Not reported)	(Not reported)	8
Bianchim 2022 (26)	7 consecutive days	3 nights	(Not reported)	16 hours/day	Procedure to detect non-wear time is described in detail elsewhere	(Not reported)	7
Biddle 2018 (27)	7 consecutive days	4 days	(Not reported)	At least 10 h of waking wear data, >500 steps, and <95% spent in any one behaviour	Heat-maps of data were examined to identify any non-wear and invalid data that may have occurred on valid days	Excluded	12
Blodgett 2023 (28)	7 consecutive days	(Not reported)	(Not reported)	(Not reported)	An algorithm separated out bouts of valid waking wear data from non-wear or sleep	(Not reported)	5
Blodgett 2024 (29)	7 days	1 day	(Not reported)	20+ hours of wear/day, 1 period of waking detection, and >0 min of sleep	ActiPASS algorithm for non-wear	(Not reported)	10
Brakenridge 2021 (30)	7 consecutive days	(Not reported)	Across all valid days	Monitor wear time at least 80% of waking hours, or at least 10 hours if the participant's diary was missing sleep and wake times	(Not reported)	(Not reported)	6
Brakenridge 2024 (31)	8 consecutive days	1 day	Across valid wear days	14 hours of waking time	(Not reported)	(Not reported)	7
Brayton 2023 (32)	(Not reported)	(Not reported)	(Not reported)	At least 16 hours of valid data	(Not reported)	(Not reported)	1
Cabanas-Sánchez 2021 (33)	7 consecutive days	4 days	At least one weekend day	At least 16 hours of valid data	(Not reported)	(Not reported)	5
Chen 2023 (34)	7 consecutive days	4 nights of sleep	At least one weekend night	Recorded sleep at least 220 min	(Not reported)	(Not reported)	8

Chong 2021 (35)	6 consecutive days	3 days	At least one weekend day	At least 16 hours of wear with at least 200 min sleep & less than 1000 min of sedentary time	Estimated based on the standard deviation and value range of each accelerometer axis, calculated over 60 min windows with 15 min increments	For each 15 min period detected as non-wear time over the valid wearing days, missing data were imputed using the mean values calculated from valid data at the same time points on other days	14
Collings 2023 (36)	One week	4 days	At least one weekend	At least 10 hours of data	(Not reported)	(Not reported)	5
Collings 2023 (37)	1 week	4 days	At least 1 weekday	At least 10 hours of data	(Not reported)	Excluded	6
Curtis 2020 (38)	7 days	4 days	At least one weekend day	At least 10 hours of data	Identification of nonwear during sleep from sleep log	Excluded	11
Curtis 2023 (39)	13 months (1 month selected)	3 days	At least one weekend day	At least 18 hours wear time including a sleep period	Minutes determined as sedentary (logged when other activities were not identified) and with absent heart rate data were classified as non-wear	Excluded	14
deFaria 2022 (40)	8 consecutive days	6 days and 3 nights	At least one weekend night	At leaset 10 hours during day (without considering sleep)	(Not reported)	(Not reported)	7
Domingues 2022 (41)	8 consecutive days	3 nights of 'proper sleep'	At least one weekend night	At least 10 hours of wakefulness	(Not reported)	(Not reported)	7
Dumuid 2017 (42)	7 days	3 nights	(Not reported)	At least 160 min total sleep period	Any sequence of ≤20 consecutive minutes of 0 activity counts)	Excluded	11
Dumuid 2018 (43)	7 days	4 days	At least 3 weekdays and 1 weekend day	At least 600 min waking wear time per day	(Not reported)	(Not reported)	5
Dumuid 2018 (44)	7 days	3 nights	At least 3 weekdays & 1 weekend night	At least 10 hours per daily waking wear time and 160 min total sleep time for at least three nights	(Not reported)	(Not reported)	8

Dumuid 2018 (45)	7 days	4 days	At least one weekend day	At least 10 hours of waking wear/day	Awake non-wear time was any sequence of 20+ consecutive minutes of 0 activity counts	Excluded	8
Dumuid 2019 (46)	8 days	4 days	(Not reported)	Waking wear time at least 10 hours, daily average sleep duration at least 200 minutes, & sedentary time less than 1000 minutes	Visual interpretation of trace combined with activity card sleep/wake and device removal times	Excluded	11
Dumuid 2020 (47)	8 days	4 days	(Not reported)	Waking wear time at least 10 hours, daily average sleep duration at least 200 minutes, & sedentary time less than 1000 minutes	Identified through visual inspection of daily accelerometer traces and logs	Wake missing for sport was imputed	10
Dumuid 2021 (48)	8 days	4 days	(Not reported)	Waking wear time at least 10 hours, daily average sleep duration at least 200 minutes, & sedentary time less than 1000 minutes	Visual inspection of accelerometer traces and the paper-based logs	Wake missing for sport was imputed	8
Dumuid 2022 (49)	7 consecutive days	4 days	At least 3 weekdays & 1 valid weekend day	At least 10 waking hours	(Not reported)	(Not reported)	5
Dumuid 2022 (50)	7 days	4 days	(Not reported)	Waking wear time at least 10 hours, daily average sleep duration at least 200 minutes, & sedentary time less than 1000 minutes	(Not reported)	(Not reported)	6
Fairclough 2017 (51)	7 days	3 days	Any three days	At least 16 hours	Estimated on the basis of the standard deviation and value range of each accelerometer axis, calculated for moving windows of 60 min with 15 min increments	Wake: For each 15 min period detected as non-wear time over the valid days, missing data were replaced by the mean value calculated from measurement on other days at the same time of day	9

Fairclough 2021 (52)	7 days	(Not reported)	(Not reported)	(Not reported)	Determined based on the standard deviation and value range of the accelerations at each axis, calculated for 60-minute windows with a 15-minute sliding window - If for at least 2 out of the 3 axes the SD was less than 13 mg or the value range was less than 50 mg, the time window was classified as non-wear	Imputed non-wear data by the average at similar time points on other days of the week	6
Fairclough 2023 (53)	7 days	4 days	At least 3 weekdays & 1 weekend day	Full 24-hour data	Signal processing in GGIR	Imputed by the aver-age at similar time points on other days of the week	11
Gupta 2020 (54)	4 consecutive days	1 day	At least 2 working days	At least 4 hours or 75% of the worker's average work and leisure time & time in bed was at least 4 h in duration	Recognized according to an automatic procedure	Excluded	11
Healy 2021 (55)	7 days & nights	4 days	(Not reported)	(Not reported)	Identified periods of 60 + minutes of zero counts	Excluded	9
Hofman 2022 (56)	7 consecutive & nights	4 days	(Not reported)	At least 1200 min of data each day	May only be wake: Defined as all time periods where the standard deviation of acceleration in each of the three axes fell below 13 mg for over 1 hour	Excluded	10
Janda 2024 (57)	7 consecutive days	4 days	At least 3 school days and at least one weekday day	At least 16 hours per day	GGIR package with default setting used for non-wear time detection	Missing data were imputed average values from the same time points on other days	9
Johansson 2020 (58)	7 consecutive days	5 days	(Not reported)	At least 16 hours per 24-hr period	Defined by diary information and visual inspection of the activity classification and in Acti4 automatically using a set of rules	Excluded	10

Kim 2021 (59)	2 randomly selected days	Not clearly reported	Not clearly reported	(Not reported)	(Not reported)	(Not reported)	4
Kuzik 2020 (60)	7 days	3 days	(Not reported)	At least 10 hours per day of waking day wear time	Defined as 20+ minutes consecutive 0 counts	Excluded	6
Le 2022 (61)	15 days	(Not reported)	(Not reported)	(Not reported)	Determined from nights during which the device was not worn or missing sleep diary and unable to identify clear sleep onset and offset times from the accelerometry data	Excluded	7
Lee 2020 (62)	7 consecutive days	(Not reported)	(Not reported)	(Not reported)	(Not reported)	(Not reported)	2
Lu 2024 (63)	8 consecutive days	4 days	At least 3 weekdays and 1 weekend day	At least 10 hours per day	Defined as periods of 60 consecutive minutes with zero counts	Excluded	9
Marmol-Perez 2024 (64)	7 consecutive days	1 day	Across all valid days	At least 16 hours (and accelerometer registered 23 hours)	Detected based on the SD of the raw accelerations recorded in the three accelerometer axes	Imputed by means of the acceleration in the rest of the days at the same time window	9
Marshall 2022 (65)	28 consecutive days	4 days	At least 3 weekdays & 1 weekend day	At least 16 hours per day	"Wear time criteria was applied"	Excluded	6
Matricciani 2021 (66)	8 days	4 nights	At least 1 non-school night	Average sleep time 200+ min	Activity monitor cards used	Only included if sleep citeria met; Wake: If non-wear period was removed for "sport" and four hours or less, the removal period was replaced with MVPA	11
Mellow 2022 (67)	7 consecutive days	4 days	At least 3 weekdays & 1 weekend day	At least 10 waking hours	Identified as 60+ min of 25 g/min or less and manually verified against participant diaries and visual inspection of the accelerometry trace	Excluded	13

Mellow 2024 (68)	7 consecutive days	4 days	At least 3 weekdays & 1 weekend day	At least 10 waking hours and less than 8 hours of non-wear in 24-hour period	(Not reported)	Excluded	6
Migueles 2020 (69)	7 consecutive days	4 days	At least 1 weekend day	At least 16 hours per day	(Not reported)	Excluded in valid day criteria not met; Non-wear time (and abnormal high accelerations related to malfunctioning) were imputed by average acceleration during the same time interval from the rest of the days	5
Migueles 2023 (70)	7 days	1 day	(Not reported)	At least 16 hours	Identified from the magnitude and variability of the raw accelerations measured at each accelerometer axis	Excluded	7
Mitchell 2023 (71)	7 days	1 day	(Not reported)	At least 10 uninterrupted hours	(Not reported)	(Not reported)	4
Murphy 2024 (72)	2 weeks	4 days	At least 1 weekend day	At least 10 hours during wake & 200+ min sleep time	Visual inspection of accelerometer traces and the van Hees algorithm	Excluded	14
Ng 2021 (73)	8 days	4 days	At least 1 weekend day	At least 10+ waking hours, 200+ min sleep, & less than 1000 min sedentary time	(Not reported)	Excluded	10
Niemelä 2024 (74)	14 days	4 nights	(Not reported)	Sleep recordings and at least 600 min during wake	(Not reported)	Excluded	8
Oviedo-Caro 2020 (75)	9 days	7 days	At least 5 weekdays and 2 weekend days	At least 95% of the day (1368 minutes)	(Not reported)	Excluded	10
Powell 2020 (76)	7 consecutive days	4 days	At least 1 weekday and 1 weekend day	At least 10 hours of waking data	Period of 60+ min of consecutive zero counts, which was cross-referenced with any self-reported non-wear time	Excluded	9

Rasmussen 2023 (77)	7 consecutive days	4 days	At least 1 weekend day	At least 16 hours	(Not reported)	Excluded	6
Rorem 2024 (78)	7 days	1 day	(Not reported)	At least 10 hours of wake time at both time points	240+ consecutive zero counts	Excluded	8
Runacres 2023 (79)	7 consecutive days	3 days	Any 3 days	At least 8 hours	(Not reported)	Excluded	6
Sandborg 2022 (80)	7 consecutive 24-hour periods	1 day	(Not reported)	At least two-thirds of 24-hour period including wear time and two-thirds of the wake time being wear time	(Not reported)	Excluded	5
Smith 2021 (81)	7 consecutive days	4 days	At least 3 week days and 1 weekend day	At least 10 waking hours	(Not reported)	Excluded	6
Smith 2021 (82)	7 days	4 days	At least 1 weekend day	(Not reported)	Aat least 60 minutes of consecutive movement counts of 0, allowing for up to 2 minutes of movement counts between 0 and 100	Excluded	8
Song 2018 (83)	7 days	3 days	(Not reported)	Complete sleep and at least 10 hours of non-sleep wear time	Non-wear algorithms to applied to periods of non-sleep hours	Excluded	9
St Laurent 2023 (84)	16 days	3 nights	(Not reported)	At least 3 nights with sleep data and 3 days with 480+ minutes waking data	Device off-wrist detection	Excluded	12
Swindell 2020 (85)	7 consecutive days	4 days	At least 1 weekend day	At least 10 hours of waking wear time	60+min of consecutive zeros with the allowance of interruptions for up to 2 min	Excluded	10
Talarico 2018 (86)	7 days	4 days	(Not reported)	At least 10 hours per day of waking day wear time	60+min of consecutive zeros with the allowance of interruptions for up to 2 min and non-wear time recorded on the log	Excluded	8

Tan 2023 (87)	7 days	3 days	At least one weekend day	At least 10 hours of wear time	Estimated based on standard deviations and value range of each accelerometer axis at 60-min windows in 15-min increments	Excluded if not valid day; Of the valid days, non-wear time in blocks of 15 min were imputed based on the mean value from other valid days	9
Taylor 2018 (88)	7 days	3 days	Any 3 days	At least 20 hours of data	20+ min of consecutive zeros during awake time data only	Excluded	10
Taylor 2020 (89)	8 days	3 days	Any 3 days	At least 20 hours of data	20+ min of consecutive zeros during awake time data only	Excluded	10
Taylor 2023 (90)	1 week	3 days	(Not reported)	At least 20 hours of data	20+ min of consecutive zeros during awake time data only	Excluded	9
Tyler 2022 (91)	7 days	3 days	Averaged over days	At least 16 hours per day	"Identified non-wear", but method not reported	Excluded if not valid day or post-calibration error was 10+ mg	7
Vanderlinden 2023 (92)	6 consecutive days and 5 nights (including two weekend days)	4 days	(Not reported)	At least 10 hours of waking wear time	(Not reported)	Excluded	5
Verhoog 2020 (93)	7 consecutive days and nights	(Not reported)	(Not reported)	(Not reported)	Estimated as time periods where the SD of acceleration in all 3 axes fell below 13 mg for >1 hour	Excluded	6
Walmsley 2022 (94)	7 days	3 days	(Not reported)	Had data in each 1-hour period of the 24-hour cycle	Unbroken episodes of at least 60 min during which SD of each axis of acceleration was less than 13.0mg	After invalid days excluded, non-wear imputed as the mean behavior in the corresponding minute of the day on remaining days	7
