# Supplementary material for: Characterizing device-measured sleep in observational health research using compositional data analysis: a systematic review
Source: Int J Behav Nutr Phys Act. 2026 Jun 15;23:78. doi: 10.1186/s12966-026-01935-8 (PMC13501660; doi:10.1186/s12966-026-01935-8)
Supplement: Supplementary file 6 [file 12966_2026_1935_MOESM6_ESM.pdf]

Additional File 6. Summary of sleep conceptualization, integration in time-use compositions, and discussion.

Article	Sleep Conceptualization					Time-Use					Discussion	
	Concep-tualization	Measured Metrics	Definitions	Cycle Defined	Score	Closure	# of Parts	# Sleep Parts	Components	Score	Sleep Limitations	Score
Balbim 2024 (25)	Other: Major sleep period	Bedtime<br>Wake time<br>Time in bed<br>WASO	(Not reported)	Bedtime to bedtime	4	24 h	4	1	Sedentary behavior<br>Sleep duration<br>Light PA<br>MVPA	2	Did not account for wear-time on week and weekend days as inclusion criteria for data analysis; Did not collect self-report measures of 24-HAC behaviors; Did not account for napping in our estimates of sleep, which was indexed as sedentary.	2
Bianchim 2022 (26)	Night	Sleep unspecified	Sleep time = any period of sustained inactivity, defined as no change of more than five degrees in the monitor angle during a nocturnal sleep window	(Not reported)	4	24 h	4	1	Sedentary time<br>Sleep (unspecified)<br>Light PA<br>MVPA	2	(Not reported)	0
Biddle 2018 (27)	Night	Time in bed	Sleep time is inferred by subtracting waking wear time from 24 hours	(Not reported)	4	24 h	4	1	Sleep (unspecified)<br>Sitting<br>Standing<br>Stepping	2	Inability to measure sleep directly within the activPAL data	2
Blodgett 2023 (28)	24-hour/daily cycle	Sleep duration	Sleep was classified as the longest reclining bout between noon and noon each day of at least 2 hours or any long bouts lasting at least 5 hours	Noon to noon	4	(Not reported)	4	1	Sedentary behavior<br>Sleep (unspecified)<br>Light PA<br>MVPA	0	Sleep time may have been overestimated as the device did not capture biological sleep	2

Blodgett 2024 (29)	24-hour/daily cycle	Time in bed<br>Sleep duration	(Not reported)	(Not reported)	2	24 h	5	1	Sedentary behavior<br>Sleep (unspecified)<br>Light PA<br>MVPA<br>Standing	2	Despite clear advances in the ActiPASS-based detection of activity intensity and SB, sleep time may have been overestimated as time spent in bed rather than biological sleep	2
Brakenridge 2021 (30)	24-hour/daily cycle	Total sleep time	(Not reported)	(Not reported)	2	24 h	4	1	Sleep (unspecified)<br>Sitting<br>Standing<br>Stepping	2	CoDA modeling did not account for sleep quality; There may have been some errors in the sleep estimate as sleep onset was self-reported or automatically estimated rather than objectively measured	2
Brakenridge 2024 (31)	24-hour/daily cycle	Time in bed<br>Total sleep time	(Not reported)	(Not reported)	0	(Not reported)	5	1	Sleep (unspecified)<br>Light PA<br>MVPA<br>Sitting<br>Standing	0	(Not reported)	0
Brayton 2023 (32)	24-hour/daily cycle	Sleep onset<br>Sleep offset<br>Total sleep time<br>Sleep efficiency	(Not reported)	(Not reported)	0	(Not reported)	4	1	Sedentary time<br>Sleep (unspecified)<br>Light PA<br>MVPA	0	(Not reported)	0
Cabanas-Sánchez 2021 (33)	24-hour/daily cycle	Sleep period	(Not reported)	(Not reported)	2	24 h	4	1	Sedentary time<br>Sleep (unspecified)<br>Light PA<br>MVPA	2	(Not reported)	0
Chen 2023 (34)	24-hour/daily cycle	Sleep onset<br>Sleep offset<br>Total sleep time	(Not reported)	(Not reported)	2	24 h	4	1	Sedentary behavior<br>Sleep duration<br>Light PA<br>MVPA	2	(Not reported)	0
Chong 2021 (35)	24-hour/daily cycle	Wake time<br>Sleep period<br>Sleep duration	(Not reported)	(Not reported)	0	(Not reported)	4	1	Sedentary time<br>Sleep duration<br>Light PA<br>MVPA	0	(Not reported)	0

Collings 2023 (36)	Night	Sleep period<br>Total sleep time<br>Sleep duration	Total sleep period = difference between sleep onset (hours before midnight) and offset times (hours since midnight)	(Not reported)	4	24 h	4 and 5	1	Sedentary time<br>Sleep (unspecified)<br>Light PA<br>MVPA<br>Prolonged sedentary time<br>Non-Prolonged sedentary time	2	Sleep period (which includes brief waking intervals overnight) was measured as opposed to duration	2
Collings 2023 (37)	Unclear	Sleep period	Total sleep period = difference between sleep onset and waking times	(Not reported)	4	24 h	4 and 5	1	Sedentary time<br>Sleep (unspecified)<br>Light PA<br>MVPA<br>Prolonged sedentary time<br>Non-prolonged sedentary bouts	2	Investigated the sleep period (which includes brief waking intervals overnight) as opposed to sleep duration	2
Curtis 2020 (38)	Night	Bedtime<br>Wake time<br>Sleep onset<br>Sleep period<br>Sleep duration	(Not reported)	(Not reported)	3	24 hours	4	1	Sedentary behavior<br>Sleep duration<br>Light PA<br>MVPA	2	Reallocations of sleep might be associated with health and well-being for a mean composition indicating insufficient sleep	2
Curtis 2023 (39)	Unclear	Sleep period	(Not reported)	(Not reported)	3	24 h	4	1	Sedentary behavior<br>Sleep duration<br>Light PA<br>MVPA	2	Fitbits may use different cut points to classify activities than research accelerometers, making comparison of results more difficult	1
deFaria 2022 (40)	Night	Bedtime<br>Wake time<br>Time in bed<br>Sleep period<br>Total sleep time<br>Sleep duration	Detected the moment when the teenager woke up in the morning and went to sleep at night	(Not reported)	3	24 h	4	1	Sedentary behavior<br>Sleep (unspecified)<br>Light PA<br>MVPA	2	(Not reported)	0

Domingues 2022 (41)	Night	Sleep period<br>Total sleep time<br>Sleep duration	(Not reported)	(Not reported)	2	24 h	4	1	Sedentary behavior<br>Sleep (unspecified)<br>Light PA<br>MVPA	2	The absence of muscle mass evaluation and the recording of the menstrual cycle in girls may interfere in the metabolism and sleep health; Sleep environment characteristics could be used as confounding factors in the analyses if they were assessed	2
Dumuid 2017 (42)	Night	Sleep period<br>Total sleep time<br>Sleep duration	(Not reported)	(Not reported)	0	(Not reported)	5	1	Sedentary behavior<br>Sleep duration<br>Light PA<br>Moderate PA<br>Vigorous PA	0	Activity was measured using waist-worn accelerometers, which have been reported to cause the overestimation of sleep duration	2
Dumuid 2018 (43)	24-hour/daily cycle	Bedtime<br>Wake time<br>Total sleep time<br>Nap frequency<br>Nap duration	(Not reported)	(Not reported)	2	100%	4	1	Sedentary time<br>Sleep (unspecified)<br>Light PA<br>MVPA	2	With accelerometers sleep duration must be estimated, based on the accelerometry movement trace and/or in combination with a sleep self-report diary	2
Dumuid 2018 (44)	Night	Total sleep time	(Not reported)	(Not reported)	2	24 h	4	1	Sedentary time<br>Sleep (unspecified)<br>Light PA<br>MVPA	2	(Not reported)	0
Dumuid 2018 (45)	Night	Sleep period	(Not reported)	(Not reported)	2	24 h	4	1	Sedentary time<br>Sleep (unspecified)<br>Light PA<br>MVPA	2	(Not reported)	0
Dumuid 2019 (46)	24-hour/daily cycle	Bedtime<br>Wake time<br>Total sleep time	(Not reported)	(Not reported)	2	24 h	4	1	Sedentary time<br>Sleep duration<br>Light PA<br>MVPA	2	(Not reported)	0
Dumuid 2020 (47)	Night	Bedtime<br>Wake time<br>Sleep duration	(Not reported)	(Not reported)	2	24 h	4	1	Sedentary time<br>Sleep (unspecified)<br>Light PA<br>MVPA	2	(Not reported)	0

Dumuid 2021 (48)	Unclear	Bedtime<br>Wake time<br>Total sleep time<br>Sleep duration	(Not reported)	(Not reported)	2	24 h	4	1	Sedentary time<br>Sleep (unspecified)<br>Light PA<br>MVPA	2	(Not reported)	0
Dumuid 2022 (49)	24-hour/daily cycle	Sleep unspecified	(Not reported)	(Not reported)	2	24 h	4	1	Sedentary behavior<br>Sleep (unspecified)<br>Light PA<br>MVPA	2	(Not reported)	0
Dumuid 2022 (50)	24-hour/daily cycle	Bedtime<br>Wake time<br>Time in bed<br>Sleep period<br>Total sleep time<br>Total wake time	(Not reported)	(Not reported)	2	24 h	4	1	Sedentary time<br>Sleep (unspecified)<br>Light PA<br>MVPA	2	(Not reported)	0
Fairclough 2017 (51)	Night	Sleep (unspecified)	(Not reported)	(Not reported)	2	24 h	4	1	Sedentary time<br>Sleep duration<br>Light PA<br>MVPA	2	Sleep time was based on sustained inactivity bouts within a predefined angular range of arm motion rather than using polysomnography	2
Fairclough 2021 (52)	Night	Sleep duration	(Not reported)	(Not reported)	2	24 h	4	1	Sedentary time<br>Sleep duration<br>Light PA<br>MVPA	2	(Not reported)	0
Fairclough 2023 (53)	24-hour/daily cycle	Bedtime<br>Wake time<br>Time in bed<br>Total sleep time<br>Waking frequency<br>Sleep duration	(Not reported)	(Not reported)	2	24 h	5	1	Sedentary time<br>Sleep duration<br>Light PA<br>Moderate PA<br>Vigorous PA	2	(Not reported)	0

Gupta 2020 (54)	24-hour/daily cycle	Time in bed	Time in bed used as a proxy of sleep time	Midnight to midnight	6	24 h	9	1	Time in bed<br>Work sedentary behavior<br>Work light PA<br>Work MVPA<br>Work standing<br>Leisure sedentary behavior<br>Leisure light PA<br>Leisure MVPA<br>Leisure standing	2	(Not reported)	0
Healy 2021 (55)	Night	Sleep duration	Total sleep time = minutes spent asleep each night	(Not reported)	4	24 h	4	1	Sedentary behavior<br>Sleep duration<br>Light PA<br>MVPA	2	(Not reported)	0
Hofman 2022 (56)	Night	Bedtime<br>Wake time<br>Sleep duration	(Not reported)	(Not reported)	2	24 h	4	1	Sedentary behavior<br>Sleep duration<br>Light PA<br>MVPA	2	Method only provides an estimate of sleep, which might be hard to distinguish from sedentary behaviour; Sample included only 28 participants with a sleep duration 9+ hours and potentially U-shaped relationship between sleep duration and mental health could not be explored	2
Janda 2024 (57)	Unclear	Wake time<br>Sleep onset<br>Sleep unspecified	Sleep = difference between the sleep onset and waking time	Midnight to midnight	6	24 h	4	1	Sedentary behavior<br>Sleep (unspecified)<br>Light PA<br>MVPA	2	(Not reported)	1
Johansson 2020 (58)	24-hour/daily cycle	Bedtime<br>Time in bed<br>Get up time	Time in bed = difference between bedtime and get up time	(Not reported)	4	24 h	6	1	Sedentary behavior<br>Standing<br>Stepping<br>Moving<br>Time in bed<br>HIPA (sum of climbing stairs, running, cycling, and rowing)	2	Time in bed is only a proxy and not a valid measure of sleep duration or sleep quality, so did not quantify the measure of association between time in bed and the outcomes	2

Kim 2021 (59)	Unclear	Total sleep time	(Not reported)	(Not reported)	2	24 h	4	1	Sedentary time<br>Sleep duration<br>Light PA<br>MVPA	2	(Not reported)	0
Kuzik 2020 (60)	24-hour/daily cycle	Total sleep time<br>Nap duration	(Not reported)	(Not reported)	2	24 h	4	1	Light PA<br>MVPA<br>Total sleep<br>Stationary time	2	(Not reported)	0
Le 2022 (61)	Night	Time in bed<br>Sleep onset<br>Sleep offset<br>Total sleep time<br>WASO<br>SOL<br>Sleep duration	Hours of sleep duration and average time awake were calculated	(Not reported)	3	24 h	5	2	Sedentary behavior<br>Sleep duration<br>WASO<br>Light PA<br>MVPA	2	Accelerometry estimates of sleep often show low specificity, which might result in overestimation of sleep duration and underestimation of time awake in bed	2
Lee 2020 (62)	24-hour/daily cycle	Total sleep time<br>SOL<br>WASO<br>Fragmentation index	(Not reported)	(Not reported)	2	24 h	4	1	Sedentary time<br>Sleep duration<br>Light PA<br>MVPA	2	Actigraphy-based estimates provide indirect estimates of sleep	2
Lu 2024 (63)	24-hour/daily cycle	Sleep duration	(Not reported)	(Not reported)	0	(Not reported)	4	1	Sedentary behavior<br>Sleep duration<br>Light PA<br>MVPA	0	(Not reported)	0
Marmol-Perez 2024 (64)	24-hour/daily cycle	Bedtime<br>Wake time<br>Sleep duration	(Not reported)	(Not reported)	0	(Not reported)	4	1	Sedentary behavior<br>Sleep (unspecified)<br>Light PA<br>MVPA	0	(Not reported)	0
Marshall 2022 (65)	24-hour/daily cycle	Total sleep time	(Not reported)	(Not reported)	0	(Not reported)	4	1	Sedentary time<br>Sleep (unspecified)<br>Light PA<br>MVPA	0	(Not reported)	0

Matricciani 2021 (66)	Night	Sleep period<br>Sleep midpoin<br>Day-to-day variability<br>Sleep efficiency	Sleep period = difference between sleep onset and offset; sleep timing = midpoint between sleep onset and offset; day-to-day sleep variability = coefficient of variation of sleep period; sleep efficiency = percent of minutes scored as sleep between onset and offset	(Not reported)	4	24 h	4	1	Sedentary time<br>Sleep duration<br>Light PA<br>MVPA	2	No studies that have validated the monitor against PSG in children; The Sadeh algorithm was applied to score sleep onset and offset, for both adults and children	2
Mellow 2022 (67)	24-hour/daily cycle	Total sleep time	(Not reported)	(Not reported)	2	24 h	4	1	Sedentary behavior<br>Sleep (unspecified)<br>Light PA<br>MVPA	2	Most of the sample had good sleep quality, or that the single-item measure did not capture the true variability in sleep quality; Future studies should consider exploring additional measures such as sleep stage duration, sleep efficiency, and spindle activity	2
Mellow 2024 (68)	Unclear	Wake time<br>Sleep onset<br>Sleep duration	(Not reported)	(Not reported)	2	24 h	4	1	Sedentary behavior<br>Sleep duration<br>Light PA<br>MVPA	2	Non-wear time is not typically accrued during sleep, and thus increasing time in sleep as a result of the proportional rescaling across all time-use components may have resulted in the overestimation of time spent in this behavior	2
Migueles 2020 (69)	Unclear	Bedtime<br>Wake time<br>Total sleep time	(Not reported)	(Not reported)	0	(Not reported)	4	1	Sedentary time<br>Light PA<br>MVPA<br>Sleep period	0	(Not reported)	0

Migueles 2023 (70)	Night	Sleep period<br>Sleep unspecified	(Not reported)	(Not reported)	2	Average day duration	4	1	Sedentary behavior<br>Sleep (unspecified)<br>Light PA<br>MVPA	2	Algorithm used to estimate sleep was validated only in adults	2
Mitchell 2023 (71)	24-hour/daily cycle	Sleep period	Sleep time = longest reclining bout between noon and noon each day (at least 2 hours) or any bouts lasting at least 5 hours	(Not reported)	2	(Not reported)	4	1	Sedentary behavior<br>Sleep (unspecified)<br>Light PA<br>MVPA	0	Accelerometers can only detect time spent in bed and cannot capture biological sleep; Differentiating between positive and negative sedentary behavior, and sleep duration and sleep quality are further avenues for exploration	2
Murphy 2024 (72)	24-hour/daily cycle	Sleep onset<br>Sleep offset<br>Sleep period	Sleep period time = difference between sleep onset and offset; sleep efficiency = percent of time asleep during sleep periods	(Not reported)	4	24 h	4	1	Sedentary time<br>Sleep duration<br>Light PA<br>MVPA	2	Compositional data analysis considers sleep as a component of time but may also need to account for sleep as a multidimensional construct involving sleep quality	2
Ng 2021 (73)	Unclear	Sleep onset<br>Sleep offset<br>WASO	(Not reported)	(Not reported)	2	24 h	4	1	Sedentary time<br>Sleep (unspecified)<br>Light PA<br>MVPA	2	(Not reported)	0
Niemelä 2024 (74)	Night	Bedtime<br>Wake time<br>Time in bed<br>Total sleep time<br>WASO<br>Sleep efficiency	Sleep period = longest sleep period from the detected start of bedtime to the end of bedtime	(Not reported)	4	24 h	4	1	Sedentary time<br>Sleep (unspecified)<br>Light PA<br>MVPA	2	The device has shown limited specificity in detecting wake periods during sleep which can indicate lower accuracy of sleep continuity metrics, including sleep efficiency	2
Oviedo-Caro 2020 (75)	24-hour/daily cycle	Sleep unspecified	Sleep component represents all sleep occurring between 12 PM and 12 PM	(Not reported)	2	(Not reported)	4	1	Sedentary time<br>Sleep (unspecified)<br>Light PA<br>MVPA	0	(Not reported)	0

Powell 2020 (76)	Night	Rise time<br>Total sleep time	Sleep = [time between 0500 h and the first non-sedentary epoch]+[time between the last non-sedentary epoch and the following 0500 h time point]	(Not reported)	2	24 h	5	1	Sedentary time<br>Sleep (unspecified)<br>Light PA<br>MVPA<br>Standing	2	Surrogate measure of sleep (bedtime) was used; A participant was presumed to be asleep based on a lack of movement during a set time-period, which may not be accurate since the method would not detect activities such as screen time or reading in bed	2
Rasmussen 2023 (77)	Night	Wake time<br>Sleep onset<br>Sleep duration	Sleep duration = difference between sleep onset and waking up time	(Not reported)	4	24 h	4	1	Sedentary behavior<br>Sleep duration<br>Light PA<br>MVPA	2	(Not reported)	0
Rorem 2024 (78)	24-hour/daily cycle	Total sleep time	(Not reported)	(Not reported)	2	24 h	5	1	Sleep duration<br>Light PA<br>MVPA<br>Non-screen-time sedentary time<br>Screen time	2	(Not reported)	0
Runacres 2023 (79)	Night	Bedtime<br>Wake time<br>Total sleep time<br>Sleep efficiency	(Not reported)	(Not reported)	0	(Not reported)	5	1	Sedentary time<br>Sleep (unspecified)<br>Light PA<br>Moderate PA<br>Vigorous PA	0	(Not reported)	0
Sandborg 2022 (80)	Unclear	Bedtime<br>Wake time<br>Sleep period	(Not reported)	(Not reported)	0	(Not reported)	4	1	Sedentary behavior<br>Sleep (unspecified)<br>Light PA<br>MVPA	0	(Not reported)	0
Smith 2021 (81)	24-hour/daily cycle	Total sleep time	(Not reported)	(Not reported)	0	(Not reported)	3 and 4	1	Sedentary behavior<br>Sleep (unspecified)<br>Light PA<br>MVPA	0	(Not reported)	0
Smith 2021 (82)	Unclear	Sleep onset<br>Sleep offset<br>Total sleep time	(Not reported)	(Not reported)	0	(Not reported)	4	1	Sedentary behavior<br>Sleep (unspecified)<br>Light PA<br>MVPA	0	(Not reported)	0

Song 2018 (83)	Night	Sleep duration	Actual sleep minutes between time in bed and time out of be	(Not reported)	2	(Not reported)	4	1	Sedentary behavior<br>Sleep duration<br>Light PA<br>Moderate PA	0	(Not reported)	0
St Laurent 2023 (84)	24-hour/daily cycle	Night/nap sleep onset<br>Night/nap sleep offset<br>Night/nap sleep time<br>Night/nap WASO<br>Night/nap SOL	Prliminary models: Sleep time = subtracting wake bouts WASO and SOL from the rest intervals; Compositional models: Proxy of sleep time = time in bed (sum of time spent in rest intervals)	Wake onset for day x until wake onset for day x+1	6	24 h	4	1	Sedentary time<br>Sleep duration<br>Light PA<br>MVPA	2	There was possibililty for misclassification of behaviors given different data configuration and processing protocols, and that the waist is generally preferable for physical activity metrics, whereas the wrist is recommended for sleep; Sleep time in the compositional models did not exclude wake bouts while in bed	2
Swindell 2020 (85)	Night	Total sleep time	(Not reported)	(Not reported)	2	24 h	4	1	Sedentary time<br>Sleep (unspecified)<br>Light PA<br>MVPA	2	(Not reported)	0
Talarico 2018 (86)	Night	Sleep onset<br>Sleep offset<br>Sleep duration	(Not reported)	(Not reported)	2	24 h	4	1	Sedentary behavior<br>Sleep duration<br>Light PA<br>MVPA	2	(Not reported)	0
Tan 2023 (87)	24-hour/daily cycle	Total sleep time	Sleep = sum of night sleep and naps, defined as periods exceeding 15 min of sustained inactivity during wear time	(Not reported)	2	(Not reported)	4	1	Sleep (unspecified)<br>Light PA<br>MVPA<br>Inactivity	0	Other dimensions of sleep could not be distinguished; In classification of the movement behaviors in the present study was algorithm-driven, which could not always differentiate sleep-related behaviors that were neither sleep nor inactivity	2

Taylor 2018 (88)	24-hour/daily cycle	Sleep onset<br>Sleep offset<br>Waking duration<br>Nap duration	(Not reported)	(Not reported)	2	24 h	5	2	Sedentary time<br>Sleep duration<br>Light PA<br>MVPA<br>WASO	2	The length of each day varies both within and between participants, requiring all data to be scaled to 24 h periods	2
Taylor 2020 (89)	Night	Sleep onset<br>Sleep offset<br>Waking duration<br>Sleep duration	Sleep duration = difference between sleep onset (time go to sleep) and sleep offset (time wake in morning), after excluding WASO	Wake time on day 1 to wake time on day 2 (and so on)	6	24 h	5	2	Sedentary time<br>Sleep duration<br>WASO<br>Light PA<br>MVPA	2	(Not reported)	0
Taylor 2023 (90)	24-hour/daily cycle	Sleep onset<br>Sleep offset<br>Total sleep time<br>WASO	Total sleep time = difference between sleep onset and offset, excluding WASO	Wake time on day 1 to wake time on day 2 (and so on)	6	24 h	3 (3.5 yrs)<br>4 (5 yrs)	1	Sedentary behavior<br>Sleep duration<br>Light PA<br>MVPA	2	(Not reported)	0
Tyler 2022 (91)	24-hour/daily cycle	Sleep duration	(Not reported)	(Not reported)	2	24 h	4	1	Sedentary time<br>Sleep (unspecified)<br>Light PA<br>MVPA	2	(Not reported)	0
Vanderlinden 2023 (92)	24-hour/daily cycle	Total sleep time	(Not reported)	(Not reported)	2	24 h	4	1	Sedentary behavior<br>Light PA<br>MVPA<br>Time spent in sleep	2	(Not reported)	0
Verhoog 2020 (93)	Night	Sleep duration	(Not reported)	(Not reported)	2	24 h	4	1	Sedentary behavior<br>Sleep duration<br>Light PA<br>MVPA	2	Only overnight sleep periods were included from the sleep diary, so naps during the day were included as sedentary behavior	2

Walmsley 2022 (94)	24-hour/daily cycle	Unclear	Sleep = non-waking behavior	(Not reported)	1	(Not reported)	4	1	Sedentary behavior<br>Sleep (unspecified)<br>Light PA<br>MVPA	0	Although comparable with UK estimates from other sources, sleep measurements should be interpreted cautiously: 'ground truth' labels for sleep came from a time use diary, which may identify time in bed rather than physiological sleep. Sleep measurements require validation using PSG	2
