# Supplementary material for: Characterizing device-measured sleep in observational health research using compositional data analysis: a systematic review
Source: Int J Behav Nutr Phys Act. 2026 Jun 15;23:78. doi: 10.1186/s12966-026-01935-8 (PMC13501660; doi:10.1186/s12966-026-01935-8)
Supplement: Supplementary file 7 [file 12966_2026_1935_MOESM7_ESM.pdf]

Additional File 7. Funding and conflict of interest disclosures of included articles.

Article	Funding Sources Reported	Authors' Description of Funding Source(s)	COI Reported	Authors' Description of COI	Study Data Availability	Authors' Description of Study Data Availability
Balbim 2024 (25)	Yes	Funding for this work was provided to T.L.A. by the Jack Brown and Family Alzheimer Research Foundation, Dawn Shaw, Vancouver Coastal Health Research Institute Innovation and Translational Research Awards (F16-01717), Canadian Institutes of Health Research (AWD- 016347; MOP-142206; PJT-148902), and Heart and Stroke Foundation of Canada (G-15-0009019). G.M.B. and N.C.B.S.S. are jointly funded by the Canadian Institutes of Health Research and Michael Smith Health Research BC. N.C.B.S.S. is supported by the Canadians for Leading Edge Alzheimer's Research. R.S.F. is funded by the Michael Smith Health Research BC. T.L.A. is a Canada Research Chair (Tier 1) in Healthy Aging. The funding sources had no role in the study design, in the collection, analysis, and interpretation of data, in the writing of the report, and in the decision to submit the article for publication.	Yes	None	Available upon request	Data will be made available on request.
Bianchim 2022 (26)	Yes	This work was supported by the Cystic Fibrosis Trust UK under its programme grant for Strategic Research Centres (grant reference number RP-PG-0108-10011). M.S.B. was funded by the Cystic Fibrosis Trust. The funder had no role in the conduct of this study, the writing of the manuscript, or the decision to submit it for publication.	Yes	M.S.B., K.A.M., M.A.M., C.A.W., S.D. and A.R.B. declare funding from the Cystic Fibrosis Trust UK. All other authors declare that they have no competing interests and the funders had no role in the design of the study; in the collection, analyses, or interpretation of data; in the writing of the manuscript, or in the decision to publish the results.	Available upon request	The datasets generated and/or analysed during the current study are not publicly available due to GDPR regulations and to protect individual privacy but are available from the corresponding author on reasonable request.
Biddle 2018 (27)	Yes	This research was funded by NIHR Collaborations for Leadership in Applied Health Research and Care (CLAHRC) East Midlands and supported by the NIHR Leicester Biomedical Research Centre.	Yes	The authors declare no conflict of interest.	No	

Blodgett 2023 (28)	Yes	JB is supported through a British Heart Foundation grant (SP/F/20/ 150002). JJM is supported through a Medical Research Council grant (MR/N013867/1). ES is supported by a National Health and Medical Research Council (NHMRC, Australia) Investigator Grant Leadership Level 2 (APP1194510). The views expressed are those of the author(s) and not necessarily those of study funders.	Yes	The authors declare that they have no competing interests.	Yes	The datasets supporting this article are available in the UK Data Service repository [1970 British Cohort Study: https://beta.ukdataservice.ac.uk/datacatalogue/series/series?id=200001].
Blodgett 2024 (29)	Yes	This project was funded by a British Heart Foundation Special Grant (SP/F/20/150002) and National Health and Medical Research Council (Australia) Investigator (APP1194510) and Ideas (APP1180812) Grants. The establishment of the ProPASS consortium was supported by an unrestricted 2018-20 grant by PAL Technologies (Glasgow, UK). ActiPASS development was partly funded by FORTE, Swedish Research Council for Health, Working Life and Welfare (2021-01561). E.S. is funded by a National Health and Medical Research Council Investigator Grant (APP1194510). G.M. is supported by a National Health and Medical Research Council Principal Research Fellowship (APP1121844). A.D.H. receives support from the British Heart Foundation, the Horizon 2020 Framework Programme of the European Union, the National Institute for Health Research University College London Hospitals Biomedical Research Centre, the UK Medical Research Council, the National Institute for Health Research, and the Wellcome Trust and works in a unit that receives support from the UK Medical Research Council.	Yes	All authors declare no disclosure of interest for this contribution.	No	Access to data is not available directly from the authors of this manuscript. Access to cohort data may be available by contacting individual cohort and following their specific governance and access requirements.

Brakenridge 2021 (30)	Yes	We are grateful for the funding support from the National Health and Medical Research Council (NHMRC grants 233200 and 1007544), Australian Government Department of Health and Ageing, Abbott Australasia Pty Ltd., Alphapharm Pty Ltd., Amgen Australia, AstraZeneca, Bristol-Myers Squibb, City Health Centre-Diabetes Service-Canberra, Department of Health and Community Services-Northern Territory, Department of Health and Human Services-Tasmania, Department of Health-New South Wales, Department of Health-Western Australia, Department of Health-South Australia, Department of Human Services-Victoria, Diabetes Australia, Diabetes Australia Northern Territory, Eli Lilly Australia, Estate of the Late Edward Wilson, GlaxoSmithKline, Jack Brockhoff Foundation, Janssen-Cilag, Kidney Health Australia, Marian & FH Flack Trust, Menzies Research Institute, Merck Sharp & Dohme, Novartis Pharmaceuticals, Novo Nordisk Pharmaceuticals, Pfizer Pty Ltd., Pratt Foundation, Queensland Health, Roche Diagnostics Australia, Royal Prince Alfred Hospital, Sydney, Sanofi Aventis, sanofi-synthelabo, and the Victorian Government's OIS Program. DD, GH, NO are supported by the NHMRC Fellowships scheme. Funding for JB came from a program project (P01 AG052352) supported by the US National Institute on Aging. The funders of this study had no role in the data analysis or interpretation of the results.	Yes	No relevant conflicts of interest to disclose	Available upon request	Datasets are available on request to corresponding author.

Brakenridge 2024 (31)	Yes	Funding Open Access funding enabled and organized by CAUL and its Member Institutions.The Maastricht Study was supported by the European Regional Development Fund via OP-Zuid, the Province of Limburg, the Dutch Ministry of Economic Affairs (grant 31O.041), Stichting De Weijerhorst (Maastricht, the Netherlands), the Pearl String Initiative Diabetes (Amsterdam, the Netherlands), the Cardiovascular Center (CVC, Maastricht, the Netherlands), CARIM School for Cardiovascular Diseases (Maastricht, the Netherlands), CAPHRI Care and Public Health Research Institute (Maastricht, the Netherlands), NUTRIM School for Nutrition and Translational Research in Metabolism (Maastricht, the Netherlands), Stichting Annadal (Maastricht, the Netherlands), Health Foundation Limburg (Maastricht, the Netherlands), and by unrestricted grants from Janssen-Cilag BV (Tilburg, the Netherlands), Novo Nordisk Farma BV (Alphen aan den Rijn, the Netherlands), and Sanofi-Aventis Netherlands BV (Gouda, the Netherlands). DD was funded by an Australian Research Council (ARC) Discovery Early Career Award (DE230101174). DWD and NO are supported by the NHMRC Fellowships scheme and the Victorian Government's OIS Program. GNH was supported by an MRFF-NHMRC Emerging Leadership Investigator Grant (no. 1193815). The funders of this study and The Maastricht Study were not involved in the design of the study; the collection, analysis, and interpretation of data; writing the report; and did not impose any restrictions regarding the publication of the report.	Yes	BEdG is a member of the editorial board of Diabetologia. The remaining authors declare that there are no relationships or activities that might bias, or be perceived to bias, their work.	Available upon request	Datasets generated and analysed are not publicly available but are available from the corresponding author upon reasonable request.
Brayton 2023 (32)	Yes	Research reported in this publication was supported by Children's Health. The content is solely the responsibility of the authors and does not necessarily represent the official views of Children's Health. The funders had no role in study design, data collection and analysis,	Yes	No potential conflict of interest was reported by the author(s).	No	

		decision to publish, or preparation of this abstract.				
Cabanas-Sánchez 2021 (33)	Yes	This work was supported by FIS grants 16/609 and 19/319 (State Secretary of R+D+I and FEDER/FSE), MINECO R+D+I grant (DEP2013-47786-R), the SALAMANDER project (PCIN 2016-145), Biomedical Research Networking Center on Frailty and Healthy Aging (CIBERFES) and FEDER funds from the European Union (CB16/10/00477). VC-S is supported by the Spanish Ministry of Science, Innovation and Universities (IJC2018-038008-I). IE-C is supported by the Spanish Ministry of Economy and Competitiveness (RTI2018-095284-J-100). DM-G is supported by a'Ramon y Cajal' contract (RYC-2016-20546). The funders played no role in the study design, the data collection or analysis, the decision to publish, or the preparation of the manuscript.	Yes	The authors declare none competing interest.	Available upon request	The datasets used and/or analyzed during the current study may be available from the Principal Investigator of the project (FR-A) upon reasonable request.
Chen 2023 (34)	Yes	This study was supported by the National Social Science Fund of China (No. 22TYB00517) and Shanghai Sport Science and Technology Project of Shanghai Administration of Sports (No. 22Q005).	Yes	The author(s) declared no potential conflicts of interest.	Available upon request	The dataset analyzed during the current study are available from the corresponding author on reasonable request.
Chong 2021 (35)	Yes	This research was funded by the University of Wollongong under the 2017 Faculty Seed Grant programme. K.H.C. is supported by a PhD scholarship from the University of Wollongong. D.D. is supported by the Australian National Health and Medical Research Council (APP1162166) and the Heart Foundation (102084). A.D.O. is supported by a National Health and Medical Research Council of Australia Investigator Grant (APP1176858). The funders had no role in the design of the study; in the collection, analyses or interpretation of data; in the writing of the manuscript, or in the decision to publish the results.	Yes	The authors declare no conflict of interest.	No	The data presented in this study are not openly available as participants did not provide informed consent for data sharing.

Collings 2023 (36)	Yes	The ORISCAV-LUX 2 study was funded by the Luxembourg Institute of Health. We have no declaration to make regarding the role of the funding body in the design of the study, the collection, analysis, interpretation of data, the writing of the report, or the decision to submit the article for publication. The views expressed are those of the authors and not necessarily those of the funder.	Yes	The authors declare that they have no known competing financial interests or personal relationships that could have appeared to influence the work reported in this paper.	Available upon request	Deidentified data supporting the conclusions of this article may be available upon reasonable request, if consent is provided by all authors and the ORISCAV-LUX study group. Requests to access the data should be directed to LM.
Collings 2023 (37)	Yes	The ORISCAV-LUX 2 study was funded by the Luxembourg Institute of Health. We have no declaration to make regarding the role of the funding body in the design of the study, the collection, analysis, and interpretation of data, and in writing the manuscript. The views expressed are those of the authors and not necessarily those of the funder.	Yes	The authors declare that they have no competing interests.	Available upon request	Deidentified data supporting the conclusions of this article may be available upon reasonable request, if consent is provided by all authors and the ORISCAV-LUX study group. Requests to access the data should be directed to LM.
Curtis 2020 (38)	Yes	This study used data from a larger project funded by an Australian National Health and Medical Research Council (NHMRC) Project Grant (APP1080186). R.G.C. and J.R. were also supported by the NHMRC Project Grant. C.M. was supported by a NHMRC Career Development Fellowship (APP1125913). C.V. was supported by a Future Leader Fellowship from the National Heart Foundation of Australia (ID 100427). S.E. and D.D. were supported by an Australian Government Research Training Program Scholarship. The funding bodies had no role in the design, data collection, analysis, or interpretation of this study.	Yes	The authors declare no conflicts of interest.	No	
Curtis 2023 (39)	Yes	This project is funded by the Australian National Health and Medical Research Council (APP1163338). The funding body was not involved in the study design, data collection, interpretation of data, data analysis, or in the preparation of the manuscript. CM receives an Investigator Grant from the Medical Research Future Fund (1193862). DD receives an Early Career Research Fellowship from the National Health and Medical Research Council (1162166).	Yes	The authors have declared that they have no competing interests.	Available upon request	The datasets analysed during the current study are available from the corresponding author on reasonable request.

deFaria 2022 (40)	Yes	FRF received a scholarship grant from the Coordination for the Improvement of Higher Education Personnel. Scholarship grant number 88881.190375/2018-01. The funders had no role in study design, data collection and analysis, decision to publish, or preparation of the manuscript.	Yes	The authors have declared that no competing interests exist.	Yes	The dataset is available at the Mendeley repository: https://data.mendeley.com/datasets/2gyv6hd898/1.
Domingues 2022 (41)	Yes	SFD The Coordination of the Improvement of Higher Education Personnel. This study was financed in part by the Coordenação de Aperfeicoamento de Pessoal de Nível Superior - Brasil (CAPES) - Finance Code 001. The funders had no role in study design, data collection and analysis, decision to publish, or preparation of the manucript.	Yes	The authors have declared that no competing interests exist.	Yes	All relevant data are within the paper and its Supporting Information files.
Dumuid 2017 (42)	Yes	Dorothea Dumuid is supported by an Australian Government Research Training Program Scholarship. Martín-Fernández is partially supported by the Spanish Ministry of Economic Competitiveness under the project CODA-RETOS (MTM2015-65016-C2-1(2)-R). Carol Maher is the recipient of a postdoctoral fellowship award from the Australian National Heart Foundation (100188). ISCOLE was funded by The Coca-Cola Company. The funders had no role in study design, data collection and analysis, decision to publish, or preparation of this article.	Yes	The authors declared no potential conflicts of interest with respect to the research, authorship, and/or publication of this article.	No	
Dumuid 2018 (43)	Yes	D Dumuid is supported by an Australian Government Research Training scholarship. C Maher is supported by an Australian National Health and Medical Research Council Career Development Fellowship.	Yes	The authors declare that they have no conflict of interest	Available upon request	There are no linked research data sets for this paper. Data will be made available on request.
Dumuid 2018 (44)	Yes	This work was supported by an Australian Government Research Training Program Scholarship [DD], the National Heart Foundation (100188) [CM] and partially supported by the Spanish Ministry of Economy and Competitiveness under the project CODA-RETOS (MTM2015-65016- C2-1(2)-R) [JAMF]. The International Study of Childhood Obesity, Lifestyle and Environment (ISCOLE) was funded	Yes	The authors declare that they have no conflict of interest.	Available upon request	The data that support the findings of this study are available from Peter T. Katzmarzyk (Peter.Katzmarzyk@pbrc.edu) but restrictions apply to the availability of these data, which were used under license for the current study, and so are not publicly available. Data are however available from the authors upon reasonable request and with permission of

		by The Coca-Cola Company. The funders had no role in the design and conduct of the study, data collection, management, analysis and interpretation of the data; and decision to publish, preparation, review or approval of this manuscript.				Pennington Biomedical Research Center.
Dumuid 2018 (45)	Yes	This work was supported by an Australian Government Research Training Program Scholarship [DD] and by The National Heart Foundation (100188) [CM]. This research has been partially supported by the Spanish Ministry of Economy and Competitiveness under the project CODA-RETOS (MTM2015-65016- C2-1(2)-R) [JAMF]. The ISCOLE study was funded by the Coca-Cola Company. The funders had no role in the design and conduct of the study, data collection, management, ana- lysis and interpretation of the data; and decision to publish, preparation, review or approval of this manuscript. DD, TES, ZP and JAMF are not part of ISCOLE group.	Yes	The authors declare that they have no competing interests.	Available upon request	The data that support the findings of this study are available from Peter T. Katzmarzyk (Peter.Katzmarzyk@pbrc.edu) but restrictions apply to the availability of these data, which were used under license for the current study, and so are not publicly available. Data are however available from the authors upon reasonable request and with permission of Pennington Biomedical Research Center.

Dumuid 2019 (46)	Yes	The Child Health CheckPoint was supported by the National Health and Medical Research Council (NHMRC) of Australia (Project Grants 1041352, 1109355), The Royal Children's Hospital Foundation (2014-241), the Murdoch Children's Research Institute (MCRI), The University of Melbourne, the National Heart Foundation of Australia (100660), and the Financial Markets Foundation for Children (2014-055, 2016-310). D.D. was supported by the Australian Government Research Training Program. M.W. was supported by NHMRC Senior Research Fellowship 1046518 and Cure Kids New Zealand. F.M. was supported by NHMRC Career Development Fellowship 1111160. D.B. was supported by NHMRC Senior Research Fellowship 1064629 and National Heart Foundation of Australia Honorary Future Leader Fellowship 100369. The MCRI administered the research grants for the study and provided infrastructural support (IT and biospecimen management) to its staff and the study, but played no role in the conduct or analysis of the trial. The Department of Social Services played a role in study design; however, no other funding bodies had a role in the study design and conduct; data collection, management, analysis, and interpretation; preparation, review, or approval of the manuscript; and decision to submit the manuscript for publication. Research at the MCRI is supported by the Victorian Government's Operational Infrastructure Support Program.	Yes	The authors declare no conflicts of interest.	No	

Dumuid 2020 (47)	Yes	DD is supported by the National Health and Medical Research Council (NHMRC) (Early Career Fellowship 1162166) and the National Heart Foundation of Australia (102084). MW is supported by the NHMRC (Principal Research Fellowship 1160906). DB is supported by an NHMRC Investigator Grant (1175744). FW is supported by the NHMRC (Early Career Fellowship 1158661). The Child Health CheckPoint was supported by the NHMRC (Project Grants 1041352, 1109355), The Royal Children's Hospital Foundation (2014-241), the Murdoch Children's Research Institute (MCRI), The University of Melbourne, the National Heart Foundation of Australia (100660), and the Financial Markets Foundation for Children (2014-055, 2016-310). Research at the MCRI is supported by the Victorian Government's Operational Infrastructure Support Program.	Yes	All authors state that they have no conflicts of interest.	No	
Dumuid 2021 (48)	Yes	DD was supported by the National Health and Medical Research Council (NHMRC) [Early Career Fellowship 1162166] and the National Heart Foundation of Australia [Postgraduate Fellowship 102084]. MW was supported by the NHMRC [Principal Research Fellowship 1160906]. DB was supported by an NHMRC Investigator Grant [1175744]. ADO was supported by an NHMRC Leadership Fellowship [1176858]. The Child Health CheckPoint was supported by the NHMRC of Australia [Project Grants 1041352, 1109355], The Royal Children's Hospital Foundation [2014-241], the Murdoch Children's Research Institute (MCRI), The University of Melbourne, the National Heart Foundation of Australia [100660] and the Financial Markets Foundation for Children [2014-055, 2016-310]. Research at the MCRI is supported by the Victorian Government's Operational Infrastructure Support Program. The funders had no role in study design, data collection and analysis, decision to publish, or preparation of the manuscript.	Yes	The authors have declared that no competing interests exist.	Available upon request	We are not the owners of the CheckPoint and LSAC data. There are legal restrictions on public sharing of the data. To become a licensed data user for the CheckPoint and LSAC studies, researchers are required to sign a confidentiality deed, found here: https://ada.edu.au/confidentiality-deed-poll-november-2019/. The confidentiality deed imposes a legal obligation to not share any unit-level data with anyone unless they also are registered data users. Researchers can apply to become registered data users by following the link provided: https://dataverse.ada.edu.au/dataverse/ncld. All the data used in our study can be downloaded at this website, except we used a slightly different form of accelerometry data. The accelerometry data currently available in the CheckPoint dataset have all minutes when the accelerometers were removed for sport classified as MVPA (moderate-to-vigorous physical activity). For our study,

						we requested CheckPoint to provide us accelerometry data where the sport non-wear time was replaced with 50% MVPA, 30% light physical activity and 20% sedentary time, as we believe this better reflects children's activity levels during sport. The alternate accelerometry files are available to registered users, upon request to the CheckPoint team at LSAC. ChildHealthCheckPoint@mcri.edu.au.
Dumuid 2022 (49)	Yes	DD is supported by the Australian National Health and Medical Research Council (NHMRC) Early Career Fellowship APP1162166. MLM is supported by a Dementia Australia Research Foundation PhD scholarship.	Yes	Nothing to disclose.	No	
Dumuid 2022 (50)	Yes	This work was funded by the Australian National Health and Medical Research Council (NHMRC) (GNT1041352; GNT1109355; GNT1186123; GNT1162166 to DD; GNT1091124 to KLy; GNT1175744 to DPB; GNT1160906 to MW); the National Heart Foundation of Australia (100660; 102084 to DD; 101239 to KLy; The Royal Children's Hospital Foundation (2014-241); the Murdoch Children's Research Institute (MCRI) (No award number available); The University of Melbourne (No award number available); the Financial Markets Foundation for Children (2014-055, 2016-310); and the Australian Department of Social Services (DSS) (No award number available). Research at the MCRI is supported by the Victorian Government's Operational Infrastructure Support Program (No award number available). DD and TO are researchers in the NHMRC Centre of Research Excellence in Driving Global Investment in Global Adolescent Health (GNT1171981).	Yes	None declared.	Available upon request	The Longitudinal Study of Australian Children datasets and technical documents are available to researchers at no cost via a licence agreement. Data access requests are co-ordinated by the National Centre for Longitudinal Data. More information is available at https://dataverse.ada.edu.au/dataverse/lsac.

Fairclough 2017 (51)	Yes	This study was funded by West Lancashire School Sport Partnership, West Lancashire Community Leisure Trust, and Edge Hill University. The funders had no involvement in the study design, data collection, analysis, and interpretation, or writing of the manuscript.	Yes	The authors declare that they have no competing interests.	Yes	All data generated or analysed during this study are included in this published article (and its Additional files 1, 2, 3, 4, 5, 6, 7 and 8).
Fairclough 2021 (52)	Yes	This work was supported by The Waterloo Foundation under Grant #1669- 3509.	Yes	The authors report no conflict of interest.	Available upon request	The datasets used and/or analysed during the current study are available from the corresponding author on reasonable request.
Fairclough 2023 (53)	Yes	Funding was provided by the Waterloo Foundation (#1669/3509; #1669/4195). This funder had no role in the design of the study and collection, analysis, and interpretation of data, and in writing the manuscript.	Yes	The authors declare that they have no competing interests.	Yes	The dataset analysed during the current study is available in the Open Science Framework repository from https://osf.io/2az8b.
Gupta 2020 (54)	Yes	We would like to acknowledge the Danish work environment research fund (journal number 01-2015-03) for financially supporting this study and the Danish government (satspulje) for providing funds to collect DPHACTO cohort data.	Yes	The authors declare that they have no conflict of interest.	No	
Healy 2021 (55)	Yes	Research reported in this publication was supported by an Institutional Development Award (IDeA) Center of Biomedical Research Excellence from the National Institute of General Medical Sciences of the National Institutes of Health under grant number P20GM113125.	No		No	
Hofman 2022 (56)	Yes	The Rotterdam Study is funded by Erasmus Medical Centre and Erasmus University, Rotterdam, Netherlands Organisation for the Health Research and Development (ZonMw), the Research Institute for Diseases in the Elderly (RIDE), the Ministry of Education, Culture and Science, the Ministry for Health, Welfare and Sports, the European Commission (DG XII) and the Municipality of Rotterdam.	Yes	None declared.	Available upon request	Data are available on reasonable request. Requests should be directed towards the management team of the Rotterdam Study (secretariat.epi@erasmusmc.nl), which has a protocol for approving data requests. Because of restrictions based on privacy regulations and informed consent of the participants, data cannot be made freely available in a public repository.

Janda 2024 (57)	Yes	This research was funded by research grants from the Czech Science Foundation (18-09188 S and 22-02392 S) and from the Palacký University Olomouc internal grant (IGA_FTK_2023_001). LA is supported by an Australian Research Council Discovery Early Career Researcher Award (DE220100847). The funders played no role in the design or conduction of the study; in the collection, management, analysis, or interpretation of the data; or in the preparation, review, or approval of the article.	Yes	The authors declare no competing interests.	Yes	The dataset is available at https://doi.org/10.6084/m9.figshare.24131367.v1
Johansson 2020 (58)	Yes	The fifth examination of the CCHS was funded by the Danish Heart Foundation, the Beckett Foundation, the Danish Lung Association, the IMK-Almene Fond, and Helene and Viggo Bruun's Foundation. The funders were not involved in the design and management of the study, in the collection, analysis, or the interpretation of data, in the preparation of the manuscript, or in the decision to submit the manuscript for publication.	Yes	The authors declare that they have no competing interests.	Available upon request	The data generated and analysed during the current study are not publicly available; however, anybody can apply for the use of data by contacting the steering committee of the CCHS [69].
Kim 2021 (59)	Yes	National Institutes of Health grant (R01 HL91024-01A1).	Yes	The authors declare that they have no conflict of interest.	No	
Kuzik 2020 (60)	Yes	The author(s) received no specific funding for this work.	Yes	The authors have declared that no competing interests exist.	Available upon request	Data cannot be shared publicly because of ethical restrictions that only permit study team members to access the data. However, interested researchers may send requests for approval and data access to the University of Alberta Research Ethics Board (contact via reoffice@ualberta.ca).
Le 2022 (61)	Yes	Wiley (1178487) and Bei (1140299) were supported by NHMRC fellowships.	Yes	The authors declare no competing interests.	Yes	This is a secondary analysis of existing data. Materials and measures are available on the Open Science framework for ACES (https://doi.org/10.17605/OSF.IO/H5497), DESTRESS (https://doi.org/10.17605/OSF.IO/QM63W), and SHS

						(https://doi.org/10.17605/OSF.IO/TZ48Y).
Lee 2020 (62)	Yes	This study was funded by the National Heart, Lung, and Blood contract R01-HL-028226. Additionally, this work was supported by 5T32-HL-125232 (JWL and MEW) from the National Heart, Lung, and Blood Institute.	Yes	The authors declare that they have no known competing financial interests or personal relationships that could have appeared to influence the work reported in this paper.	No	
Lu 2024 (63)	Yes	This study was supported by Science and Technology Bureau of Jinhua City (Grant No. 2022-4-055).	Yes	None declared.	Available upon request	Data are available on reasonable request.
Marmol-Perez 2024 (64)	Yes	This study has been partially supported by the Spanish Ministry of Science and Innovation (ref. no. PID2020-117302RA-I00 financiado por MCIN/AEI/10.13039/501100011033), La Caixa Foundation (ref. no. LCF/BQ/PR19/11700007), the University of Granada Plan Propio de Investigación 2021-Excellence actions: Unit of Excellence on Exercise, Nutrition and Health (UCEENS), and the CIBEROBN, Centro de Investigación Biomédica en Red (CB22/03/00058), Instituto de Salud Carlos III, Ministerio de Ciencia e Innovación and Unión Europea-European Regional Development Fund. AM-P is recipient of a predoctoral fellowship (FPU20/05530) by the Spanish Ministry of Education, Culture and Sport. EU-G is supported by the Maria Zambrano fellowship by the Ministerio de Universidades y la Unión Europea-NextGenerationEU.	Yes	The authors declare no competing interests.	No	
Marshall 2022 (65)	Yes	This research received no specific grant from any funding agency in the public, commercial or not-for-profit sectors.	Yes	The authors declare that they have no competing interests.	Available upon request	The data that support the findings of this study are available from the corresponding author upon reasonable request.

Matricciani 2021 (66)	Yes	The Child Health CheckPoint has been supported to date by the National Health and Medical Research Council (NHMRC) of Australia (Project Grants 1041352, 1109355), The Royal Children's Hospital Foundation (2014-241), Murdoch Children's Research Institute, The University of Melbourne, National Heart Foundation of Australia (100660), Financial Markets Foundation for Children (2014-055, 2016-310), Cure Kids, New Zealand Ministry of Business, Innovation and Employment, University of Auckland Faculty Development Research Fund (3712987), National Centre for Longitudinal Data (at the DSS) and Victorian Deaf Education Institute. The urinary albumin and creatinine quantification was funded through NHMRC Program Grant 633003 Screening and Test Evaluation Program. The MCRI administered the research grants for the study and provided infrastructural support (IT and biospecimen management) to its staff and the study but played no role in the conduct or analysis of the trial. Senior Research Fellowships to MW (1046518) and DPB (1064629); Principal Research Fellowship to MW (1160906); Early Career Fellowship to KL (1091124). NHMRC Early Career Fellowship (APP1162166) to DD and Heart Foundation Post Graduate Fellowship (02084) to DD.	Yes	None.	No	
Mellow 2022 (67)	Yes	MM was supported by a Dementia Australia Research Foundation Ph.D. scholarship. The ACTIVate study is funded by an NHMRC Boosting Dementia Research Priority Round 5 grant (GNT1171313, $1.23m). DD was supported by an NHMRC Early Career Fellowship (GNT1162166). MG was supported by an Australian Research Council (ARC) fellowship (DE200100575). TS was supported by a Hospital Research Foundation grant (C-	Yes	The authors declare that the research was conducted in the absence of any commercial or financial relationships that could be construed as a potential conflict of interest.	Available upon request	The raw data supporting the conclusions of this article will be made available by the authors, without undue reservation.

		PJ-008-Transl-2020) awarded to AS and DD.				
Mellow 2024 (68)	Yes	MM was supported by a Dementia Australia Research Foundation PhD scholarship. The ACTIVate study is funded by an NHMRC Boosting Dementia Research Priority Round 5 grant (GNT1171313, $1.23 m). DD was supported by an NHMRC Early Career Fellowship (GNT1162166, 2019-2022) and an ARC Discovery Early Career Award (DE230101174, 2023-2025). MG was supported by an Australian Research Council (ARC) fellowship (DE200100575). TS was supported by a Hospital Research Foundation grant (C-PJ-008-Transl-2020) awarded to AS and DD. AS was supported by a Dementia Australia Henry Brodaty Mid-Career Fellowship. The funding bodies were not involved the design or conduct of the study; collection, analysis, and interpretation of the data; preparation, review, or approval of the manuscript; or the decision to submit the manuscript for peer-reviewed publication.	Yes	The authors declare that they have no competing interests.	Available upon request	The datasets generated and/or analysed during the current study are not publicly available due to the longitudinal nature of the larger ACTIVate study (ongoing at the time of publication) but are available from the corresponding author on reasonable request.

Migueles 2020 (69)	Yes	This is part of a PhD thesis conducted in the Official Doctoral Programme in Biomedicine of the University of Granada, Spain. This study was conducted under the umbrella of the ActiveBrains and the SmarterMove projects supported by the MINECO/FEDER (DEP2013-47540, DEP2016-79512-R, RYC-2011-09011). J.H.M. and L.V.T.-L. are supported by grants from the Spanish Ministry of Education, Culture and Sport (FPU15/02645 and FPU17/04802, respectively). C.C.-S. is supported by the Government of Andalusian, Integrated Territorial Initiative 2014-2020 for the province of Cádiz (PI-0002-2017) and the Spanish Ministry of Science and Innovation (FJC2018-037925-I).I.E.-C. is supported by a grant from the Alicia Koplowitz Foundation and by the Spanish Ministry of Economy and Competitiveness (RTI2018-095284-J-100). Additional funding was obtained from the University of Granada, PlanPropio de Investigación 2016, Excellence actions: Units of Excellence; Scientific Excellence Unit on Exercise andHealth (UCEES). Junta de Andalucía, Consejería de Conocimiento, Investigación y Universidades, and European Regional Development Funds (ref. SOMM17/6107/UGR). In addition, funding was provided by the SAMID IIInetwork, RETICS, funded by the PN I + D+I 2017-2021 (Spain), ISCIII-Sub-Directorate General for ResearchAssessment and Promotion, the European Regional Development Fund (ERDF) (Ref. RD16/0022), the EXERNET Research Network on Exercise and Health in Special Populations (DEP2005-00046/ACTI), and the European Union's 2020 research and innovation program under grant agreement No. 667302. The funders had no role in the design of the study; in the collection, analyses, or interpretation of data; in the writing of the manuscript, or in the decision to publish the results.	Yes	The authors declare no conflicts of interest.	No	

Migueles 2023 (70)	Yes	Open access funding provided by Karolinska Institute. The MINISTOP project was funded by the Swedish Research Council (project no. 2012-2883, Marie Löf), the Swedish Research Council for Health, Working Life and Welfare (2012-0906, Marie Löf; 2021-00036, Jairo H Migueles), Bo and Vera Axson Johnsons Foundation and Karolinska Institutet (Marie Löf, Jairo H Migueles), and the Joanna Cocozza Foundation (Marie Löf). Dorothea Dumuid was supported by the Australian National Health and Medical Research Council (NHMRC) Early Career Fellowship (APP1162166). The funders had no role in study design, data collection and analysis, decision to publish, or preparation of the manuscript.	Yes	The authors declare that they have no competing interests.	Available upon request	The datasets generated and/or analyzed during the current study are not publicly available but are available from the corresponding author on reasonable request.
Mitchell 2023 (71)	Yes	Medical Research Council (MR/N013867/1); British Heart Foundation (SP/15/6/31397, SP/F/20/150002)	Yes	None declared.	Yes	Original study protocol and survey documents can be found online at: https://bcs70.info/ and access to this data is available through the UK data service: UK Data Service>Series
Murphy 2024 (72)	Yes	This project had no funding but was kindly supported by the Allied Health and Human Performance Unit, University of South Australia. DD is supported by an Australian Research Council (ARC) Discovery Early Career Researcher Award (DECRA) DE230101174.	Yes	All the authors declare that they have no conflicts of interest	Available upon request	Raw data are available from authors on request.
Ng 2021 (73)	Yes	Supported by the National Health and Medical Research Council (Early Career Fellowships 1162166 to Dr Dumuid and 1091124 to Dr Lycett; Principal Research Fellowship 1160906 to Dr Wake). This work was also supported by the National Heart Foundation of Australia (Postdoctoral Fellowship 102084 to Dr Dumuid and Honorary Postdoctoral Fellowship 101239 to Dr Lycett). The CheckPoint study is supported by the National Health and Medical Research Council (project grants 1041352 and 1109355), The Royal Children's Hospital Foundation (2014-241), the Murdoch Children's Research Institute, The University of	Yes	The authors have indicated they have no financial relationships relevant to this article to disclose. The authors have indicated they have no potential conflicts of interest to disclose.	No	

		Melbourne, the National Heart Foundation of Australia (100660), and the Financial Markets Foundation for Children (2014-055 and 2016-310). Research at the Murdoch Children's Research Institute is supported by the Victorian Government's Operational Infrastructure Support Program.				
Niemelä 2024 (74)	Yes	NFBC1986 33-35y follow-up study received financial support from University of Oulu and Oulu University Hospital (K65760). The study was supported by the Ministry of Education and Culture (Grants OKM/20/626/2022, OKM/76/626/2022, OKM/68/626/2023). The study is also connected to the DigiHealth-project and 6GESS-project, strategic profiling projects at the University of Oulu, which are supported by the Academy of Finland (project numbers DigiHealth 326291, 6GESS 336449) and the University of Oulu.	Yes	The authors declare no conflicts of interest. The results of the study are presented clearly, honestly, and without fabrication, falsification, or inappropriate data manipulation.	Available upon request	NFBC data are available from the University of Oulu, Infrastructure for Population Studies. Permission to use the data can be applied for research purposes via an electronic material request portal. In the use of data, we follow the EU general data protection regulation (679/2016) and the Finnish Data Protection Act. The use of personal data is based on a cohort participant's written informed consent in their latest follow-up study, which may cause limitations to its use. Please, contact the NFBC project center (NFBCprojectcenter@oulu.fi) and visit the cohort website (www.oulu.fi/nfbc) for more information.
Oviedo-Caro 2020 (75)	Yes	The study was funded by Research Group CTS-948 of University Pablo de Olavide, Seville, Spain and European University of Madrid, Cátedra Real Madrid, Spain (funding project number P2017/RM08); this funding included material and equipment for the research. There was no external financial support. MAO is supported by the Departamento de Innovación, Investigación y Universidad del Gobierno de Aragón y el Fondo Europeo de Desarrollo Regional - Programa Operativo FEDER Aragón 2014-2020 "Construyendo Europa desde Aragón" (reference number PUI/2018-336). JB is supported by the Spanish Ministry of Education (grant number FPU13/05130) and by the Departamento de Innovación, Investigación y Universidad del Gobierno de Aragón y el Fondo Europeo de Desarrollo Regional -	No		No	

		Programa Operativo FEDER Aragón 2014-2020 "Construyendo Europa desde Aragón" (reference number PUI/2018-337).				
Powell 2020 (76)	Yes	Funding was provided by a University of Limerick Department of Physical Education and Sport Sciences Postgraduate Scholarship Programme (2013-2017) and the Health Research Board Centre for Health and Diet Research (HRB 2007/2013).	Yes	As per the signed conflicts of interest forms, Cormac Powell, Leonard D. Browne, Brian P. Carson, Kieran P. Dowd, Ivan J. Perry, Patricia M. Kearney, Janas M. Harrington and Alan E. Donnelly have no conflicts of interest to declare.	Available upon request	The data that support the findings of this study are available from the lead author (Cormac Powell; cormacpowell@swimireland.i.e.) upon reasonable request and with permission from all involved institutions.
Rasmussen 2023 (77)	Yes	This research was funded by research grants from the Czech Science Foundation (18-09188S and 22-02392S). DD was supported by the Australian National Health and Medical Research Council (NHMRC) Early Career Fellowship GNT1162166 and by the Centre of Research Excellence in Driving Global Investment in Adolescent Health funded by the NHMRC GNT1171981.	Yes	The authors declare that the research was conducted in the absence of any commercial or financial relationships that could be construed as a potential conflict of interest.	Yes	The dataset analysed during the current study is available in the Figshare repository, https://doi.org/10.6084/m9.figshare.20553108.v1
Rorem 2024 (78)	Yes	This research has been funded by the Stollery Children's Hospital Foundation's Health Research Institute. We thank the CHILD Cohort Study (CHILD) participant families for their dedication and commitment to advancing health research. CHILD was initially funded by CIHR and AllerGen NCE.	Yes	The authors declare that they have no known competing financial interests or personal relationships that could have appeared to influence the work reported in this paper.	Available upon request	Meta data is available by request through the National Coordinating Centre of the CHILD Study. Researchers must submit a concept proposal to the CHILDdb in order to receive participant level data.
Runacres 2023 (79)	Yes	This work was supported by the Knowledge Economy Skills Scholarships (KESS). KESS is a Pan-Wales higher-level skills initiative led by Bangor University on behalf of the HE sector in Wales. It is part funded by the Welsh Government's European Social Fund (ESF) convergence programme for West Wales and the Valleys.	Yes	The authors have declared that no competing interests exist.	Yes	All relevant data are within the paper and Supporting Information files.

Sandborg 2022 (80)	Yes	The following has provided funding for the HealthyMoms trial: the Swedish Research Council (2016-01147 to M. L.); the Swedish Research Council for Health, Working Life and Welfare (Forte, 2017-00088 to P. H.; 2018-01410 to M. L.); Bo and Vera Ax:son Johnsons' Foundation (to M. L.); the Strategic Research Area Health Care Science, Karolinska Institutet/Umeå University (to P. H.); the Swedish Society of Medicine (to P. H.); Karolinska Institutet (to P. H. and M. L.); Lions Forskningsfond (to P. H.); and ALF Grants, Region Östergötland (LIO-893101, LIO-941191 M. L.).	Yes	The authors declare no conflicts of interest. The results of the study are presented clearly, honestly, and without fabrication, falsification, or inappropriate data manipulation.	No	
Smith 2021 (81)	Yes	Dr AE Smith and MR Goldsworthy are funded by Australian NHMRC-ARC Dementia Research Development Fellowships (GNT1097397 and GNT1102272, respectively). Dr D Dumuid is funded by an NHMRC Early Career Fellowship (GNT1162166) and a Heart Foundation Postgraduate scholarship (102084).	Yes	Authors declare no conflict of interest.	No	
Smith 2021 (82)	No		No		No	
Song 2018 (83)	Yes	This work was supported in part by National Institute for Arthritis and Musculoskeletal Diseases (grant no. R01-AR054155 and P60-AR064464).	Yes	All authors report no disclosures or conflicts of interest.	No	
St Laurent 2023 (84)	Yes	This study was supported by NIH R01 HL111695 (PI: Spencer). CWS was supported by NIH F32 HD105384.	Yes	CLR serves as an editorial board member for the Journal of Activity, Sedentary and Sleep Behaviors. The remaining authors declare that they have no competing interests.	Available upon request	The datasets used and/or analyzed during the current study are available from the corresponding author on reasonable request.
Swindell 2020 (85)	Yes	The PREVIEW study received grants from the EU 7th Framework Programme (FP7-KBBE-2012), grant no: 312057; the New Zealand Health Research Council, grant no. 14/191; and the NHMRC-EU Collaborative Grant, Australia.	Yes	The authors declare that they have no competing interests.	Available upon request	The datasets generated during and/or analysed during the current study are not publicly available but are obtainable from the corresponding author on reasonable request.
Talarico 2018 (86)	Yes	This research was funded by a grant from the Heart and Stroke Foundation of Canada.	Yes	The authors declare that they have no conflict of interest.	No	

Tan 2023 (87)	Yes	The study is supported by the National Research Foundation (NRF) under the Open Fund-Large Collaborative Grant (OF-LCG; MOH-000504) administered by the Singapore Ministry of Health's National Medical Research Council (NMRC) and the Agency for Science, Technology and Research (ASTAR). In RIE2025, GUSTO is supported by funding from the NRF's Human Health and Potential (HHP) Domain, under the Human Potential Programme.	Yes	KMG reports being part of an academic consortium that has received research funding from Nestle Research, unrelated to the present manuscript. JYB is supported by a grant from the Agence Nationale de la Recherche (ANR) (iSCAN project, ANR-20-CE36-0001).	No	
Taylor 2018 (88)	Yes	The POI Study was funded by the Health Research Council of New Zealand (08/374, 12/281, 12/310) and the Southern District Health Board. RWT is supported by a Fellowship from Karitane Products Society. The funding source had no role in the design and conduct of the study; collection, management, analysis, or interpretation of the data; preparation, review, or approval of the manuscript; or the decision to submit the manuscript for publication.	Yes	The authors declare that they have no competing interests.	Available upon request	The datasets used and/or analysed during the current study are available from the corresponding author on reasonable request.
Taylor 2020 (89)	Yes	The PLAY Study was funded by the Health Research Council of New Zealand, and the Otago Diabetes Research Trust. VLF was in receipt of a Medicine Award and subsequently a Lottery Health Research New Zealand PhD Scholarship during her PhD study. RWT is partially funded by a Fellowship from the Karitane Products Society (KPS) Limited.	Yes	The authors declare that they have no conflict of interest.	No	
Taylor 2023 (90)	Yes	Health Research Council of New Zealand (08/374, 12/281, 12/310) and the Southern District Health Board. RWT is supported by a Fellowship from Karitane Products Society.	Yes	The authors declare they have no competing interests.	Available upon request	The datasets used and/or analysed during the current study are available from the corresponding author on reasonable request.
Tyler 2022 (91)	Yes	The current study was funded by The Waterloo Foundation (Grant #1669-3509). The Waterloo Foundation had no role in the design of the study, the collection, analysis, and interpretation of the data, and in the writing of the manuscript.	Yes	The authors declare that they have no conflict of interests. The results are presented clearly, honestly, and without fabrication, falsification, or inappropriate data manipulation.	Available upon request	The datasets used and/or analysed during the current study are available from the corresponding author on reasonable request.
Vanderlinden 2023 (92)	Yes	This research received no external funding.	Yes	The authors declare no conflict of interest.	Available upon request	The datasets used and/or analysed during the current study are available from

						the corresponding author on reasonable request.
Verhoog 2020 (93)	Yes	The Rotterdam Study is funded by Erasmus MC and Erasmus University, Rotterdam, The Netherlands; the Netherlands Organization for Scientific Research; the Netherlands Organization for Health Research and Development; the Research Institute for Diseases in the Elderly; the Ministry of Education, Culture and Science; the Ministry for Health, Welfare and Sports; the European Commission; and the Municipality of Rotterdam.	Yes	No financial disclosures were reported by the authors of this paper.	Available upon request	Data can be obtained on request. Requests should be directed toward the management team of the Rotterdam Study (secretariat.epi@erasmusmc.nl), which has a protocol for approving data requests. Because of restrictions based on privacy regulations and informed consent of the participants, data cannot be made freely available in a public repository.
Walmsley 2022 (94)	Yes	This research has been conducted using the UK Biobank Resource under Application Number 59070. CAPTURE-24 data collection and annotation was supported by the UK Economic and Social Research Council (grant number ES/L011662/1). RW is supported by a Medical Research Council Industrial Strategy Studentship (grant number MR/S502509/1). AD, DB, and KR are supported by the National Institute for Health Research (NIHR) Oxford Biomedical Research Centre (BRC). AD and SC are supported by Health Data Research UK (HDR UK), an initiative funded by UK Research and Innovation (UKRI), Department of Health and Social Care (England) and the devolved administrations, and leading medical research charities. AD and SC are supported by the Alan Turing Institute and the British Heart Foundation (grant number SP/18/4/33803). MW is supported by National Health and Medical Research Foundation of Australia grants 1080206 and 1149987. KR is supported by: the PEAK Urban programme, UKRI's Global Challenge Research Fund, Grant Ref: ES/P011055/; the Oxford Martin School (OMS), University of Oxford; and the British Heart Foundation, Grant ref: PG/18/65/33872. Computation used the Oxford Biomedical Research	Yes	MW is a consultant to Amgen, Kyowa Kirin and Freeline. No other authors have any competing interests to disclose	Yes	Data from the CAPTURE-24 study used for developing machine-learning models are available at 10.5287/bodleian:nZVqj2ojw. UK Biobank data are available to researchers who submit an application (https://www.ukbiobank.ac.uk/)

		Computing (BMRC) facility, a joint development between the Wellcome Centre for Human Genetics and the Big Data Institute supported by HDR UK and the NIHR Oxford BRC. The views expressed are those of the author(s) and not necessarily those of the UK National Health Service, the NIHR, the OMS, or the Department of Health and Social Care. The funding organisations had no role in design or conduct of the study; collection, management, analysis, and interpretation of the data; preparation, review, or approval of the manuscript; or decision to submit the manuscript for publication.				
